# PTEN-L inhibits mitophagy and GSDME-dependent pyroptosis via Parkin dephosphorylation

**DOI:** 10.1038/s44319-026-00957-4

**Published:** 2026-09-28

**Authors:** Chenghao Yan, Cainian Huang, Xu Liu, Yifan Liu, Yancheng Tang, Zhixuan Zhu, Jiaqi Qi, Hao Guo, Ying Li, Yuyuan Zhu, Lijie Wang, Yufei Luo, Linlin Xing, Haiji Wang, Faxiang Li, Bo Sun, Sulai Liu, Guang Lu, Haodong Xu, Bo Zhou, Han-Ming Shen, Liming Wang

**Affiliations:** 1 School of Biomedical Sciences, Hunan University, Changsha, China; 2 Shenzhen Research Institute, Hunan University, Shenzhen, China; 3 Center for Medical Genetics, Hunan Key Laboratory of Medical Genetics, Key Lab of Rare Pediatric Diseases of Ministry of Education, School of Life Sciences, Central South University, Changsha, China; 4 Cryo-EM Facility of Tsinghua University, Branch of National Protein Science Facility, Tsinghua University, Beijing, China; 5 Department of Hepatobiliary Surgery, The First Affiliated Hospital of Hunan Normal University, Hunan Provincial People’s Hospital, Changsha, China; 6 Zhongshan School of Medicine, Sun Yat-sen University, Guangzhou, China; 7 Department of Orthopaedics, The Second Xiangya Hospital of Central South University, Changsha, China; 8 Faculty of Health Sciences, University of Macau, Macau, China

**Keywords:** Autophagy & Cell Death, Organelles, Post-translational Modifications & Proteolysis

## Abstract

Mitochondrial dysfunction is a potent trigger of inflammatory cell death; however, the precise signaling pathways linking mitochondrial damage to pyroptosis remain incompletely understood. Here, we identify a previously unrecognized pathway in which mitochondrial depolarization activates the PINK1–Parkin axis to drive GSDME-mediated pyroptosis, a process negatively regulated by the phosphatase PTEN-L. Upon activation, Parkin promotes the ubiquitination and proteasomal degradation of MCL-1, facilitating mitochondrial translocation and activation of BAX. This triggers cytochrome c release, caspase-3 activation, and subsequent cleavage and plasma membrane targeting of GSDME, ultimately leading to pyroptotic cell death. Conversely, PTEN-L functions as a master negative regulator that counteracts Parkin through dephosphorylation and inactivation of Parkin. This action not only suppresses mitophagy but also stabilizes MCL-1, thereby inhibiting the downstream BAX/BAK–caspase-3–GSDME cascade and subsequent pyroptosis. Thus, our findings reveal a phosphorylation-dependent regulatory switch centered on Parkin that functionally couples mitophagy regulation to GSDME-dependent pyroptosis, delineating a novel mitochondrial signaling pathway that integrates organelle quality control with cellular fate decisions under stress conditions.

## Synopsis

Upon mitochondrial damage, Parkin-mediated degradation of MCL-1 triggers GSDME-dependent pyroptosis. PTEN-L dephosphorylates and inactivates Parkin, which stabilizes MCL-1 and concurrently inhibits both, pyroptosis and mitophagy.

- Mitochondrial depolarization activates the PINK1–Parkin axis to drive GSDME-dependent pyroptosis through Parkin-mediated ubiquitination and degradation of MCL-1, which unleashes the BAX/BAK–caspase-3 cascade.
- PTEN-L functions as a critical brake on this pathway by directly dephosphorylating Parkin and restraining its E3 ligase activity, thereby preserving MCL-1 and suppressing both mitophagy and pyroptosis.
- This phosphorylation-dependent regulatory switch centered on Parkin, couples organelle quality control to inflammatory cell death and expands the functional repertoire of the PINK1–Parkin axis beyond mitophagy.

## Abstract (web summary)

Upon mitochondrial damage, Parkin-mediated degradation of MCL-1 triggers GSDME-dependent pyroptosis. PTEN-L dephosphorylates and inactivates Parkin, which stabilizes MCL-1 and concurrently inhibits both, pyroptosis and mitophagy.

## Graphical abstract

**Figure:**
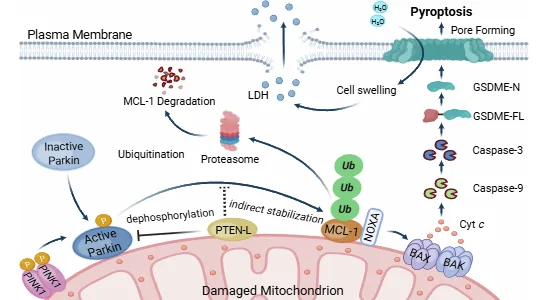

## Introduction

The E3 ubiquitin ligase Parkin, in concert with the kinase PINK1, plays a pivotal role in maintaining cellular homeostasis by eliminating damaged mitochondria through an autophagy-lysosome process termed mitophagy (Wang et al, 2020). Under homeostatic conditions, cytosolic Parkin maintains an autoinhibited state, with its catalytic activity suppressed by intramolecular interactions among its UBL, RING0, and REP domains (Ham et al, 2016; Trempe et al, 2013; Wauer and Komander, 2013). Mitochondrial depolarization leads to PINK1 stabilization on the outer mitochondrial membrane, where it phosphorylates ubiquitin (pSer65-Ub), thereby recruiting cytosolic Parkin (Kane et al, 2014; Kazlauskaite et al, 2014; Koyano et al, 2014; Lai et al, 2015; Wauer et al, 2015). Subsequently, PINK1 phosphorylates Parkin’s UBL domain at Ser65, which relieves autoinhibition and fully activating its E3 ligase activity (Gladkova et al, 2018; Kondapalli et al, 2012; Sauvé et al, 2015; Sauvé et al, 2018). This step is essential for Parkin function in vivo (Han et al, 2024; McWilliams et al, 2018). Since mutations in Parkin are linked to Parkinson’s disease characterized by dopaminergic neuron loss, Parkin is widely believed to exert cytoprotective effects. For example, Parkin can ubiquitinate BAX, BAK, and VDAC1 to suppress apoptosis (Bernardini et al, 2019; Cheng et al, 2023; Ham et al, 2020; Johnson et al, 2012). However, accumulating evidence indicates that Parkin also exhibits a context-dependent pro-death role. It can facilitate apoptosis through distinct mechanisms (Carroll et al, 2014; Liang et al, 2015; Quarato et al, 2023; Zhang et al, 2014), and its hyperactivation has been shown to contribute to synaptic dysfunction and neuronal death in early tauopathy via mediating Miro1 degradation (Jeong et al, 2022). This dual functionality, particularly in the regulation of cell death pathways beyond apoptosis, remains incompletely understood, highlighting the necessity to re-evaluate Parkin’s role in determining cell fate.

As a pivotal tumor suppressor, Phosphatase and Tensin Homolog (PTEN) exhibits both lipid and protein phosphatase activities (Lee et al, 2018). A longer isoform of PTEN, known as PTEN-Long (PTEN-L), contains an alternatively translated N-terminal region that enables distinct subcellular localization, particularly within the cytosol and mitochondria (Hopkins et al, 2013; Liang et al, 2014). Like canonical PTEN, PTEN-L retains an intact phosphatase domain with dual lipid and protein phosphatase activities. However, it targets a unique set of substrates compared to canonical PTEN (Li et al, 2019; Masson et al, 2016; Yin et al, 2024). We have previously reported that PTEN-L can directly dephosphorylate pSer65-Ub through its protein phosphatase activity, thereby inhibiting Parkin recruitment and mitophagy (Wang et al, 2018). Notably, in addition to its canonical role as a tumor suppressor, PTEN-L exhibits non-canonical functions: in certain contexts, it paradoxically promotes tumorigenesis in hepatocellular carcinoma (Shen et al, 2019), and protects tumors from T-cell-mediated killing by suppressing ferroptosis (Sun et al, 2021). These findings underscore the context-dependent nature of PTEN-L function and warrant further exploration of its regulatory roles in other cell death pathways.

Mitochondria are increasingly recognized as central signaling hubs that coordinate cell fate beyond their canonical roles in energy production and metabolism (Chakrabarty and Chandel, 2022; Picard and Shirihai, 2022). This is exemplified during intrinsic apoptosis, in which mitochondrial outer membrane permeabilization (MOMP), mediated by BAX/BAK pore proteins, facilitates the release of cytochrome c to activate the caspase cascade (Yuan and Ofengeim, 2024). The executioner caspase-3 then cleaves key substrates such as poly (ADP-ribose) polymerase (PARP), leading to the execution of apoptosis, a nonlytic form of cell death in which the cell retains membrane integrity (Yuan and Ofengeim, 2024). Gasdermin family proteins are the executioners of pyroptosis, a lytic and pro-inflammatory form of cell death that is distinct from apoptosis in both mechanism and morphology. Upon cleavage by caspases, their cytotoxic N-terminal domains form membrane pores, leading to cell lysis and death (Broz, 2025; Broz et al, 2019). Notably, caspase-3 can also cleave Gasdermin E (GSDME/DFNA5) at aspartate 270, releasing its N-terminal pore-forming domain (GSDME-N) (Rogers et al, 2017; Wang et al, 2017). Subsequently, GSDME-N translocates to the plasma membrane and undergoes oligomerization to form membrane pores, triggering pyroptosis (Rogers et al, 2017; Wang et al, 2017). Thus, GSDME cleavage switches caspase-3-mediated death from apoptotic to pyroptotic. Furthermore, GSDME-N can localize to the mitochondrial membrane, thereby promoting caspase-3 activation and establishing a feed-forward loop that amplifies cell death signals (Rogers et al, 2019). Although GSDME activation has been associated with mitochondrial damage in neurotoxin models (Neel et al, 2023), a critical unanswered question remains: whether and how the PINK1–Parkin axis, the primary pathway triggered by mitochondrial depolarization, participates in GSDME activation to coordinate pyroptotic cell death.

Here, we demonstrate that GSDME executes pyroptosis following mitochondrial depolarization, a process coordinated by the antagonistic regulators Parkin and PTEN-L. Activation of the PINK1-Parkin axis promotes MCL-1 degradation, leading to activation of the BAX/BAK–caspase-3–GSDME pathway and eventual induction of pyroptosis. Conversely, PTEN-L directly dephosphorylates Parkin, thereby suppressing mitophagy and stabilizing MCL-1, which in turn inhibits the downstream pyroptotic cascade. Collectively, our findings unveil a previously unrecognized regulatory mechanism in which PTEN-L, via dephosphorylating Parkin, simultaneously restricts mitophagy and pyroptosis, coupling these two processes under mitochondrial stress.

## Results

### PTEN-L restricts pyroptosis following mitochondrial depolarization

**Figure 1:**
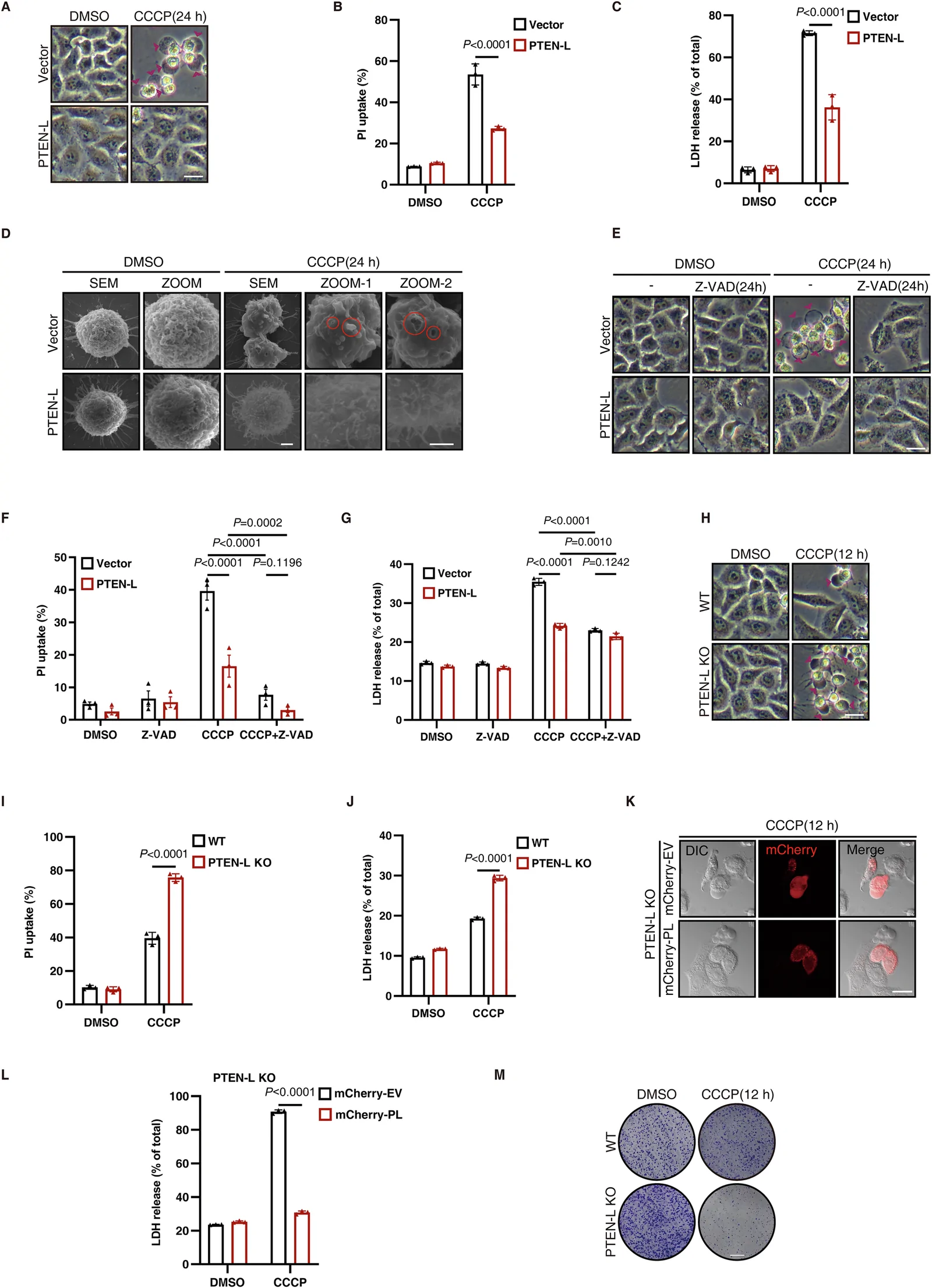
PTEN-L restricts pyroptosis following mitochondrial depolarization. (**A**–**C**) Representative microscopy images, PI uptake and LDH release assay of HeLa YFP-Parkin Vector/PTEN-L cells following treatment with 20 μM CCCP for 24 h. Scale bar, 25 μm. In (**B**) and (**C**), *P* < 0.0001. (**D**) Representative and zoom high-resolution scanning electron microscopy (SEM) images of HeLa YFP-Parkin Vector/PTEN-L cells following treatment with 20 μM CCCP for 24 h. Scale bars, 2 μm. (**E**–**G**) Representative microscopy images, PI uptake and LDH release assay of HeLa YFP-Parkin Vector/PTEN-L cells following treatment with 20 μM CCCP in combination with or without 40 μM Z-VAD-FMK (Z-VAD) for 24 h. Scale bar, 25 μm. In (**F**), *P* values were (from left to right): *P* < 0.0001; *P* < 0.0001; 0.0002; 0.1196. In (**G**), *P* values were (from left to right): *P* < 0.0001; *P* < 0.0001; 0.0010; 0.1242. (**H**–**J**) Representative microscopy images, PI uptake and LDH release assay of HeLa YFP-Parkin WT/PTEN-L KO cells following treatment with 20 μM CCCP for 12 h. Scale bar, 25 μm. In (**I**) and (**J**), *P* < 0.0001. (**K**, **L**) Representative microscopy images snapped by confocal microscopy and LDH release assay of HeLa YFP-Parkin PTEN-L KO cells rescued with mCherry empty control (mCherry-EV) or mCherry-PTEN-L (mCherry-PL) constructs following treatment with 20 μM CCCP for 12 h. Scale bar, 20 μm. In (**L**), *P* < 0.0001. (**M**) Colony formation assay of HeLa YFP-Parkin WT/PTEN-L KO cells following treatment with 20 μM CCCP for 12 h and 5 more days’ normal cultivation. Scale bar, 5 mm. Red arrows in (**A**), (**E**), and (**H**) indicate pyroptotic cells. Data in (**B**), (**C**), (**F**), (**G**), (**I**), (**J**), and (**L**) are presented as mean ± SD from three independent biological replicates. Statistical significance was assessed by two-way ANOVA. Source data are available online for this figure.

While investigating PTEN-L’s role in mitophagy (Wang et al, 2018), we observed that the mitochondrial uncoupler carbonyl cyanide m-chlorophenyl hydrazone (CCCP) induced pronounced cell death, consistent with previous reports (Bernardini et al, 2019; Carroll et al, 2014; Ham et al, 2020). Notably, CCCP-induced cell death in HeLa cells displayed morphological features that are reminiscent of pyroptosis, including cellular swelling and large plasma membrane bubbles, whereas PTEN-L overexpression (PTEN-L OE) significantly reversed CCCP-induced cell death (Fig. 1A; red arrows indicate cells with pyroptotic morphology). Furthermore, assessment of cell death by propidium iodide (PI) uptake (Fig. 1B), lactate dehydrogenase (LDH) release (Fig. 1C) and SYTOX green staining (Appendix Fig. S1A,B) revealed significantly reduced cell death in PTEN-L-overexpressing cells. Moreover, high-resolution scanning electron microscopy (SEM) and transmission electron microscopy (TEM) analysis of cellular morphology revealed that CCCP-treated HeLa cells displayed pyroptotic features. These features included numerous bubble-like protrusions on the cell surface (as indicated by red circles in Fig. 1D), visible membrane pores, plasma membrane rupture (as indicated by red circles in Fig. 1D), and cytoplasmic swelling (Appendix Fig. S1C), whereas PTEN-L OE attenuated these morphological changes (Fig. 1D; Appendix Fig. S1C). To investigate whether PTEN-L specifically suppresses pyroptosis triggered by mitochondrial depolarization, we treated HeLa cells with other stimuli known to induce pyroptosis, including tumor necrosis factor plus cycloheximide (TNFα plus CHX), Cisplatin, and Doxorubicin (Wang et al, 2017; Yu et al, 2021). However, in contrast to CCCP, PTEN-L exhibited no discernible effects on pyroptosis induced by these agents (Appendix Fig. S1D–G), suggesting that PTEN-L specifically modulated pyroptosis triggered by mitochondrial depolarization. Consistently, PTEN-L also suppressed pyroptosis triggered by oligomycin/antimycin A (O/A), another known mitochondrial depolarizing agent (Appendix Fig. S1H–L).

To elucidate the underlying mechanism, we performed mass spectrometry analysis and found that PTEN-L negatively regulated programmed cell death pathways (Appendix Fig. S2A). We then co-treated HeLa cells with ferroptosis inhibitor ferrostatin-1, necroptosis inhibitors necrostatin-1 and necrostatin 2 racemate (Nec-1s); however, these inhibitors failed to attenuate CCCP-induced cellular responses (Appendix Fig. S2B–G). In contrast, Z-VAD-FMK (Z-VAD) and Q-VD (Q-VD-OPh), two pan-caspase inhibitors, effectively suppressed CCCP or O/A-induced cell swelling and bubble protrusions (Fig. 1E; Appendix Fig. S2H,I), PI uptake (Fig. 1F; Appendix Fig. S2J), LDH release (Fig. 1G) and SYTOX green staining (Appendix Fig. S2K,L). It is well-established that caspase inhibitors can suppress both apoptosis and pyroptosis (Wang et al, 2017; Zhou et al, 2018). However, CCCP did not induce hallmark apoptotic features, including morphological changes (Appendix Fig. S3A,B), DNA laddering (Appendix Fig. S3C), nuclear fragmentation (Appendix Fig. S3D), PARP cleavage (Appendix Fig. S3E), or annexin V single-positive staining (Appendix Fig. S3F). Conversely, staurosporine (STS), a canonical inducer of apoptosis, robustly triggered apoptotic responses (Appendix Fig. S3A–E).

Using a PTEN-L knockout (KO) cell line we generated (Wang et al, 2018), we found that PTEN-L deficiency sensitized cells to CCCP-induced pyroptosis (Fig. 1H–J; Appendix Fig. S3G,H). Conversely, Re-introduction of PTEN-L into KO cells rescued the phenotype, mitigating cell swelling, bubble protrusions, and LDH release induced by CCCP or O/A (Fig. 1K,L; Appendix Fig. S3I–K). Strikingly, PTEN-L KO markedly enhanced the long-term cytotoxic effects of CCCP, as evidenced by reduced colony formation (Fig. 1M), underscoring its essential role in cell survival under mitochondrial stress.

### PTEN-L inhibits GSDME cleavage and activation

**Figure 2:**
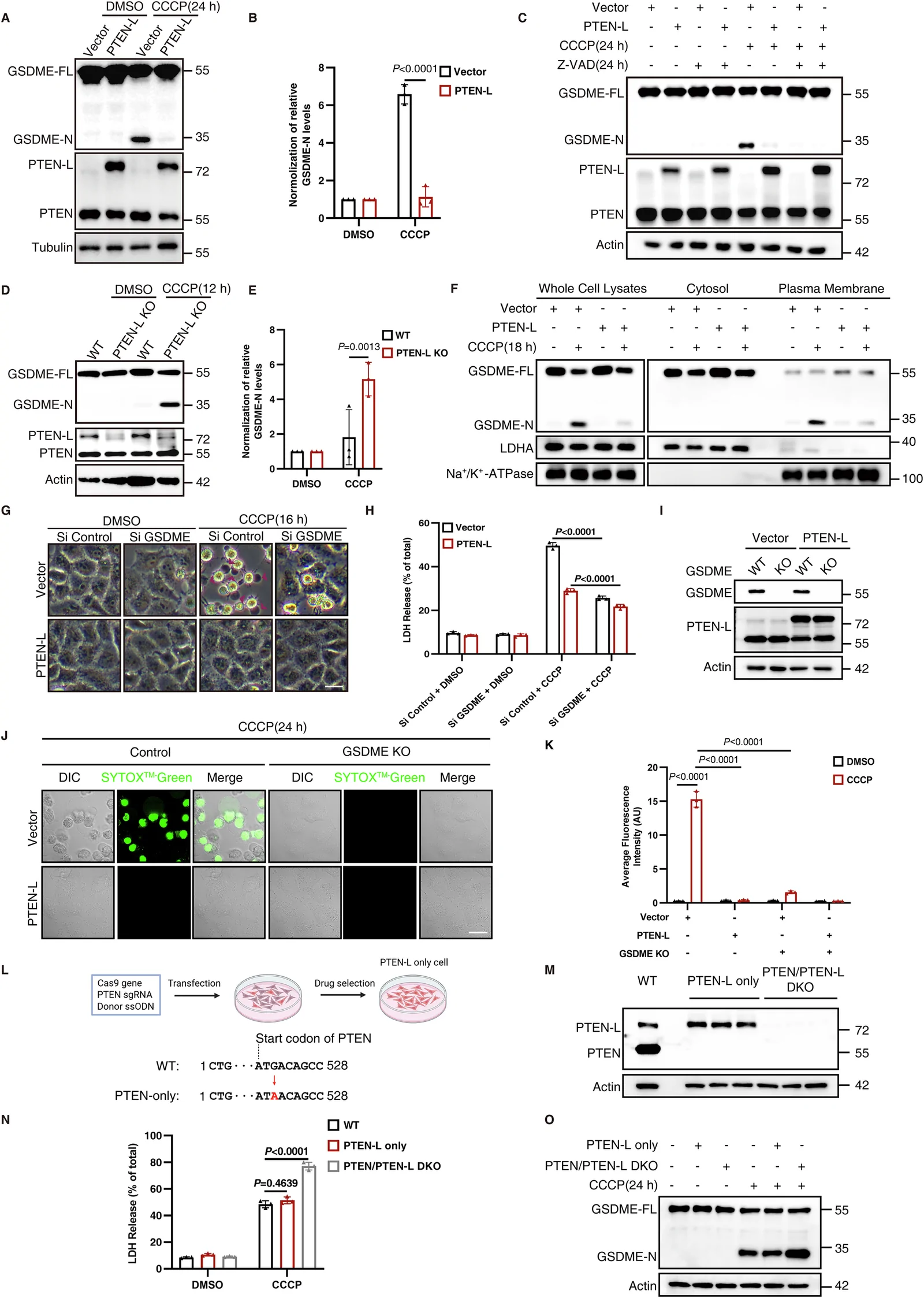
PTEN-L inhibits the cleavage, plasma membrane translocation and activation of GSDME. (**A**, **B**) Immunoblotting and quantitative analysis of GSDME cleavage and activation of HeLa YFP-Parkin Vector/PTEN-L cell lysates following treatment with 20 μM CCCP for 24 h. In (**B**), *P* < 0.0001. (**C**) Immunoblotting analysis of GSDME cleavage and activation in lysates of HeLa YFP-Parkin Vector/PTEN-L cells following treatment with 20 μM CCCP in combination or not with 40 μM Z-VAD for 24 h. (**D**, **E**) Immunoblotting and quantitative analysis of GSDME cleavage and activation in lysates of HeLa YFP-Parkin WT/PTEN-L KO cells following treatment with 20 μM CCCP for 12 h. In (**E**), *P* < 0.0001. (**F**) Immunoblotting detection of GSDME-N plasma membrane translocation of HeLa YFP-Parkin Vector/PTEN-L cells following treatment with 20 μM CCCP for 18 h, followed by cell fractionation. (**G**, **H**) Representative microscopy images and LDH release assay of HeLa YFP-Parkin Vector/PTEN-L cells transfected with nontargeting control siRNA (Si Control) or siRNA targeted against GSDME (Si GSDME) treated with 20 μM CCCP for 16 h. Scale bar, 25 μm. In (**H**), *P* < 0.0001. (**I**) HeLa YF*P-*Parkin Vector/PTEN-L cells with GSDME knockout (GSDME KO) created via CRISPR/Cas9 were subjected to immunoblotting as indicated. (**J**, **K**) Representative images and average fluorescence intensity (AU) of SYTOX Green staining in HeLa YFP-Parkin Vector/PTEN-L cells with GSDME knockout (GSDME KO) created via CRISPR/Cas9 system following treatment with 20 μM CCCP for 24 h. Scale bars, 20 μm. In (**K**), *P* values were (from left to right): *P* < 0.0001; *P* < 0.0001; *P* < 0.0001. (**L**) Schematic diagram of the CRISPR/Cas9 for generating the PTEN-L only cell line. (**M**) HeLa YFP-Parkin WT, HeLa YFP-Parkin PTEN-L only and HeLa YFP**-**Parkin PTEN/PTEN-L DKO cells created via CRISPR/Cas9 were subjected to immunoblotting as indicated. (**N**) LDH release assay of HeLa YFP-Parkin WT, HeLa YFP-Parkin PTEN**-**L only and HeLa YFP-Parkin PTEN/PTEN-L DKO cells following treatment with 20 μM CCCP for 24 h. *P* values were (from left to right): 0.4639; *P* < 0.0001. (**O**) Immunoblotting detection of GSDME cleavage and activation in lysates of HeLa YFP-Parkin WT, HeLa YFP-Parkin PTEN-L and PTEN/PTEN-L DKO cells following treatment with 20 μM CCCP for 24 h. Scale bar, 20 μm. Red arrows in (**G**) indicate pyroptotic cells. Immunoblots in (**C**), (**I**) are representative of two independent biological replicates. Immunoblots in (**A**), (**D**), (**F**), and (**O**) are representative of three independent biological replicates. Immunoblots in (**M**) are representative of four independent biological replicates. Data in (**B**), (**E**), (**H**), (**K**), and (**N**) are presented as mean ± SD from three independent biological replicates. Statistical significance was assessed by two-way ANOVA. Source data are available online for this figure.

Given the well-established role of gasdermin proteins, such as gasdermin D (GSDMD) and gasdermin E (GSDME), as key executors of pyroptosis (Liu et al, 2021), we investigated whether PTEN-L acts through these mediators. We found that PTEN-L OE significantly inhibited the cleavage of full-length GSDME into its active N-terminal fragment, GSDME-N, following CCCP or O/A treatment (Fig. 2A,B; Appendix Fig. S4A,B). This inhibitory effect was specific to mitochondrial stress, as PTEN-L did not alter GSDME cleavage induced by Cisplatin or Doxorubicin, and only slightly modulated it upon TNFα + CHX treatment (Appendix Fig. S4C,D). This modest effect during TNFα + CHX treatment is likely mediated via caspase-3 activation after the engagement of the mitochondrial pathway (Zhou et al, 2018). In contrast, neither CCCP nor O/A influenced GSDMD cleavage (Appendix Fig. S4E,F), highlighting the specificity for GSDME. And PTEN-L did not alter the mRNA level of GSDME (Appendix Fig. S4G). Consistent with the caspase-dependence nature of this process (Wang et al, 2017), the pan-caspase inhibitor Z-VAD effectively blocked CCCP-induced GSDME cleavage (Fig. 2C). We next investigated the role of endogenous PTEN-L in regulating GSDME activation, and found that genetic ablation of PTEN-L robustly enhanced CCCP-induced GSDME cleavage (Fig. 2D,E), an effect reversed by PTEN-L re-expression (Appendix Fig. S4H). Next, we conducted matched time-course experiments to compare PTEN-L OE, PTEN-L KO, and control HeLa cells after 12 h and 24 h CCCP treatment. At both time points, PTEN-L KO cells consistently displayed the highest level of pyroptosis, control cells exhibited intermediate levels, and PTEN-L OE cells showed the lowest levels, as evaluated by pyroptotic morphology, LDH release, and GSDME cleavage (Appendix Fig. S4I–K). These data confirm that PTEN-L plays a genuine regulatory role in pyroptosis across different time points. Crucially, CCCP treatment promoted GSDME-N translocation to the plasma membrane, a process that was markedly attenuated by PTEN-L (Fig. 2F), thereby confirming its functional inhibition of GSDME activation. To further substantiate the essential role of GSDME in this pathway, we employed reverse transfection for small interfering RNA (siRNA) to knock down GSDME expression (Appendix Fig. S5A), which led to a significant reduction in CCCP-induced pyroptosis (Fig. 2G) and decreased LDH release (Fig. 2H). Moreover, in GSDME KO cells, CCCP-induced pyroptosis was markedly suppressed (Fig. 2I–K; Appendix Fig. S5B–E). In contrast, siRNA-mediated knockdown of GSDMD did not alter the pyroptotic response triggered by CCCP (Appendix Fig. S5F,G). To definitively demonstrate that GSDME cleavage is required for pyroptosis, we performed rescue experiments in GSDME knockout cells by reintroducing either wild-type GSDME or a cleavage-resistant mutant (D267/270A). Following CCCP treatment, HeLa cells reconstituted with wild-type GSDME displayed pyroptotic morphology, elevated LDH release, and GSDME cleavage, whereas cells expressing the cleavage-resistant mutant showed none of these features (Appendix Fig. S5H–J). These results confirm that cleaved GSDME is strictly required for pyroptosis in our system.

To more precisely distinguish the functions of PTEN-L and canonical PTEN, we initially compared the exogenous overexpression of PTEN-L and PTEN. As the data indicated, PTEN-L OE significantly suppressed pyroptosis, whereas canonical PTEN OE did not inhibit any pyroptotic features (Appendix Fig. S6A–C). Subsequently, we explored the physiological relevance of endogenous PTEN-L in contrast to endogenous canonical PTEN. We established two additional HeLa cell lines using CRISPR/Cas9 technology. The first is a PTEN-L only cell line, where the ATG start codon of canonical PTEN was mutated to ATA, allowing the expression of PTEN-L but not canonical PTEN (Fig. 2L,M; Appendix Fig. S6D). The second is a PTEN/PTEN-L double KO cell line, in which both isoforms were eliminated by targeting a shared region (Fig. 2M). PTEN-L only and wild-type cells exhibited comparable levels of pyroptosis (Fig. 2N,O; Appendix Fig. S6E). In contrast, PTEN/PTEN-L double KO cells showed a significantly increased level of pyroptosis (Fig. 2N,O; Appendix Fig. S6E). These data strongly suggest that endogenous PTEN-L, rather than canonical PTEN, is responsible for the regulation of pyroptosis upon CCCP treatment.

Previous studies have demonstrated that GSDME is highly expressed in the human neuroblastoma SH-SY5Y cell line (Wang et al, 2017). To strengthen the physiological relevance and rule out potential cell line-specific artifacts, we initially investigated the sensitivity of SH-SY5Y cells to CCCP-induced pyroptosis at different concentrations (20 µM or 40 µM). SH-SY5Y cells expressing only endogenous Parkin exhibited weaker pyroptosis compared to SH-SY5Y cells stably expressing YFP-Parkin (Appendix Fig. S7A,B). To more efficiently detect the regulatory impact of PTEN-L in this cell line, we employed 40 µM CCCP to guarantee a robust pyroptotic response. In line with our findings in HeLa cells, PTEN-L OE in SH-SY5Y cells significantly repressed CCCP-induced pyroptosis and inhibited GSDME cleavage, while PTEN-L KO exacerbated these effects (Appendix Fig. S7C–H). Additionally, knockdown of GSDME in SH-SY5Y cells notably diminished CCCP-induced pyroptosis (Appendix Fig. S7I–K). Moreover, colony formation assays demonstrated that the absence of PTEN-L significantly augmented the long-term cytotoxicity of CCCP (Appendix Fig. S7L). Collectively, these data provide compelling evidence supporting the conclusion that PTEN-L suppresses pyroptosis primarily by inhibiting GSDME cleavage and activation.

### PTEN-L inhibits pyroptosis by suppressing BAX/BAK-mediated mitochondrial signaling pathway

**Figure 3:**
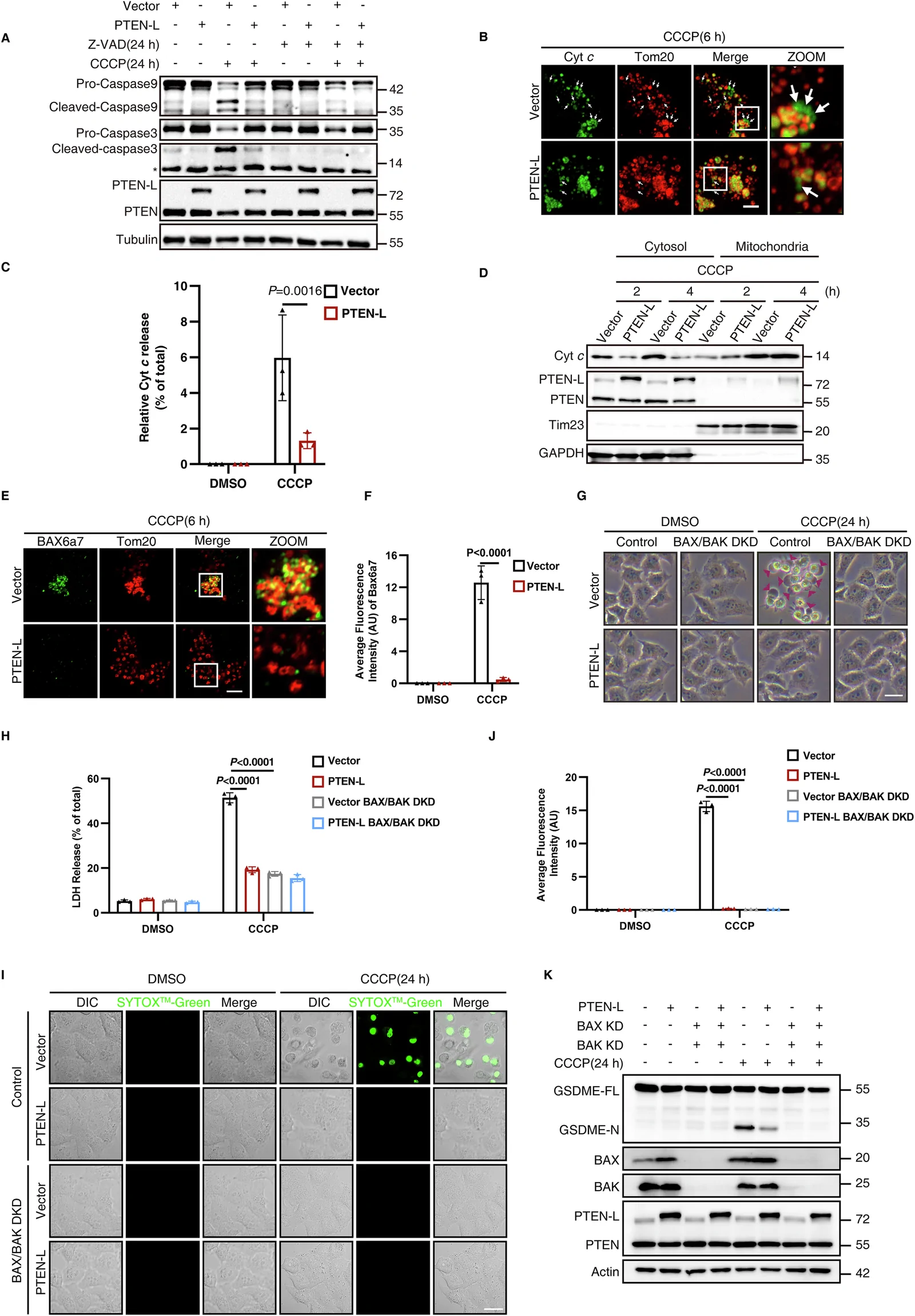
PTEN-L inhibits pyroptosis by suppressing BAX-mediated mitochondrial signaling pathway. (**A**) Immunoblotting analysis of caspase-9 and caspase-3 cleavage and activation in lysates of HeLa YFP-Parkin Vector/PTEN-L cells following treatment with 20 μM CCCP with or without with 40 μM Z-VAD for 24 h. * indicates non-specific bands. (**B**, **C**) Immunostaining assay and quantitative analysis to detect cytochrome c (Cyt c) release of HeLa YFP-Parkin Vector/PTEN-L cells following treatment with 20 μM CCCP for 6 h. White arrows indicate Cyt c releasing from mitochondria. Scale bar, 5 μm. In (**C**), *P* = 0.0016. (**D**) Immunoblotting detection of Cyt c release of HeLa YFP-Parkin Vector/PTEN-L cells following treatment with 20 μM CCCP for 6 h, followed by cell fractionation. (**E**, **F**) Immunostaining assay and quantitative analysis to assess the presence of active BAX (BAX6a7) on mitochondria of HeLa YFP-Parkin Vector/PTEN-L cells following treatment with 20 μM CCCP for 6 h. Scale bar, 5 μm. In (**F**), *P* < 0.0001. (**G**, **H**) Representative microscopy images, and LDH release assay of HeLa YFP-Parkin Vector/PTEN-L cells with BAX/BAK knockdown (BAX/BAK DKD) following treatment with 20 μM CCCP for 24 h. Scale bar, 25 μm. In (**H**), *P* values were (from left to right): *P* < 0.0001; *P* < 0.0001. (**I**, **J**) Representative images and average fluorescence intensity (AU) of SYTOX Green staining in HeLa YFP-Parkin Vector/PTEN-L cells with BAX/BAK knockdown (BAX/BAK DKD) following treatment with 20 μM CCCP for 24 h. Scale bars, 20 μm. In (**J**), *P* values were (from left to right): *P* < 0.0001; *P* < 0.0001. (**K**) Immunoblotting analysis for GSDME cleavage and activation of HeLa YFP-Parkin Vector/PTEN-L cells with BAX/BAK knockdown (BAX/BAK DKD) cell lysates following treatment with 20 μM CCCP for 24 h. Red arrows in (**G**) indicate pyroptotic cells. Immunoblots in (**A**) and (**D**) are representative of three independent biological replicates. Immunoblots in (**K**) are representative of four independent biological replicates. Data in (**C**), (**F**), (**H**), and (**J**) are presented as mean ± SD from three independent biological replicates. Statistical significance was assessed by two-way ANOVA. Source data are available online for this figure.

Since CCCP and O/A can activate caspase-3, an essential regulator of GSDME cleavage (Carroll et al, 2014; Lu et al, 2022; Quarato et al, 2023; Rogers et al, 2017; Wang et al, 2017), we next investigated how PTEN-L inhibits the caspase-3 activation. PTEN-L OE in HeLa cells effectively suppressed CCCP-induced cleavage of caspase-3 and its upstream activator, caspase-9, producing an effect comparable to that of Z-VAD treatment (Fig. 3A). Gene Ontology (GO) analysis of our proteomics data highlighted a strong association with mitochondrial components and biological processes (Appendix Fig. S8A), indicating a central role of mitochondria in regulating CCCP-induced cell death. Through mitochondrial fractionation from HeLa cells, we discovered that a portion of GSDME-N translocated to the mitochondria upon CCCP treatment, while PTEN-L OE inhibited this translocation (Appendix Fig. S8B). We also observed that CCCP treatment triggered the release of cytochrome c from mitochondria into the cytosol, a critical step for caspase activation, This effect was substantially reduced by PTEN-L, as demonstrated by immunofluorescence assays (Fig. 3B,C; Appendix Fig. S8C) and mitochondrial subcellular fractionation (Fig. 3D). Furthermore, flow cytometry analysis using digitonin-permeabilized cells corroborated these observations, showing consistent inhibition of cytochrome c release (Appendix Fig. S8D,E).

Given that BAX serves as a central mediator of MOMP and cytochrome c release (Bernardini et al, 2019; Carroll et al, 2014), we evaluated its activation using the 6A7 antibody, which specifically detects the active conformation of BAX. Treatment with CCCP in HeLa cells induced the translocation of activated BAX to mitochondria, forming distinct punctate structures, which was markedly inhibited by PTEN-L OE (Fig. 3E,F; Appendix Fig. S8F). Crucially, Overexpression of PTEN-L did not significantly alter the mRNA levels of BAX (Appendix Fig. S8G). BAX knockdown in HeLa cells and significantly reduced pyroptotic cell death (Appendix Fig. S8H,I) and prevented the cleavage of GSDME (Appendix Fig. S8J). Additionally, we examined whether BAK, another key factor known to regulate MOMP and cytochrome c release, participates in this pathway. Combined results from bright-field microscopy (Appendix Fig. S8K), SYTOX Green staining (Appendix Fig. S8L), and Western blotting (Appendix Fig. S8M) consistently showed that siRNA-mediated BAK knockdown in HeLa cells significantly attenuated CCCP-induced pyroptosis. To further confirm the importance of BAX and BAK in this process, we generated BAX/BAK double knockdown HeLa cells. Following CCCP treatment, we examined cell death morphology, LDH release, SYTOX Green uptake, and GSDME cleavage. BAX/BAK double knockdown completely blocked CCCP-induced pyroptosis (Fig. 3G-K), which verified that the mitochondrial pathway mediated by both BAX and BAK is crucial for the pyroptotic cell death.

BAX/BAK-mediated MOMP is a well-established event that typically activates the intrinsic apoptotic pathway. Indeed, in cells with knockout or knockdown of GSDME, we observed that CCCP treatment resulted in apoptosis rather than pyroptosis (Fig. 2G; Appendix Figs. S5B, 7J, 9A). These observations motivated us to examine the temporal relationship between MOMP and GSDME activation. We conducted a comprehensive time-course analysis examining cell morphology, PI uptake, LDH release, caspase-3 activity, and GSDME cleavage in HeLa cells. At early time points (6–9 h), we noted sporadic cells presenting apoptotic morphology, along with slight increases in PI uptake, LDH release, and caspase-3 activity. From 9 to 12 h, cells started to show pyroptotic morphology, with more significant increases in these parameters and the emergence of GSDME-N cleavage fragments. By 24 h, the majority of cells exhibited pronounced pyroptotic morphology, accompanied by robust GSDME cleavage and massively elevated pyroptotic markers (Appendix Fig. S9B–G). These results suggest that in the presence of GSDME, moderate mitochondrial damage initially triggers a low level of caspase-3 activity, which is sufficient to induce mild apoptosis. In contrast, severe or persistent damage elevates caspase-3 activity to a level that can efficiently cleave GSDME, thereby shifting the outcome towards pyroptosis. To further validate this model, we compared HEK293T cells, which lack endogenous GSDME, with SH-SY5Y cells, which express high levels of GSDME. CCCP treatment induced apoptosis in HEK293T cells, as evidenced by apoptotic morphology and PARP cleavage, whereas it triggered pyroptosis in SH-SY5Y cells (Appendix Fig. S9H–J). Importantly, the ectopic expression of GSDME in HEK293T cells transformed CCCP-induced cell death from apoptosis to pyroptosis, and this effect was inhibited by PTEN-L OE (Appendix Fig. S9K,L). These findings support the concept that the apoptosis-pyroptosis switch is context-dependent and regulated by the interaction between GSDME expression and the severity of mitochondrial damage.

Mitochondrial permeabilization can also lead to the generation of mitochondrial reactive oxygen species (mtROS). Several studies have demonstrated that mtROS function as both initiators and enhancers of GSDME-mediated pyroptosis (An et al, 2021; Zhang et al, 2026; Zhao et al, 2025). Oxidative stress triggers caspase-3 activation, leading to GSDME cleavage. The released N-terminal fragment subsequently targets mitochondria, further increasing mtROS production, exacerbating mitochondrial injury, and promoting plasma membrane pore formation. Consistent with previous observation, treatment with CCCP resulted in elevated mtROS levels, while PTEN-L OE attenuated mtROS production (Appendix Fig. S10A). Moreover, N-acetyl-L-cysteine (NAC), an ROS inhibitor, significantly reduced mtROS elevation (Appendix Fig. S10B). Noticeably, NAC also reversed the CCCP-induced pyroptosis as evidenced by the changes in cellular morphology (Appendix Fig. S10C), PI uptake (Appendix Fig. S10D), and most importantly GSDME cleavage (Appendix Fig. S10E).

Collectively, these findings establish PTEN-L as an upstream regulator of BAX/BAK activation within a mitochondrial signaling pathway that is essential for initiating the caspase cascade and subsequent GSDME-dependent pyroptosis.

### PTEN-L blocks the pyroptotic cascade by stabilizing MCL-1

Having established that PTEN-L acts upstream of BAX, we sought to elucidate the mechanism by which it prevents BAX activation. As pro-survival BCL-2 proteins (e.g., MCL-1, BCL-2, BCL-xL) are key repressors of BAX/BAK (Vandenabeele et al, 2023), we hypothesized that PTEN-L might regulate their stability. To identify potential targets, we performed proteomic analysis at an early time point after mitochondrial damage. Gene Set Enrichment Analysis (GSEA) revealed a negative correlation between PTEN-L expression and the activity of apoptotic signaling pathways (Appendix Fig. S11A). Given the well-documented overlap in upstream regulators shared between apoptosis and caspase-3/GSDME-mediated pyroptosis (Xu et al, 2021; Zhou et al, 2018), we reasoned that this apoptotic signature could serve as a surrogate indicator for identifying regulators involved in the pyroptotic phenotype we observed. Furthermore, our previous GO enrichment analysis highlighted the central involvement of mitochondria in this process. Therefore, by focusing on mitochondrial proteins through Venn analysis, we identified four core candidates: MCL-1, SOD2, HSPB1, and GPX1 (Appendix Fig. S11B). Notably, MCL-1 was the only BCL-2 family protein among them, making it a prime candidate for mediating PTEN-L’s effect on BAX.

**Figure 4:**
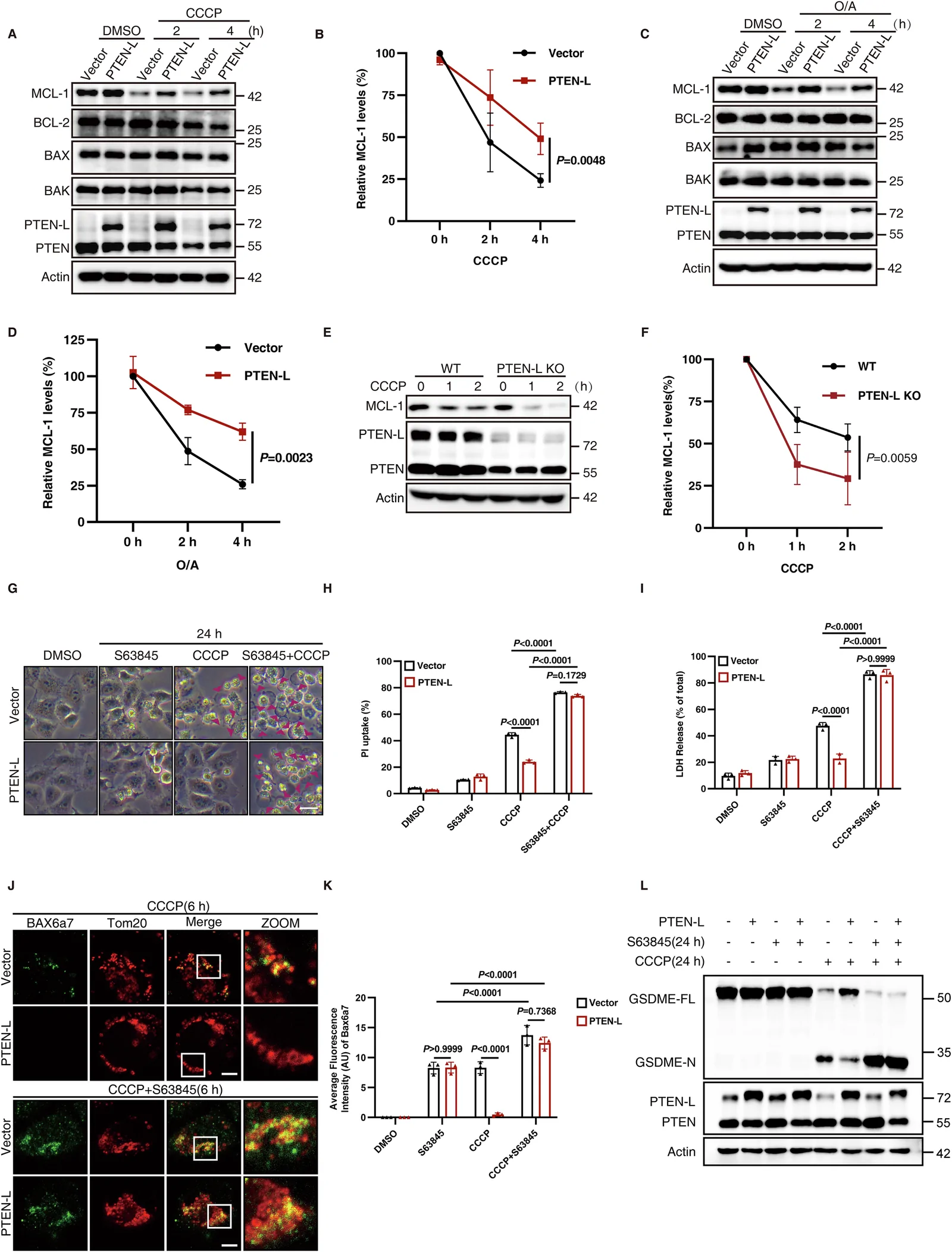
PTEN-L blocks the pyroptotic cascade by stabilizing MCL-1. (**A**, **B**) Immunoblotting and quantitative analysis of MCL-1 levels in lysates of HeLa YFP-Parkin Vector/PTEN-L cells following treatment with 20 μM CCCP for either 2 or 4 h. In (**B**), *P* = 0.0048. (**C**, **D**) Immunoblotting and quantitative analysis of MCL-1 levels in lysates of HeLa YFP-Parkin Vector/PTEN-L cell following treatment with 20 nM/200 nM O/A for either 2 or 4 h. In (**D**), *P* = 0.0023. (**E**, **F**) Immunoblotting and quantitative analysis of MCL-1 levels in lysates of HeLa YFP-Parkin WT/PTEN-L KO cells after following treatment with 20 μM CCCP for either 1 or 2 h. In (**F**), *P* = 0.0059. (**G**, **I**) Representative microscopy images, PI uptake assay, LDH release assay of HeLa YFP-Parkin Vector/PTEN-L cells treated with 20 μM CCCP in combination with or without 3 μM S63845 for 24 h. Scale bar, 25 μm. In (**H**), *P* values were (from left to right): *P* < 0.0001; *P* < 0.0001; *P* < 0.0001; 0.1729. In (**I**), *P* values were (from left to right): *P* < 0.0001; *P* < 0.0001; *P* < 0.0001; *P* > 0.9999. (**J**, **K**) Immunostaining assay and quantitative analysis to assess the presence of active BAX (BAX6a7) on mitochondria of HeLa YFP-Parkin Vector/PTEN-L cells following treatment with 20 μM CCCP in combination with or without 3 μM S63845 for 6 h. Scale bar, 5 μm. In (**K**), *P* values were (from left to right): *P* > 0.9999; *P* < 0.0001; *P* < 0.0001; *P* < 0.0001; 0.7368. (**L**) Immunoblotting analysis of GSDME cleavage and activation in lysates of HeLa YFP-Parkin Vector/PTEN-L cells following treatment with 20 μM CCCP combination with or without 3 μM S63845 for 24 h. Red arrows in (**G**) indicate pyroptotic cells. Immunoblots in (**A**), (**C**), and (**E**) are representative of three independent biological replicates. Immunoblots in (**L**) are representative of four independent biological replicates. Data in (**B**), (**D**), (**F**), (**H**), (**I**), and (**K**) are presented as mean ± SD from three independent biological replicates. Statistical significance was assessed by two**-**way ANOVA. Source data are available online for this figure.

We then found that CCCP or O/A treatment induced a pronounced degradation of MCL-1, but not of BCL-2, BAX, or BAK; this degradation was potently inhibited by PTEN-L OE (Fig. 4A–D). Moreover, knockout of PTEN-L facilitated CCCP-induced degradation of MCL-1 (Fig. 4E,F), positioning MCL-1 stability as a key event downstream of PTEN-L. To rule out the possibility that the differential MCL-1 degradation merely reflected the degree of cell death rather than a direct regulatory effect of PTEN-L, we treated HeLa cells with caspase inhibitor to block cell death and then examined MCL-1 levels under these conditions. Even when cell death was effectively inhibited, MCL-1 degradation in vector cells still remained significantly higher than that in PTEN-L OE cells (Appendix Fig. S11C,D), indicating that the protective effect of PTEN-L on MCL-1 stability is not simply a result of reduced cell death. To test the functional necessity of MCL-1, we co-treated HeLa cells with S63845, a potent and selective inhibitor of MCL-1 (Kotschy et al, 2016), and CCCP. Strikingly, S63845 completely abrogated PTEN-L-mediated cytoprotection, restoring pyroptosis as evidenced by alterations in cell morphology (Fig. 4G), PI uptake (Fig. 4H) and LDH release (Fig. 4I). Consistent results were observed when O/A was co-administered with S63845 (Appendix Fig. S11E–G). Furthermore, S63845 remarkably reversed PTEN-L’s suppression on BAX activation (Fig. 4J,K; Appendix Fig. S11H) and GSDME cleavage upon CCCP (Fig. 4L) and O/A treatment (Appendix Fig. S11I). To further confirm that the effects observed with S63845 are indeed attributable to the loss function of MCL-1, rather than off-target effects of the inhibitor, we generated stable MCL-1 knockdown HeLa cell lines and examined pyroptotic morphology, LDH release, SYTOX Green uptake, BAX activation, and GSDME cleavage. MCL-1 knockdown phenocopied the effects of S63845 treatment, which abolished PTEN-L-mediated cytoprotection and restored pyroptosis (Appendix Fig. S12A–G). Collectively, these data demonstrate that PTEN-L inhibits the pyroptotic cascade by stabilizing MCL-1, which in turn constrains BAX activation.

### PTEN-L suppresses Parkin-mediated ubiquitination and proteasomal degradation of MCL-1

**Figure 5:**
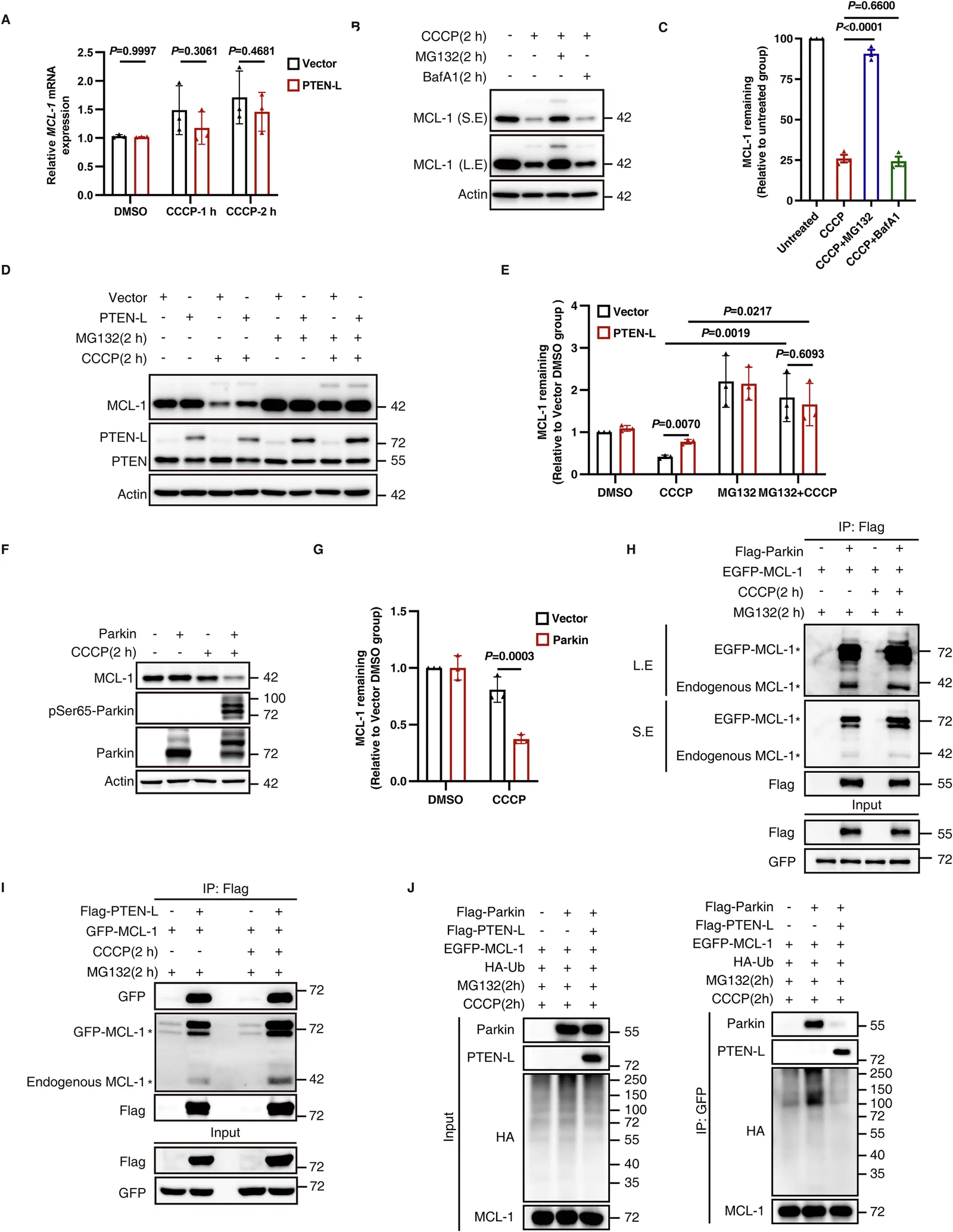
PTEN-L suppresses Parkin-mediated ubiquitination and proteasomal degradation of MCL-1. (**A**) Quantitative polymerase chain reaction to evaluate the relative mRNA expression of *MCL-1* genes in HeLa YFP-Parkin Vector/PTEN-L cells following treatment with 20 μM CCCP for either 1 or 2 h. *P* values were (from left to right): 0.9997; 0.3061; 0.4681. (**B**, **C**) Immunoblotting and quantitative analysis of HeLa YFP-Parkin cell lysates pretreated with 10 μM MG132 or 100 nM Bafilomycin A1 (BafA1) for 1 h following cotreatment with 20 μM CCCP for 2 h. S.E, short exposure. L.E, long exposure. In (**C**), *P* values were (from left to right): *P* < 0.0001; 0.6600. (**D**, **E**) Immunoblotting and quantitative analysis of HeLa YFP-Parkin Vector/PTEN-L cell lysates pretreated with 10 μM MG132 for 1 h following cotreatment with 20 μM CCCP for 2 h. In (**E**), *P* values were (from left to right): 0.0070; 0.0019; 0.0217; 0.6093. (**F**, **G**) Immunoblotting and quantitative analysis of MCL-1 levels in lysates of HeLa WT/YFP-Parkin cells following treatment with 20 μM CCCP for 2 h. In (**G**), *P* = 0.0003. (**H**) Immunoprecipitation analysis for the interaction between Parkin and MCL-1 in HEK293T cells transiently co-transfected with Flag-Parkin or Flag empty vector, and GFP-MCL-1 constructs pretreated with 10 μM MG132 for 1 h following cotreatment with 20 μM CCCP for 2 h. Flag-Parkin and Flag empty vector were immunoprecipitated with anti-Flag beads. S.E, short exposure. L.E, long exposure. (**I**) Immunoprecipitation analysis for the interaction between PTEN-L and MCL-1 in HEK293T cells transiently co-transfected with Flag-PTEN-L or Flag empty vector, and GFP-MCL-1 constructs pretreated with 10 μM MG132 for 1 h following cotreatment with 20 μM CCCP for 2 h. Flag-PTEN-L and Flag empty vector were immunoprecipitated with anti-Flag beads. (**J**) Immunoprecipitation analysis for ubiquitination of MCL-1 and interaction between PTEN-L, Parkin and MCL-1 in HEK293T cells transiently co-transfected with GFP-MCL-1, HA-Ub, Flag-Parkin, Flag-PTEN-L and empty vector constructs pretreated with 10 μM MG132 for 1 h following cotreatment with 20 μM CCCP for 2 h. GFP-MCL-1 was immunoprecipitated with anti-GFP beads. Immunoblots in (**B**), (**D**), and (**F**) are representative of three independent biological replicates. Immunoblots in (**H**), (**I**), and (**J**) are representative of two independent biological replicates. Data in (**A**), (**C**), (**E**), and (**G**) are presented as mean ± SD from three independent biological replicates. Statistical significance was assessed by two-way ANOVA (**A**), (**E**), (**G**) and two-tailed unpaired Student’s-t test (**C**). Source data are available online for this figure.

Having established MCL-1’s critical role, we explored the mechanism by which PTEN-L regulates its protein abundance. We initially assessed the mRNA levels of MCL-1 and found PTEN-L OE did not influence MCL-1 mRNA levels in HeLa cells; in fact, there was a slight decrease observed (Fig. 5A). To determine whether PTEN-L affects MCL-1’s protein degradation via the proteasomal or lysosomal pathway, we induced mitochondrial depolarization and applied specific inhibitors in HeLa cells. Co-treatment with the proteasome inhibitor MG132 effectively rescued CCCP-induced MCL-1 degradation, whereas the lysosome inhibitor Bafilomycin A1 (BafA1) did not (Fig. 5B,C). Importantly, MG132 treatment restored MCL-1 levels in control vector cells to a level comparable to that in PTEN-L overexpressing cells, indicating that PTEN-L stabilizes MCL-1 by specifically counteracting its proteasomal degradation. Moreover, the appearance of high-molecular-weight smears of MCL-1, which are characteristic of polyubiquitinated species, was enhanced upon MG132 treatment (Fig. 5B–E), further supporting the involvement of ubiquitin-proteasome system in MCL-1 turnover. We next investigated whether other regulators might contribute to MCL-1 degradation in this context. Notably, NOXA, a BH3-only protein known to promote proteasomal MCL-1 turnover (Djajawi et al, 2020), exhibited enhanced binding to MCL-1 and was upregulated upon CCCP treatment independently of PTEN-L expression (Appendix Fig. S13A–C). Knockdown of NOXA in HeLa cells attenuated MCL-1 degradation (Appendix Fig. S13D), decreased BAX activation (Appendix Fig. S13E,F), and inhibited pyroptosis (Appendix Fig. S13G–K), positioning NOXA upstream of PTEN-L in this pathway.

Subsequently, we investigated the E3 ligase accountable for MCL-1 ubiquitination within this system. Although Parkin is recognized to ubiquitinate MCL-1 under mitochondrial stress, the upstream regulatory factors that precisely modulate its E3 ligase activity remain poorly understood. We hypothesized that PTEN-L, as a phosphatase and binding partner of Parkin, could serve as such a regulator. Consistent with this notion, we found that CCCP treatment induced robust activation of Parkin, as evidenced by its phosphorylation at Serine 65 (pSer65-Parkin), and this activation was both temporally and functionally coupled to MCL-1 degradation (Fig. 5F,G). We also observed that PTEN-L overexpression did not completely block MCL-1 degradation, and a certain degree of degradation persisted even in the absence of Parkin, which implies the participation of additional E3 ligases. To test this, we treated HeLa cells with TAK-243, a specific inhibitor of the E1 ubiquitin-activating enzyme. As expected, TAK-243 completely blocked MCL-1 degradation regardless of the status of Parkin or PTEN-L (Appendix Fig. S14A–E), suggesting that PTEN-L mainly functions via Parkin. We then delineated the precise mechanism of PTEN-L’s intervention. Immunoprecipitation assays clearly demonstrated that Parkin interacted with both exogenous and endogenous MCL-1 (Fig. 5H; Appendix Fig. S14F). Likewise, immunoprecipitation assays indicated that PTEN-L also bound to exogenous and endogenous MCL-1 (Fig. 5I). Significantly, when Parkin, PTEN-L, and MCL-1 were co-expressed, PTEN-L effectively weakened the interaction between Parkin and MCL-1 (Fig. 5J; Appendix Fig. S14F). Even more importantly, PTEN-L potently reversed the Parkin-mediated ubiquitination of MCL-1 (Fig. 5J). These findings establish PTEN-L as a gatekeeper of Parkin-mediated MCL-1 ubiquitination by attenuating the interaction between Parkin and MCL-1 and suppressing Parkin-mediated ubiquitination of MCL-1, thereby stabilizing MCL-1 and preventing pyroptosis.

While the canonical role of MCL-1 is to inhibit apoptosis, our data reveal its critical function in restraining pyroptosis under specific conditions. We found that MCL-1 inhibition by S63845 alone induces apoptosis in our system, consistent with its established role in regulating the intrinsic apoptotic pathway. A pivotal shift to pyroptosis occurs only when MCL-1 inhibition is coupled with a parallel insult that activates the PINK1-Parkin axis, as achieved with CCCP co-treatment in HeLa cells (Appendix Fig. S15A,B). In comparison with the treatment of CCCP or S63845 alone, their combination uniquely drove robust Parkin mitochondrial recruitment and activation (Appendix Fig. S15C,D), accompanied by extensive mitochondrial fragmentation (Appendix Fig. S15E,F). We also found MCL-1 knockdown promoted Parkin mitochondrial recruitment and activation, which is consistent with previous study (Hollville et al, 2014a). To explore whether broader pharmacological inhibition of other BCL-2 family members could also sensitize cells to pyroptosis, we employed ABT-737, a BH3 mimetic that inhibits BCL-2 and BCL-xL but not MCL-1 in HeLa cells (van Delft et al, 2006). Consistent with our findings and earlier studies, ABT-737 and/or S63845 alone induced apoptosis (Appendix Fig. S15I,J). When combined with CCCP, S63845 promoted a shift toward pyroptosis, whereas ABT-737 did not further enhance pyroptosis beyond CCCP treatment alone (Appendix Fig. S15I,K,L). Importantly, ABT-737 did not reverse the protective effect of PTEN-L (Appendix Fig. S15I,K,L). These results further support the specificity of MCL-1 in mediating the effects we observed. However, it is important to note that the failure of S63845 alone to induce pyroptosis might be dependent on experimental conditions. It is still formally plausible that under specific circumstances, for example, when S63845 leads to sufficiently severe mitochondrial damage, the resultant caspase-3 activity could reach the threshold necessary for GSDME cleavage and pyroptosis induction. In addition, to investigate the role of mitophagy in this process, we utilized a penta KO HeLa cell line which is deficient in mitophagy. We found the presence and activation of Parkin but not the activity of mitophagy is essential for CCCP-induced pyroptosis (Appendix Fig. S15M,N).

Based on these findings, we propose a threshold model in which the apoptosis-pyroptosis switch is regulated by the extent of mitochondrial damage, the level of caspase-3 activation, and the expression of GSDME. MCL-1 degradation acts as an initiating event, facilitating BAX/BAK-mediated MOMP while generating only a relatively low level of caspase-3 activity that is sufficient for apoptosis. A boosting signal, provided by the Parkin-driven amplification of mitochondrial damage, elevates caspase-3 activity beyond the critical threshold required for GSDME cleavage and pyroptosis. Notably, Parkin has dual functions: under mild stress, it promotes mitophagy and cell survival; under severe stress, it amplifies mitochondrial damage to induce pyroptosis. The exact threshold for GSDME cleavage is likely to vary according to the cellular context, including GSDME expression levels, caspase-3 abundance, and the kinetics of mitochondrial damage. Such context-dependence might account for the variable outcomes observed across different cell lines and experimental conditions. This model elucidates why MCL-1 inhibitors alone induce apoptosis in our system, while their combination with mitochondrial depolarizing agents triggers pyroptosis, and highlights the importance of signal intensity in cell fate decisions.

### PTEN-L is a direct protein phosphatase for Parkin and dephosphorylates it at Serine 65

**Figure 6:**
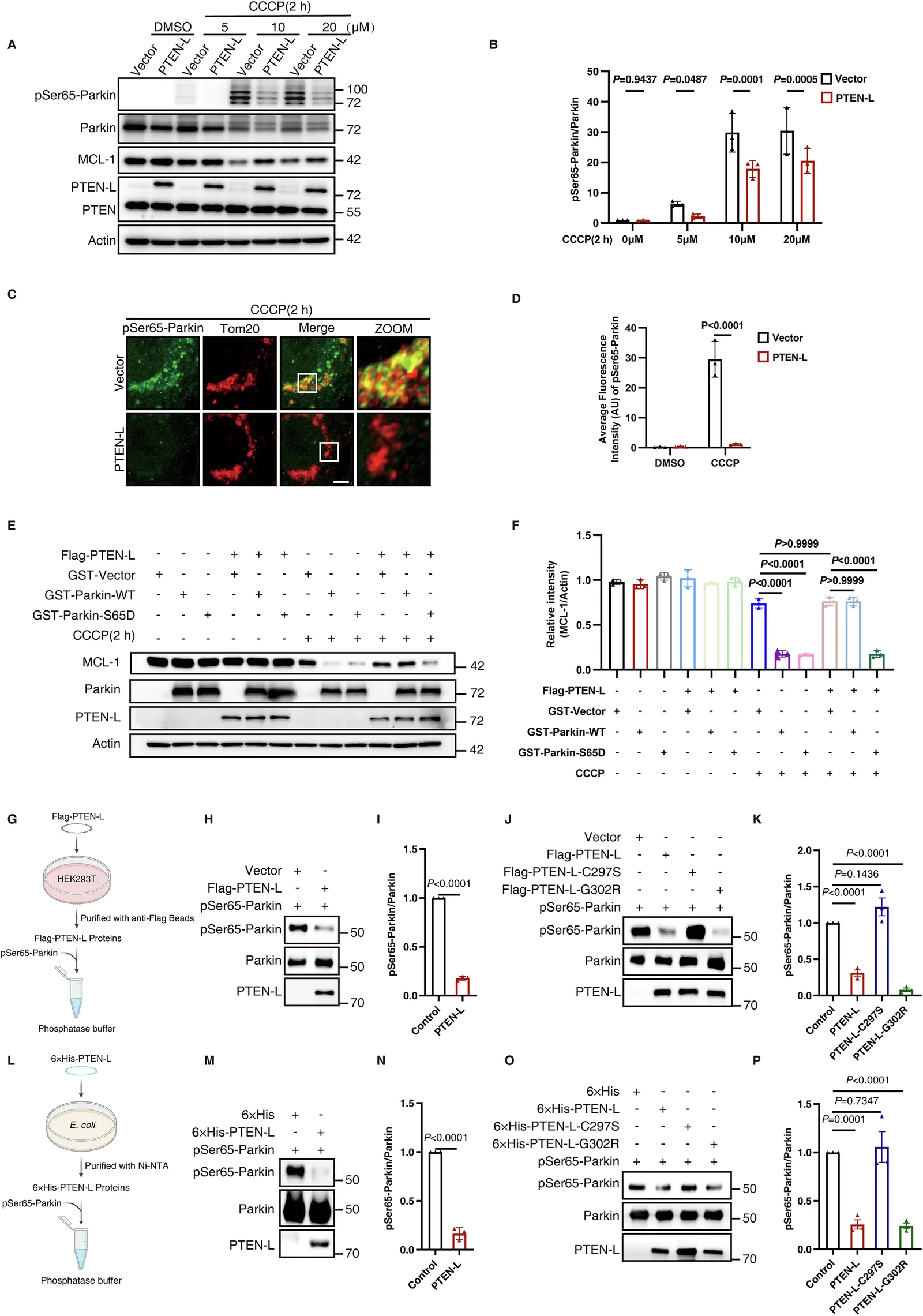
PTEN-L is a direct protein phosphatase for Parkin and dephosphorylates it at Serine 65. (**A**, **B**) Immunoblotting analysis and quantitative analysis of pSer65-Parkin levels in lysates of HeLa YFP-Parkin Vector/PTEN-L cells following treatment with 5 μM, 10 μM or 20 μM CCCP for 2 h. In (**B**), *P* values were (from left to right): 0.9437; 0.0487; 0.0001; 0.0005. (**C**, **D**) Immunostaining assay and quantitative analysis of pSer65-Parkin levels in lysates of HeLa YFP-Parkin Vector/PTEN-L cells following treatment with 20 μM CCCP for 2 h. Scale bar, 5 μm. In (**D**), *P* < 0.0001. (**E**, **F**) Immunoblotting and quantitative analysis of MCL-1 levels in lysates of Hela cells transiently co-transfected with Flag-PTEN-L, GST empty vector, GST-Parkin-WT, or GST-Parkin-S65D constructs following treatment with 20 μM CCCP for 2 h. In (**F**), *P* values were (from left to right): *P* < 0.0001; *P* < 0.0001; *P* > 0.9999; *P* > 0.9999; *P* < 0.0001. (**G**) Schematic representation showing in vitro dephosphorylation assay using Flag-PTEN-L proteins purified from HEK293T cells transfected with different constructs of Flag-PTEN-L. (**H**, **I**) Immunoblotting and quantitative analysis of in vitro dephosphorylation assay samples of purified pSer65-Parkin incubated with purified Flag-PTEN-L proteins and Flag control proteins from HEK293T cells in phosphatase reaction buffer. pSer65-Parkin was evaluated by anti-pSer65-Parkin specific antibody (MJF-17). In (**I**), *P* < 0.0001. (**J**, **K**) Immunoblotting and quantitative analysis of in vitro dephosphorylation assay samples of purified pSer65-Parkin incubated with purified Flag-WT PTEN-L, Flag-PTEN-L^C297S^, Flag-PTEN-L^G302R^ proteins and Flag control proteins from HEK293T cells in phosphatase reaction buffer. pSer65-Parkin was evaluated by anti-pSer65-Parkin specific antibody (MJF-17). In (**K**), *P* values were (from left to right): *P* < 0.0001; 0.1436; *P* < 0.0001. (**L**) Schematic representation showing in vitro phosphatase assay using 6×His-PTEN-L proteins purified from *E. coli* transformed with different constructs of 6×His-PTEN-L. (**M**, **N**) Immunoblotting and quantitative analysis of in vitro dephosphorylation assay samples of purified pSer65-Parkin incubated with purified 6×His-PTEN-L proteins and His control proteins from *E. coli* in phosphatase reaction buffer. pSer65-Parkin was evaluated by anti-pSer65-Parkin specific antibody (MJF-17). In (**N**), *P* < 0.0001. (**O**, **P**) Immunoblotting and quantitative analysis of in vitro dephosphorylation assay samples of purified pSer65-Parkin incubated with purified 6×His-WT PTEN-L, 6×His-PTEN-L^C297S^, 6×His -PTEN-L^G302R^ proteins and His control proteins from *E. coli* in phosphatase reaction buffer. pSer65-Parkin was evaluated by anti-pSer65-Parkin specific antibody (MJF*-*17). In (**P**), *P* values were (from left to right): 0.0001; 0.7437; *P* < 0.0001. Immunoblots in (**A**), (**H**), (**J**), (**M**), and (**O**) are representative of three independent biological replicates. Immunoblots in (**E**) are representative of four independent biological replicates. Data in (**B**), (**D**), (**F**), (**I**), (**K**), (**N**), and (**P**) are presented as mean ± SD from three independent biological replicates. Statistical significance was assessed by two-way ANOVA (**B**), (**D**) and two-tailed unpaired Student’s-t test (**F**), (**I**), (**K**), (**N**), (**P**). Source data are available online for this figure.

The PINK1-mediated phosphorylation of Parkin at Serine 65 (pSer65-Parkin) is a pivotal event in mitochondrial quality control, serving as a key trigger for Parkin activation and subsequent mitophagy. Our previous studies demonstrated that PTEN-L suppresses mitophagy by dephosphorylating the PINK1-generated phospho-ubiquitin (pSer65-Ub). In this study, we examined whether PTEN-L also exerts a more direct regulatory effect on Parkin itself. We first confirmed that PTEN-L OE significantly reduced pSer65-Parkin levels across a broad range of CCCP concentrations, as demonstrated by immunoblotting (Fig. 6A,B) and immunostaining (Fig. 6C,D; Appendix Fig. S16A). The abundance of MCL-1 exhibited a positive correlation with PTEN-L expression and an inverse correlation with pSer65-Parkin levels (Fig. 6A). To determine whether PTEN-L directly stabilizes MCL-1 or acts indirectly through Parkin, we expressed PTEN-L either in the presence or absence of Parkin in HeLa cells. In the absence of Parkin, MCL-1 degradation occurred regardless of PTEN-L expression, indicating that PTEN-L does not directly stabilize MCL-1 (Appendix Fig. S16B,C). However, when Parkin was present, PTEN-L expression reduced Parkin phosphorylation and suppressed MCL-1 degradation (Appendix Fig. S16B,C). Moreover, PTEN-L failed to inhibit the phospho-mimetic Parkin S65D mutant, which cannot be dephosphorylated (Fig. 6E,F). These results indicate that PTEN-L indirectly stabilizes MCL-1 via dephosphorylation and inactivation of Parkin. As expected, PTEN-L effectively suppressed mitophagy in HeLa cells, as assessed by the mitochondria-targeted monomeric keima-red (mt-Keima) assay (Appendix Fig. S16D,E). Additionally, we observed that PTEN-L OE markedly reduced pSer65-Parkin following exposure to varying concentrations or durations of O/A (Appendix Fig. S16F–H). Conversely, genetic ablation of PTEN-L robustly enhanced pSer65-Parkin induction (Appendix Fig. S16I). Notably, the increase in pSer65-Parkin was specifically associated with mitochondrial depolarization and was not observed upon treatment with other cell death inducing agents (Appendix Fig. S16J).

We next sought to elucidate the mechanism by which PTEN-L reduces pSer65-Parkin. We first excluded an effect on the upstream kinase PINK1, as PTEN-L did not alter PINK1’s mitochondrial accumulation (Appendix Fig. S17A) or its autophosphorylation status (Appendix Fig. S17B) in HeLa cells. We then observed a context-dependent influence on Parkin recruitment: PTEN-L impaired mitochondrial translocation of Parkin under low-dose CCCP treatment (Appendix Fig. S17C), but this inhibitory effect was lost under high-dose stress (Appendix Fig. S17D). This differential regulation mirrors our earlier observation that PTEN-L-mediated dephosphorylation of pSer65-Ub is more pronounced at lower stress levels (Appendix Fig. S17E,F). Together, these findings suggest that PTEN-L employs a dual-layer inhibitory mechanism, attenuating the initial ubiquitin signal during mild stress and directly counteracting Parkin activation under conditions of strong stimulation.

This observation led us to hypothesize that PTEN-L might act as a direct phosphatase for Parkin. To rigorously test this hypothesis, we purified human WT PTEN-L protein, along with its lipid phosphatase-dead (PTEN-L^G302R^) and protein phosphatase-dead (PTEN-L^C297S^) mutants, from HEK293T cells (Fig. 6G). In an in vitro dephosphorylation assay, wild-type PTEN-L significantly reduced the phosphorylation level of pSer65-Parkin (Fig. 6H,I). This dephosphorylation activity was abolished by the protein phosphatase-dead mutation (PTEN-L^C297S^), but remained intact in the lipid phosphatase-dead mutant (PTEN-L^G302R^) (Fig. 6J,K), thereby demonstrating that PTEN-L’s protein phosphatase activity is essential for this function. To rule out potential contamination from co-purifying mammalian phosphatases, we repeated the experiment using highly purified recombinant proteins expressed in *E. coli* (Fig. 6L; Appendix Fig. S17G). The results were clear and consistent: only wild-type PTEN-L, and not the C297S mutant, directly dephosphorylated pSer65-Parkin (Fig. 6M–P).

Therefore, our work conclusively establishes PTEN-L as a direct protein phosphatase for Parkin. These findings elucidate the mechanism underlying PTEN-L’s potent suppression of the pyroptotic cascade and unveils a sophisticated, two-tiered phosphatase strategy: PTEN-L initially attenuates the initiating signal (pSer65-Ub), and subsequently, under sustained stress, directly inactivates the pathway’s core amplifier (pSer65-Parkin), thereby ensuring precise and robust control over cell fate.

## Discussion

**Figure 7:**
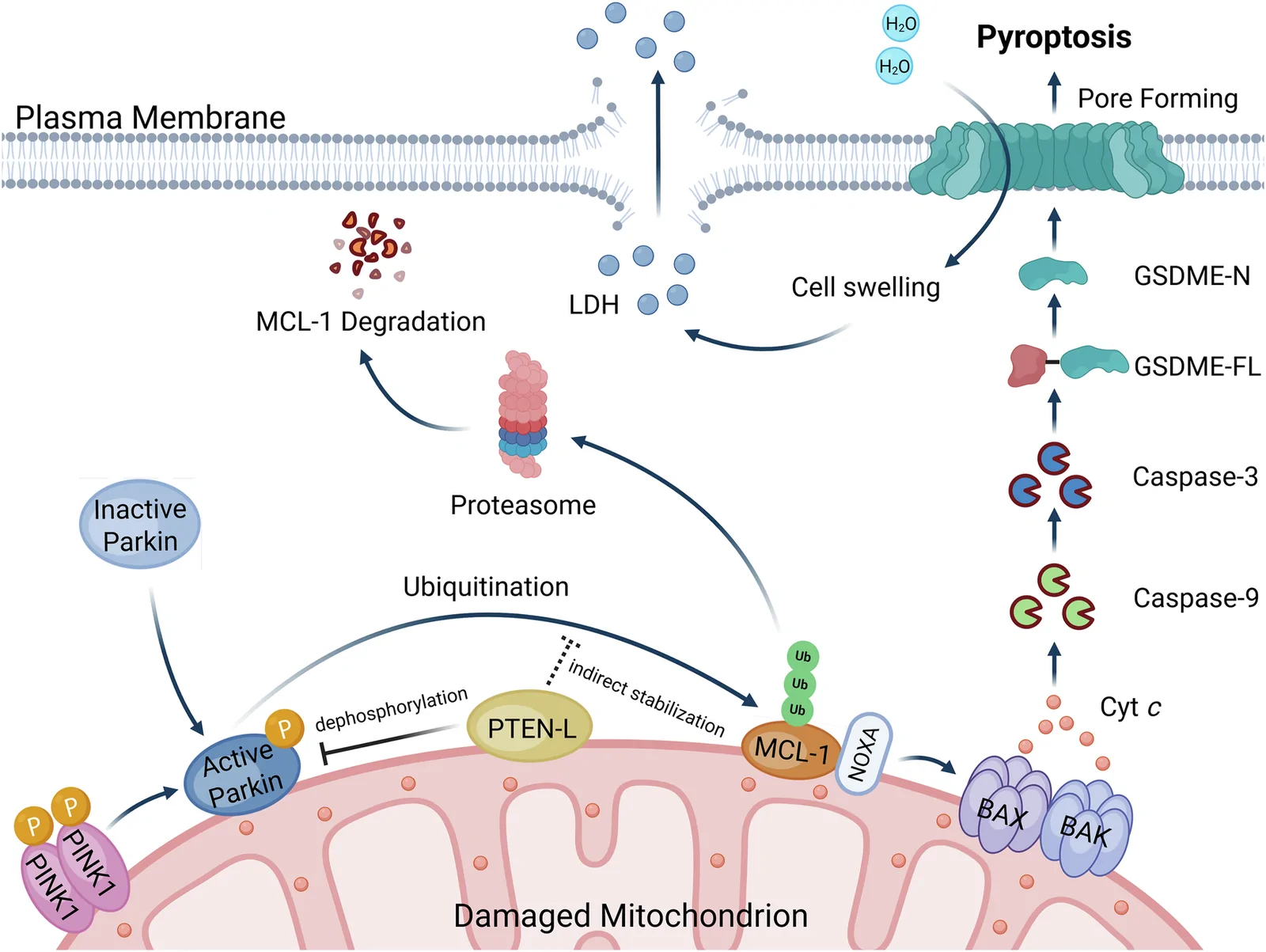
Illustration of the proposed mechanism of pyroptosis pathway. Following mitochondrial damage, PINK1 phosphorylates and activates Parkin. Once activated, Parkin ubiquitinates MCL-1 and designates it for proteasomal degradation. NOXA promotes this degradation process by binding to MCL-1, thereby accelerating its turnover. The subsequent depletion of MCL-1 relieves its inhibitory impact on BAX/BAK, leading to their recruitment to and activation on the mitochondria. Active BAX/BAK enables cytochrome c to be released from the mitochondria into the cytosol, which in turn activates caspase-3. Ultimately, this process induces pyroptosis by facilitating GSDME cleavage and plasma membrane localization. In contrast, PTEN-L directly dephosphorylates Parkin and suppresses its E3 ligase activity, thus indirectly stabilizing MCL-1. This in turn prevents the activation of the BAX/BAK-caspase-GSDME pathway and inhibits pyroptosis.

In this study, we elucidate a comprehensive mitochondrial pathway that converts depolarization stress into pyroptotic cell fate, governed by the antagonistic regulators Parkin and PTEN-L (Fig. 7). We demonstrate that upon mitochondrial depolarization, activation of the PINK1-Parkin axis not only initiates mitophagy but also triggers pyroptosis by promoting ubiquitination and degradation of MCL-1, leading to BAX/BAK/caspase-3-dependent cleavage of GSDME. Conversely, we identify the phosphatase PTEN-L as a master negative regulator of this pathway. PTEN-L counteracts Parkin through dephosphorylation and inactivation of Parkin, thereby stabilizing MCL-1 and preserving mitochondrial integrity, which in turn suppresses the pyroptotic cascade. Our findings establish PTEN-L as a critical gatekeeper in orchestrating cellular responses to mitochondrial stress, uncovering a previously unrecognized phospho-switch that simultaneously inhibits mitophagy and pyroptosis via direct regulation of Parkin.

Mitochondrial stresses that induce mitochondrial damage or depolarization can trigger mitophagy and mitochondria-related cell death. While the involvement of Parkin in mitophagy is well-established, its impact on cell fate remains intricate and controversial. Previous studies have reported that Parkin mediates BAX ubiquitination to prevent its translocation to mitochondria or promote its degradation, thereby inhibiting apoptosis (Cakir et al, 2017; Johnson et al, 2012). Additionally, it has been demonstrated that Parkin inhibits apoptosis by enhancing NEMO ubiquitination, leading to the upregulation of OPA1 (Muller-Rischart et al, 2013). Furthermore, Bernardini et al discovered direct ubiquitination of BAK by Parkin as a mechanism for suppressing apoptosis (Bernardini et al, 2019). However, an alternative perspective posits that Parkin possesses the ability to induce apoptosis. A proteomics study revealed that several BCL-2 family proteins, including MCL-1, BCL-2L13, and BAX, serve as substrates for Parkin (Sarraf et al, 2013). Subsequently, two independent studies demonstrated that Parkin ubiquitinates and degrades MCL-1 in a PINK1-mediated phosphorylation-dependent manner at serine 65 (pSer65) (Carroll et al, 2014; Zhang et al, 2014). Consistent with these findings, our research corroborated the role of Parkin in mediating the ubiquitination and proteasomal degradation of MCL-1 during mitochondrial depolarization-induced cell death. Furthermore, we have discovered that PTEN-L can counteract Parkin’s E3 ligase activity through dephosphorylation to stabilize MCL-1 and inhibit cell death. More importantly, our findings were further substantiated through the utilization of S63845, a specific inhibitor of MCL-1, thus reinforcing the pivotal role of MCL-1 as a downstream mediator regulated by both Parkin and PTEN-L in controlling cell death dynamics. Intriguingly though, we unexpectedly discovered that this form of cell death triggered by mitochondrial depolarization predominantly corresponds to pyroptosis based on compelling evidence: (1) clear observation of cellular swelling and formation of large bubbles protruding from the plasma membrane upon CCCP and O/A treatment, which are typical morphological features of pyroptosis rather than apoptosis (Rogers et al, 2017; Wang et al, 2017); (2) CCCP failed to elicit discernible apoptotic characteristics such as morphological alterations, DNA ladder formation, nuclear fragmentation, PARP cleavage as well as annexin V single-positive cells, while STS induced obvious apoptosis; (3) GSDME, the crucial executor of pyroptosis, was clearly cleaved and activated by caspase-3 in response to CCCP and O/A treatment; (4) importantly, CCCP significantly facilitated the localization of GSDME-N to the plasma membrane, which is the critical step to induce pyroptosis (Rogers et al, 2017; Wang et al, 2017); (5) moreover, knockdown of GSDME effectively mitigated CCCP-induced cell death. Our findings are consistent with previous studies demonstrating that GSDME can induce a switch from apoptosis to pyroptosis in HeLa cells (Wang et al, 2017). Therefore, the type of cell death triggered by CCCP may vary depending on the extent of mitochondrial damage and the expression levels of GSDME.

It is well-established that MCL-1, NOXA, and BFL-1 constitute a crucial regulatory axis in programmed cell death. In our experimental system, mitochondrial depolarization resulted in an elevation of NOXA expression and strengthened its interaction with MCL-1, regardless of PTEN-L expression. Silencing of NOXA reduced MCL-1 degradation and suppressed pyroptosis, indicating that NOXA is positioned upstream of PTEN-L in this pathway. Considering that NOXA can also trigger MOMP and that BFL-1 has been reported to sequester NOXA when MCL-1 is neutralized, we attempted to detect the interaction between NOXA and BFL-1 through reciprocal co-immunoprecipitation. However, we failed to obtain conclusive results, presumably because the antibodies were inappropriate for immunoprecipitation applications. We recognize this limitation and believe that future studies using optimized tools are required to clarify the potential role of BFL-1 in this process. Nevertheless, our data clearly identify NOXA as a significant upstream regulator that promotes MCL-1 degradation and facilitates pyroptosis following mitochondrial depolarization.

Previous studies have demonstrated that apoptosis and GSDME-mediated pyroptosis share significant overlap in upstream regulators (Xu et al, 2021; Zhou et al, 2018). Notably, caspase proteins such as caspase-9 and caspase-3 serve as prime examples in this context. Following cleavage and activation by caspase-9, caspase-3 initiates the cleavage of numerous proteins including PARP to induce apoptosis. Subsequently, compelling evidence has emerged indicating that activated caspase-3 can also cleave GSDME to mediate pyroptosis (Rogers et al, 2017; Wang et al, 2017). However, given that caspase-9 and caspase-3 have long been considered hallmarks of apoptosis (Yuan and Ofengeim, 2024), it is often feasible to infer that this form of cell death corresponds to apoptosis. Therefore, it is crucial to differentiate the exact type of cell death observed in relevant investigations. In addition to caspase proteins, the involvement of BCL-2 family proteins in pyroptosis has been demonstrated. For instance, during viral infection, MCL-1 deletion and BCL-xL inactivation promote pyroptosis by activating caspase-3 and GSDME (Orzalli et al, 2021). Studies have revealed that iron-activated ROS induces TOM20 oxidation and oligomerization, leading to the recruitment of BAX to mitochondria and subsequent activation of caspase-3 followed by GSDME cleavage, thereby triggering pyroptosis (Zhou et al, 2018). Although further research is required to fully elucidate the precise mechanisms underlying the differential regulation of apoptosis and pyroptosis, our findings reveal a critical regulatory mechanism that determines cell fate upon caspase-3 activation, specifically, whether cells undergo pyroptosis or apoptosis. This decision is governed by a two-signal integration: (1) a competence signal conferred by GSDME expression, and (2) an activation signal determined by the extent of mitochondrial damage. In cells lacking or expressing low levels of GSDME, caspase-3 activation leads unequivocally to apoptosis. However, in the presence of GSDME, the PINK1-Parkin axis amplifies mitochondrial damage, generating a robust caspase-3 signal that exceeds the threshold necessary for extensive GSDME cleavage and pore formation, thereby triggering pyroptosis. Conversely, weaker mitochondrial damage stress signals, or the presence of PTEN-L inhibiting Parkin activation, lead to a reduced caspase-3 signal, thereby promoting either apoptosis or cell survival. Thus, the PINK1-Parkin/PTEN-L module functions as a central regulatory switch that senses the extent of mitochondrial damage and directs caspase-3 activity toward distinct functional outcomes.

We have previously reported that PTEN-L exhibits dephosphorylation activity towards ubiquitin, thereby inhibiting Parkin-mediated mitophagy (Wang et al, 2018). The UBL domain of Parkin exhibits a structural resemblance to ubiquitin, with the conservation of Ser 65 observed in both proteins (Koyano et al, 2014). Here, we demonstrate that PTEN-L possesses the ability to dephosphorylate Parkin, thereby inhibiting Parkin-mediated pyroptosis and also Parkin-mediated mitophagy. First, we have demonstrated a direct interaction between PTEN-L and Parkin. Second, employing various techniques such as Phos-Tag assay, tandem mass spectrometry, immunoblotting, and immunostaining has provided compelling evidence that PTEN-L significantly reduces the levels of pSer65-Parkin in vivo following CCCP and O/A treatment. Third, our results indicate that PTEN-L does not influence the activities of PINK1, the upstream kinase of Parkin. Finally, separate in vitro dephosphorylation assays strongly support the notion that PTEN-L directly dephosphorylates Parkin through its protein phosphatase activity. Previous studies have highlighted that phosphorylation of Parkin’s UBL domain and pSer65-UB are both required for Parkin activation (Gladkova et al, 2018; Sauvé et al, 2018). Therefore, our findings suggest that PTEN-L sets two thresholds for Parkin activation. In contrast, canonical PTEN does not possess the same function as PTEN-L. This functional disparity likely mirrors their distinct sub-cellular distributions. The unique N-terminal extension of PTEN-L guides its localization to mitochondria, enabling it to access and dephosphorylate Parkin when mitochondrial depolarization occurs. Canonical PTEN, lacking this extension, is not effectively targeted to mitochondria and thus cannot regulate Parkin under such circumstances. Similarly, other negative regulators of Parkin also inhibit both Parkin-mediated mitophagy and Parkin-mediated cell death. For instance, MCL-1 antagonizes mitophagy by inhibiting the mitochondrial recruitment of Parkin (Hollville et al, 2014b), and it can also suppress Parkin-mediated apoptosis (Carroll et al, 2014). Additionally, USP30 blocks both mitophagy and apoptotic cell death by deubiquitinating Parkin’s substrates (Bingol et al, 2014; Liang et al, 2015). Interestingly, we have discovered that PTEN-L interacts with MCL-1 and stabilizes it. Given the well-discussed relationship between Parkin-mediated mitophagy and cell death (Bernardini et al, 2019; Carroll et al, 2014; Quarato et al, 2023), exploring the potential cross-talk among these negative regulators of Parkin in regulating mitophagy and cell death following mitochondrial damage would be intriguing. We also observed that PTEN-L still provided partial protection in GSDME knockdown or knockout cells when mitochondrial damage occurred. This residual protection can likely be ascribed to PTEN-L acting upstream of caspase activation. By dephosphorylating Parkin, PTEN-L lessens the magnitude of the initial mitochondrial insult, thus weakening the activation of the caspase cascade. Additionally, PTEN-L might also affect other signaling pathways that promote cell survival under these circumstances. We are dedicated to further investigating these aspects in our research.

In summary, our study identifies a previously unknown mitochondrial pathway that induces GSDME-dependent pyroptosis through the activation of the PINK1-Parkin axis, with PTEN-L serving as a critical regulatory inhibitor. The identification of the PTEN-L/Parkin/MCL-1 signaling axis not only deepens the molecular understanding of cell fate determination under mitochondrial stress but also presents promising therapeutic opportunities. Future studies should focus on elucidating the role of this pathway in mitochondrial damage-associated diseases, including cancer and neurodegenerative conditions, to support the development of novel therapeutic strategies.

## Methods

**Table:** Reagents and tools table

Reagent/resource	Reference or source	Identifier or catalog number
**Experimental models**		
HeLa cells	ATCC	CCL-2
HeLa stably expressing YFP-Parkin (HeLa YFP-Parkin)	Dr. Richard J. Youle	N/A
HeLa YFP-Parkin/PTEN-L OE	This study	N/A
HeLa YFP-Parkin/PTEN-L KO	This study	N/A
HeLa YFP-Parkin/PTEN OE	This study	N/A
HeLa YFP-Parkin/PTEN and PTEN-L DKO	This study	N/A
HeLa YFP-Parkin/PTEN only	This study	N/A
HeLa YFP-Parkin/GSDME KO	This study	N/A
HeLa YFP-Parkin/PTEN-L OE/GSDME KO	This study	N/A
HeLa YFP-Parkin/ BAX and BAK DKD	This study	N/A
HeLa YFP-Parkin/PTEN-L OE/BAX and BAK DKD	This study	N/A
HeLa YFP-Parkin/MCL-1 KD	This study	N/A
HeLa YFP-Parkin/PTEN-L OE/MCL-1 KD	This study	N/A
HeLa YFP-Parkin/NOXA KD	This study	N/A
HeLa YFP-Parkin/PTEN-L OE/NOXA KD	This study	N/A
HeLa YFP-Parkin/mito-keima	This study	N/A
HeLa YFP-Parkin/mito-keima/PTEN-L OE	This study	N/A
HeLa stably expressing HA-Parkin (HeLa HA-Parkin)	This study	N/A
SH-SY5Y cells	ATCC	CRL-2266
SH-SY5Y stably expressing YFP-Parkin (SH-SY5Y YFP-Parkin)	This study	N/A
SH-SY5Y/PTEN-L OE	This study	N/A
SH-SY5Y/ PTEN-L KO	This study	N/A
SH-SY5Y/GSDME KD	This study	N/A
HEK293T cells	ATCC	CL-0005
**Recombinant DNA**		
pCMV-Tag2B-PTEN-L	This study	N/A
pCMV-Tag2B-PTEN-L^C297S^	This study	N/A
pCMV-Tag2B-PTEN-L^G302R^	This study	N/A
pCMV-Tag2B-PTEN	This study	N/A
pCMV-Tag2B-Parkin	This study	N/A
pmcherry-PTEN-L	This study	N/A
pET30a-WT PTEN-L	This study	N/A
pET30a-PTEN-L^C297S^	This study	N/A
pET30a-PTEN-L^G302R^	This study	N/A
pCDH-HA-GSDME	This study	N/A
pCDH-HA-GSDME ^D267A/D270A^	This study	N/A
lentiCRISPR v2-sgPTEN-L	This study	N/A
lentiCRISPR v2-sgGSDME	This study	N/A
lentiCRISPR v2-sgPTEN and PTEN-L	This study	N/A
pPLKO.1-shBAX and BAK	This study	N/A
pPLKO.1-shGSDME	This study	N/A
pPLKO.1-shMCL-1	This study	N/A
pPLKO.1-shNOXA	This study	N/A
pEBG vector	Dr. Jongkyeong Chung	N/A
pEBG-Parkin WT	Dr. Jongkyeong Chung	N/A
pEBG-Parkin S65D	Dr. Jongkyeong Chung	N/A
eYFP-C1-Parkin	Dr. Richard J. Youle	N/A
pCDH-YFP-Parkin-puro	Dr. Han-Ming Shen	N/A
pEGFP-C1-MCL-1	This study	N/A
pRK5-HA-Ub	Addgene	17608
pEGFP-N1-PINK1	Dr. Kah-Leong Lim	N/A
**Antibodies**		
Rabbit anti-Caspase-3	Cell Signalling Technology	9662
Rabbit anti-Cleaved Caspase-3	Cell Signalling Technology	9661
Rabbit anti-Caspase-9	Cell Signalling Technology	9502
Rabbit anti-PARP	Cell Signalling Technology	9542
Rabbit anti-PINK1	Cell Signalling Technology	6946
Rabbit anti-GFP	Cell Signalling Technology	2956
Mouse anti-Parkin	Cell Signalling Technology	4211
Rabbit anti-PTEN	Cell Signalling Technology	138G6
Rabbit anti-PTEN	Cell Signalling Technology	9559
Rabbit anti-BCl-2	Cell Signalling Technology	2876
Rabbit anti-BAK	Cell Signalling Technology	12105
Rabbit anti-GSDMD	Cell Signalling Technology	39754
Rabbit anti-GAPDH	Cell Signalling Technology	2118
Mouse anti-Actin	Sigma-Aldrich	A5441
Mouse anti-Tubulin	Sigma-Aldrich	T6199
Rabbit anti-GSDMD	Proteintech	20770-1-AP
Mouse anti-Flag	ABclonal	AE005
Rabbit anti-LDHA	ABclonal	A0861
Mouse anti-HA	ABclonal	AE008
Rabbit anti-PMAIP1 (NOXA)	ABclonal	A9801
Mouse anti-α1 sodium potassium ATPase	Abcam	ab215191
Rabbit anti-pSer65-Parkin	Abcam	ab7671
Rabbit anti-BFL-1 (BCL-2A1)	Abcam	ab315376
Rabbit anti-GSDME	Abcam	ab287160
Mouse anti-Tim23	BD Biosciences	611223
Mouse anti-BAX	BD Biosciences	610983
Mouse anti-Cytochrome c	BD Biosciences	556433
Mouse anti-Cytochrome c	Promab	P20788
Mouse anti-BAX(6A7)	Novus Biologicals	NBP1-28566
Rabbit anti-MCL-1	Santa Cruz Biotechnology	S-19
Mouse anti-MCL-1	Santa Cruz Biotechnology	sc-819
Mouse anti-Tom20	Santa Cruz Biotechnology	sc-17764
Mouse anti-Alexa Fluor® 647 Cytochrome c	BioLegend	612310
Mouse anti-IgG1 kappa Isotype Control	Invitrogen	51-4714-81
Mouse anti-Alexa Fluor™ 647	Invitrogen	A-21235
Rabbit anti-Alexa Fluor™ 647	Invitrogen	A-21245
Mouse anti-Alexa Fluor™ 488	Invitrogen	A-32723
Rabbit anti-Alexa Fluor™ 488	Invitrogen	A-32731
Mouse anti-Alexa Fluor™ 555	Invitrogen	A-32727
Rabbit anti-Alexa Fluor™ 555	Invitrogen	A-32732
Mouse anti-IgG, light chain specific	Jackson ImmunoResearch	115-035-174
Rabbit anti-IgG, light chain specific	Jackson ImmunoResearch	211-032-171
Mouse anti-IgG-HRP	Absin	abs20039
Rabbit anti-IgG-HRP	Absin	abs20040
**Chemicals, enzymes and other reagents**		
Q-VD-OPh	MedChemExpress	HY-12305
Staurosporine	MedChemExpress	HY-15141
TNF-α	MedChemExpress	HY-P7058
Cisplatin	MedChemExpress	HY-17394
Doxorubicin	MedChemExpress	HY-15142
Ferrostatin-1	MedChemExpress	HY-100579
Necrostatin-1	MedChemExpress	HY-15760
Necrostatin 2 racemate	MedChemExpress	HY-14622
Cycloheximide	MedChemExpress	HY-12320
ABT-737	MedChemExpress	HY-50907
TAK-243	MedChemExpress	HY-100487
Oligomycin	Sigma-Aldrich	75351
Antimycin	Sigma-Aldrich	A8674
Carbonyl cyanide 3-chlorophenylhydrazone	Sigma-Aldrich	C2759
Z-VAD-FMK	Targetmol	T6013
S63845	Targetmol	T5346
MG132	Targetmol	T001-10011605
DMSO	Solarbio	D8371
Bafilomycin A1	Selleck	S1413
Phosphatase inhibitor	TargetMol	C0004
Proteinase inhibitor cocktail	TargetMol	C0001
PVDF membrane	Merck	TPVH00010[334485]
Enhanced chemiluminescence	4A BIOTECH	4AW011-200
Fetal Bovine Serum	Excell	FSP500
Opti-MEM	Gibco	31985-070
Medium Dulbecco’s Modified Eagle Medium	Procell	PM150210
TAE buffer	Biosharp	BL533A
TS-GelRed nucleic acid gel stain Ver.2	TSINGKE	TSJ003
Puromycin Dihydrochloride	Yeasen	60210ES25
Polybrene	Beyotime	C0351
GZ-C07 Lipo8000	Beyotime	C0533
AgeI-HF®	NEW ENGLAND Biolabs	R3552S
EcoRI-HF®	NEW ENGLAND Biolabs	R3101S
BamHI-HF®	NEW ENGLAND Biolabs	R3136S
BsmBI-v2	NEW ENGLAND Biolabs	R0739S
Propidium iodide	Sangon Biotech	A601112
SYTOX Green staining	Thermo Fisher	S7020
**Oligonucleotides and other sequence-based reagents**		
si GSDME#1	*Homo sapiens*	GCGGUCCUAUUUGAUGAUGAA
si GSDME#2	*Homo sapiens*	GAUGAUGGAGUAUCUGAUCUU
si BAX#1	*Homo sapiens*	GGGACGAACUGGACAGUAA
si BAX#2	*Homo sapiens*	AAGGUGCCGGAACUGAUCAGA
si GSDMD#1	*Homo sapiens*	GUGUGUCAACCUGUCUAUCAA
si GSDMD#2	*Homo sapiens*	CAGCACCUCAAUGAAUGUGUA
si BAK#1	*Homo sapiens*	CCCAUUCACUACAGGUGAA
si BAK#2	*Homo sapiens*	GUACGAAGAUUCUUCAAAU
BAX KD	*Homo sapiens*	CCGGGACGAACTGGACAGTAACATGCTCGAGCATGTTACTGTCCAGTTCGTCTTTTTG
BAK KD	*Homo sapiens*	CCGGTGGTACGAAGATTCTTCAAATCTCGAGATTTGAAGAATCTTCGTACCATTTTTG
GSDME KD	*Homo sapiens*	CCGGGATGATGGAGTATCTGATCTTCTCGAGAAGATCAGATACTCCATCATCTTTTTG
MCL-1 KD	*Homo sapiens*	CCGGGCCTAGTTTATCACCAATAATCTCGAGATTATTGGTGATAAACTAGGCTTTTTG
NOXA KD	*Homo sapiens*	CCGGGCATTGTTGTTGTTGCTGTTTCTCGAGAAACAGCAACAACAACAATGCTTTTTG
GSDME KO	*Homo sapiens*	CACCGTAAGTTACAGCTTCTAAGTC
PTEN-L KO	*Homo sapiens*	CACCGCGGCGGCACATCCAGGGACC
PTEN/PTEN-L DKO	*Homo sapiens*	CACCGTCATCTGGATTATAGACCAG
PTEN-L only	*Homo sapiens*	CCAGACATAACAGCCATCATCAA
**Real-time quantitative polymerase chain reaction oligonucleotides**		
Homo GSDME	TGATCTCTGAGCACATGCAG	TCCTTGGTGACATTCCCATC
Homo MCL-1	CCAAGAAAGCTGCATCGAACCAT	CAGCACATTCCTGATGCCACCT
Homo BAX	TGACGGCAACTTCAACTGGG	AGCACTCCCGCCACAAAGA
Homo GAPDH	GACCCCTTCATTGACCTCAAC	CTTCTCCATGGTGGTGAAGA
**Software**		
GraphPad Prism	https://www.graphpad.com/	Version 9.5.1
ImageJ	https://imagej.net/ij/	Version 1.8.0
FlowJo	Tree Star Software	Version 10.10.0
Zen light	Zeiss	Version 3.8
**Other**		
MDX10 microscope	Mshot	N/A
LSM 980	Zeiss	N/A
EVOS M5000	Thermo Fisher	N/A
Synergy H1	Bio Tek	N/A
ChemiDoc™ Imaging System	BIO-RAD	N/A

### Plasmid constructs

DNA sequences encoding WT PTEN-L, its mutants PTEN-L^C297S^ and PTEN-L^G302R^, PTEN or Parkin were inserted to pCMV-Tag2B for eukaryotic expression and pET30a with an N-terminal 6×His tag for prokaryotic expression. pmCherry-C1 plasmids were used to construct PTEN-L or Parkin overexpressing plasmids. pEGFP-C1 plasmids were engineered to containing DNA sequences encoding MCL-1 or PTEN-L. pCDH-HA-blasticidin plasmids were engineered to containing DNA sequences encoding WT GSDME, GSDME^D267A/D270A^ or Parkin. HA-Ub, pHAGE-mt-mKeima and eYFP-C1-Parkin plasmids were kind gifts from Dr. Richard J. Youle (National Institute of Neurological Disorders and Stroke, USA). PINK1 C-GFP plasmids and Flag-Parkin were kind gifts from Dr. Kah-Leong Lim (National Neuroscience Institute, Singapore). 3×Flag-GSDME were kind gifts from Dr. Yigang Wang (College of Life Science, Zhejiang Sci-Tech University). pCDH-YFP-Parkin-puro were kind gifts from Dr. Han-Ming Shen (Faculty of Health Sciences, University of Macau). Site-directed mutagenesis was performed by using the QuickChange Site-Directed Mutagenesis Kit following the manufacturer’s instructions. All plasmid constructs were confirmed by restriction digestion or DNA sequencing. Expression of all constructs was confirmed by immunoblotting.

### Cell lines, cell culture, and transfections

Cells were maintained in Dulbecco’s modified Eagle’s medium (Procell, PM150210) with 10% fetal bovine serum (ExCell Bio, FSP500) and 1% penicillin–streptomycin (Solarbio, P1400) in a 5% CO_2_ incubator at 37 °C. Transfections with plasmids were performed with Lipofectamine 3000 (Thermo Fisher Scientific, L3000015) according to the manufacturer’s instructions when cells were grown to 70% confluency.

HeLa cells stably expressing YFP-Parkin (HeLa YFP-Parkin cells) were kind gifts from Dr. Richard J. Youle. The following cell lines were generated based on HeLa YFP-Parkin cells: HeLa YFP-Parkin Vector/PTEN-L or PTEN cells and HeLa YFP-Parkin WT/PTEN-L KO cells, as previously described (Wang et al, 2018).

To generate new stable cell lines, including stable gene knockdown (BAX, BAK, GSDME, MCL-1 and NOXA), knockout (PTEN-L, PTEN and GSDME) and over-expression (YFP-Parkin), lentiviral particles were produced in HEK293T cells co-transfected with pLKO.1-puro-shRNA, LentiCRISPR v2-sgRNA or over-expression constructs along with psPAX2 and pMD2.G using PEI. Viral supernatants were collected, filtered, and then used to infect target cells in the presence of polybrene (2 μg/mL). Transduced cells were selected with puromycin (1 μg/mL). The targeting sequences of shRNA and sgRNA are listed in the Reagents and Tools Table.

To generate HeLa YFP-Parkin cells with a homozygous PTEN start codon mutation (ATG > ATA), we utilized CRISPR/Cas9-mediated homology-directed repair. The cells were co-transfected with validated guide RNAs and a single-stranded oligodeoxynucleotide donor that carried the desired mutation along with a silent mutation to prevent re-cleavage. After electroporation, the cells underwent transient puromycin selection at a concentration of 2 μg/mL. The pooled cell population was evaluated by next-generation sequencing, which showed an approximately 9% HDR efficiency, and then single-cell cloning was performed through limiting dilution. Individual clones were expanded and genotyped via Sanger sequencing to identify the homozygous mutants. This cell line only expresses PTEN-L rather than canonical PTEN, which was named as PTEN-L only cells.

### Rescue experiments

For rescue assays, GSDME knockout cells were transfected with plasmids encoding WT or mutant GSDME cDNA using Lipofectamine 8000. To prevent re-cleavage by the CRISPR/Cas9 system, silent mutations were introduced into the single-guide RNA (sgRNA) target sequence (including the protospacer adjacent motif, PAM) of the WT and D267/270A construct. At 48 h post-transfection, cells were harvested for downstream analyses. Empty vector-transfected knockout cells served as negative controls.

### Reverse transfection for small interfering RNA (siRNA)

Transient transfection was performed with Lipofectamine RNAi MAX (Thermo Fisher Scientific, 13778150) according to the manufacturer’s instructions. The siRNAs were synthesized by Sangon Biotech and their sequences are listed in the Reagents and Tools Table.

### Real-time quantitative polymerase chain reaction

RNA was extracted by TransZol Up (TransGen Biotech, ET111-01-V2). The cDNA synthesis and Real-time fluorescent quantitative PCR was performed using the PerfectStart Uni RT&qPCR kit (TransGen Biotech, AUQ-01) according to the manufacturer’s instructions. The primer sequences are listed in the Reagents and Tools Table.

### Live cell imaging

Cells were seeded in six-well plates at 40–60% confluence and treated as indicated. For live-cell bright-field imaging, an Mshot MDX10 camera (Mshot, China) mounted on an inverted microscope with a 20× objective was used. The 1-inch CMOS sensor (2.4 µm/pixel) captured images at default resolution under bright-field illumination with automatic exposure, automatic gain, and global white balance (MShot Image Analysis System). Images were saved as TIFF, and scale bars were added using the same software. Imaging was carried out at room temperature, with at least 3 random fields per coverslip and three independent experiments. For each experimental condition, at least 3 random fields of view were selected and imaged. Cell numbers were counted and averaged per field. At least 200 cells were counted per condition across 3 independent experiments.

### Immunofluorescence microscopy

For immunostaining, the cells were washed twice with phosphate-buffered saline (PBS) and fixed with 4% paraformaldehyde in PBS for 15 min at room temperature. Then cells were permeabilized with 0.25% Triton X-100 for 15 min and blocked in 10% BSA in PBS for 30 min at 37 °C before being stained with the indicated antibodies. Primary antibodies were used at 1:200 overnight at 4 °C, followed by secondary antibodies at 1:1000 for 1 h at 37 °C. After washed with PBS, coverslips were mounted with Antifade Mounting Medium (Beyotime, P0131). Immunofluorescence images were acquired on a Zeiss LSM 980 confocal microscope that was equipped with an Airyscan 2 detector and a 40×/1.3 Oil DIC objective. The Airyscan super-resolution mode with bidirectional scanning and two-frame averaging was used. The acquisition was performed with ZEN 3.8 (Lite) software; the raw data were saved in CZI format, and Airyscan-processed images were exported as TIFF. The laser power, gain, and pinhole were maintained constant across all conditions to allow quantitative comparison. At least four random fields per coverslip were imaged per condition, with three independent biological replicates. The fluorescence intensity was quantified using ImageJ 1.53k (NIH, USA). For each experimental condition, 3 random fields of view were selected for imaging. Fluorescence intensity was quantified using ImageJ software, and the mean fluorescence intensity per cell was calculated. At least 200 cells were analyzed per condition across 3 independent experiments.

### Electron microscopy

For electron microscopy, HeLa YFP-Parkin Vector/PTEN-L cells were fixed in 2.5% glutaraldehyde in 0.1 M PBS overnight at 4 °C. After rinsing with PBS, cells were post-fixed with 1% osmium tetroxide, dehydrated through a graded ethanol series, and then processed for either transmission or scanning electron microscopy. For transmission electron microscopy (TEM), samples were infiltrated and embedded in epoxy resin. Ultrathin sections were cut, stained with uranyl acetate and lead citrate, and examined under a transmission electron microscope. For scanning electron microscopy (SEM), samples were critical-point dried, mounted on stubs, sputter-coated with gold, and visualized under a scanning electron microscope.

### Colony formation assay

The cells were seeded in six-well plates at consistent cell densities overnight. Subsequently, the cells were exposed to CCCP (20 μM) for a duration of 12 h. Following this treatment, the cells were allowed to proliferate for a period of 5 days until visible colonies formed, after which they were fixed and stained using methanol and crystal violet.

### Immunoblotting

After following the prescribed treatments, the collected cells were lysed with Laemmli SDS buffer (62.5 mM Tris-HCl, pH 6.8, 25% glycerol, 2% SDS, supplemented with a phosphatase inhibitor [TargetMol, C0004 - 1 mL] and a proteinase inhibitor cocktail [TargetMol, C0001 - 1 mL]). An equal quantity of protein was separated via SDS-PAGE on 8%, 10%, or 12% polyacrylamide gels, which were selected according to the molecular weight of the target protein. Subsequently, the proteins were transferred onto a PVDF membrane (Merck, TPVH00010 [334485]). After blocking the membrane with 5% non-fat milk for 1 h, it was probed with primary antibodies overnight at 4 °C. Then, it was incubated with HRP-conjugated secondary antibodies for 1 h at room temperature. Proteins were visualized through enhanced chemiluminescence (4A BIOTECH, 4AW011-200). Western blot bands were quantified by densitometry using ImageJ software (National Institutes of Health, Bethesda, MD, USA). The grayscale value of each band was measured, and the relative protein expression level was calculated by normalizing the target protein signal to the loading control (β-actin or Tubulin). The value of the control group was set to 1. All quantifications were derived from three independent biological replicates.

### DNA agarose gel electrophoresis

DNA agarose gel electrophoresis was carried out for 30 min at 100 V with 1% agarose gel in 1× TAE buffer (Biosharp, BL533A) containing TS-GelRed nucleic acid gel stain Ver.2 (TSINGKE, China) at a final dilution of 1:10,000, then visualized under UV light.

### Immunoprecipitation and Immunoblotting analysis

For immunoprecipitation assays, HEK293T cells were transfected with the indicated plasmids for 24 h, then cells were lysed with lysis buffer (20 mM Tris, pH 8.0, 150 mM NaCl, 0.5% NP-40, 1 mM EDTA, pH 8.0 and 10 mM NEM and protease inhibitor cocktail). Lysates were pre-cleared using protein A/G agarose and subsequently subjected to overnight immunoprecipitation with the indicated antibodies at 4 °C. Following an additional 2 h incubation with protein A/G agarose at 4 °C, the immunoprecipitates were washed four times with lysis buffer and subsequently analyzed by western blotting. For the detection of MCL-1 ubiquitination, cells were pretreated with MG132 (10 μM) for 1 h prior to CCCP treatment. Grayscale values of corresponding bands were measured using ImageJ 1.53k (National Institutes of Health) for quantification of GSDME, MCL-1, pSer65-Parkin, and other proteins. The fold change was calculated by comparing the treated groups with control samples.

### Phos-tag gels

To analyze PINK1 phosphorylation, lysates were prepared in sample buffer without EDTA and subjected to electrophoresis on 7.5% Tris-glycine gels containing 50 µM Phos-tag (FUJIFILM Wako Pure Chemical Corporation) and 100 µM MnCl_2_ according to the manufacturer’s instructions. Simultaneously, SDS-PAGE gels were run as control.

### Recombinant protein purification and Coomassie blue staining

*E. coli* BL21 (DE3) cells (TransGen Biotech, CD601-02) were transformed with the recombinant plasmid (pET30a-WT PTEN-L, pET30a-PTEN-L^C297S^ and pET30a-PTEN-L^G302R^ plasmids). A single colony was inoculated into LB medium containing kanamycin; cultures were incubated in 37 °C at 200 rpm. Once cell density reached to OD = 0.6–0.8 at 600 nm, IPTG (200 mM; Sangon Biotech, A100487-0005) was introduced for induction. The recombinant proteins were affinity-purified by Ni-Sepharose beads (Sangon Biotech, C600033-0010). 1 μg of purified recombinant proteins were subjected to SDS–PAGE followed by Coomassie blue (Beyotime, P0003FT) staining.

### In vitro dephosphorylation assay

The PTEN-L proteins used in these assays were purified from two distinct sources: one from HEK293T cells and the other from *E. coli*. HEK293T cells were transiently transfected with empty vector or vectors encoding Flag-WT PTEN-L, Flag-PTEN-L^C297S^ and Flag-PTEN-L^G302R^. After 24 h, cells were lysed with 0.5% NP-40 immunoprecipitation buffer at 4 °C for 30 min. The lysed protein samples were then centrifuged to obtain the supernatant. Flag beads, which had been washed once with NP-40 buffer, were added to the samples and incubated at 4 °C for 2 h. Subsequently, the samples were washed three times with 1×PBS followed by two washes with phosphatase reaction buffer containing 20 mM HEPES (pH 7.2), 1 mM DTT, 1 mM MgCl_2_, and 0.1 mg/mL BSA along with protease inhibitors. This was followed by incubation of the samples with phosphatase reaction buffer and commercialized pSer65-Parkin protein (UBIQUIGENT, 69-1100-BUL) at a temperature of 37 °C for a duration of 2 h. Similarly, an equal amount of 6×His-WT PTEN-L, 6×His-PTEN-L^C297S^, 6×His-PTEN-L^G302R^, and control proteins from *E. coli* were added to the phosphatase reaction buffer containing pSer65-Parkin, followed by incubation at 37 °C for 2 h.

### Cell death measurement (PI uptake, LDH release, SYTOX green and Annexin V/PI staining)

The cells were harvested and resuspended in PBS containing 3 μM propidium iodide (Sangon Biotech, A601112). The PI-positive cells were quantified using a BD FACSCelesta flow cytometer, at least 10,000 cells were counted per sample. LDH release in cell culture supernatants was analyzed with the LDH Cytotoxicity Assay Kit (Beyotime, C0016) following the manufacturer’s protocol. For SYTOX green staining, the cells were seeded into the µ-Slide 8 well comes with a thin ibidi polymer coverslip bottom and incubated overnight. To detect dead cells, SYTOX Green (Thermo Fisher, S7020) was added to the medium together with the drugs at a final concentration of 167 nM. After treatment, the cells were then observed under a fluorescence microscope and detection was completed within 1 h. At least 200 cells were analyzed per condition across 3 independent experiments. The SYTOX Green signal (Ex/Em = 488/500–525 nm) was effectively separated from YFP (Ex/Em = 514/525–565 nm) to avoid spectral crosstalk. Annexin V/PI staining was performed using the Annexin V-FITC Apoptosis Detection Kit (Beyotime, C1062M), according to the manufacturer’s instructions. Although both PI and SYTOX green are membrane-impermeant nucleic acid stains that detect plasma membrane permeabilization, in this study, PI staining was used for endpoint quantification by flow cytometry. In contrast, SYTOX Green staining was performed in conjunction with confocal microscopy to simultaneously visualize cell morphology and quantify dead cells within the same field of view.

### Mt-Keima mitophagy assay

HeLa YFP-Parkin Vector/PTEN-L cells stably expressing mt-Keima generated using lentiviral expression system were treated with CCCP 20 μM and Z-VAD 20 μM for 24 h. Measurements of lysosomal mt-Keima were made on the BD FACSCelesta flow cytometer using dual-excitation ratiometric pH measurements at 488 nm (pH 7) and 561 nm (pH 4) lasers, respectively, following the described protocols (Vargas et al, 2019).

### Cytochrome c release assay measured by flow cytometry

To monitor cytochrome c release, cells were pretreated with Q-VD-OPh (40 μM, 1 h) and then treated with CCCP (20 μM, 6 h). Subsequently, the cells were incubated on ice with 25 μg/ml digitonin in PBS for 10 min to permeabilize the membrane. After that, they were resuspended in cold Fixation buffer (YEASEN, 40402ES50) and incubated at room temperature for 10 min. Following this step, the cells were resuspended in Permeabilization buffer (YEASEN, 40403ES64) and incubated at room temperature for another 15 min. For staining purposes, the cells were kept on ice and exposed to Alexa Fluor® 647 anti-Cytochrome c Antibody or Mouse IgG1k Isotype Ctrl in permeabilization buffer for a duration of 30 min. Finally, after washing and resuspending them in PBS solution, flow cytometric analysis was performed on the BD FACSCelesta flow cytometer, at least 10,000 cells were counted per sample. Analysis was used by FlowJo software (v10; Tree Star).

### Caspase-3/7 activity assay

Caspase-3/7 activity was measured using the Caspase-Glo® 3/7 Assay (Promega, G8092) according to the manufacturer’s instructions. Briefly, 1 × 10^4^ cells were seeded in 96-well white opaque plates and treated as indicated. At the endpoint, an equal volume of Caspase-Glo® 3/7 Reagent (prepared by transferring the buffer to the substrate bottle and mixing until fully dissolved) was directly added to each well containing the cell culture medium. The plates were gently mixed on an orbital shaker at 300 rpm for 30 s and incubated at room temperature for 30 min to 3 h. Luminescence was recorded using a microplate luminometer.

### Mitochondrial ROS production assay measured by flow cytometry

Cells were cultured in a 6-well plate and treated as indicated. To detect mitochondrial ROS, cells were labeled with MitoSOX™ (Invitrogen, M36008). After incubation, cells were washing, detaching and resuspended in PBS. Fluorescence was detected using a FACSCalibur flow cytometry (BD Biosciences) with PE channel, at least 10,000 cells were counted per sample. Statistical analysis was used by FlowJo software (v10; Tree Star).

### Plasma membrane enrichment

The cell fractionation procedure was conducted using a PM extraction kit (Abcam, Ab65400). Briefly, cells were gently detached and collected, followed by homogenization in a douncer with homogenization buffer. Subsequently, sequential centrifugation steps were performed according to the manufacturer’s instructions to separate cytosolic and total membrane proteins.

### Subcellular fraction

2 × 10^6^ cells were harvested via trypsinization, washed twice with ice-cold PBS, and resuspended in 400 μL of a buffer containing Digitonin (150 mM NaCl, 50 mM HEPES at pH 7.4, 25 μg/mL Digitonin, along with protease and phos-phatase inhibitors) and gently rotated at 4 ^◦^C for 10 min. Subsequently, the samples underwent centrifugation at 2000 × *g* for 10 min at 4 ^◦^C to separate the cytosolic fraction (supernatant). The obtained supernatants were carefully decanted into clean tubes and centrifuged three times at 20,000 × *g* for 20 min at 4 ^◦^C to ensure the removal of pellet and subsequently transferred into fresh tubes. To isolate the crude mitochondrial fraction, the remaining pellet was resuspended in ice-cold PBS to remove any residual Digitonin buffer and centrifuged again at 2000 × *g* for 5 min at 4 ^◦^C. The pellet was then lysed with 400 μL of NP-40 buffer (150 mM NaCl, 50 mM HEPES at pH 7.4, 1% NP-40, supplemented with protease and phosphatase inhibitors) and incubated on ice for 30 min. Finally, the samples were centrifuged at 7000 × *g* for 10 min at 4 ^◦^C to obtain the crude mitochondrial fraction.

### Proteomics analysis

For proteomics analysis, cells pellets were harvested and immediately frozen using liquid nitrogen, and transported with dry ice to Hangzhou Cosmos Wisdom Biotech Co., Ltd (Hangzhou, China) for sample preparation and LC-MS/MS analysis. LC-MS/MS analysis was performed on an Orbitrap Astral mass spectrometer coupled to a Thermo Scientific Vanquish Neo UHPLC system. A 5.5 cm High Throughput μPAC™ Neo HPLC Column was used for peptide separation with a 8 min or 14 min gradient of mobile phase B from 4% to 35% at 2.5 μL/min. The Orbitrap Astral was operated at full-MS resolution at 240,000 with a scan range of 380–980 *m*/*z* and normalized AGC target were set to 500% with a Maximum IT of 5 ms. Data independent acquisition (DIA) was performed with 300 scan windows at 2 Th isolation window and Normalized HCD Collision Energy was set at 25%. Raw files were analyzed in DIA-NN 1.8.1 using an in silico DIA-NN predicted spectral library.

### Functional enrichment analysis

Differentially expressed proteins (DEPs) identified through quantitative proteomic analysis were subjected to Gene Ontology (GO) enrichment analysis and Gene Set Enrichment Analysis (GSEA). The GO enrichment analysis was conducted using the Gene Ontology annotation database to identify significantly enriched Biological Process (BP) and Cellular Component (CC) terms. Statistical significance was evaluated via the hypergeometric test, and the resultant *P* values were adjusted for multiple comparisons using the Benjamini–Hochberg (BH) method. GO terms with an adjusted *P* value (false discovery rate, FDR) less than 0.05 were regarded as significantly enriched. GSEA was executed using the GSEA software (version 4.3.2, Broad Institute, Cambridge, MA, USA) and the Molecular Signatures Database (MSigDB v2023.2.Hs). All quantified proteins were ranked according to their log2 fold change between PTEN-L overexpressing cells and vector controls. Enrichment scores (ES), normalized enrichment scores (NES), nominal *P* values, and FDR values were computed using 1000 phenotype permutations. Gene sets with a nominal *P* value less than 0.05 and FDR less than 0.25 were considered to be significantly enriched. All enrichment analyses were carried out using the proteomics data generated in this study (PTEN - L overexpression versus vector control).

### Statistical analysis and reproducibility

Please refer to the figure legends for the description of sample sizes and statistical tests performed. Statistical analyses were performed using GraphPad Prism 9.5.1. Between-group differences were assessed by a two-tailed Student’s *t* test (two-sample comparisons) or one-way ANOVA with Tukey’s post hoc test (multi-group comparisons). For experiments involving two independent variables, two-way ANOVA with Sidak’s multiple comparisons test was applied. Data are expressed as mean SD from ≥3 independent experiments. Statistical significance was defined as *P*  <  0.05.

### Graphics

Figure 2L, Fig. 7, and the synopsis graphics were created with BioRender.com.

## Supplementary information

Peer Review File

Appendix

Source data Fig. 1

Source data Fig. 2

Source data Fig. 3

Source data Fig. 4

Source data Fig. 5

Source data Fig. 6

## Data Availability

The mass spectrometry proteomics data have been depositedin the ProteomeXchange Consortium via the iProX partnerrepository under ProteomeXchange dataset identifier PXD081568 (iProX: IPX0018525000) and PXD081677 (iProX: IPX0018613000), and are available at https://www.iprox.cn/page/project.html?id=IPX0018525000 and https://www.iprox.cn/page/project.html?id=IPX0018613000, respectively. The source data of this paper are collected in the following database record: biostudies:S-SCDT-10_1038-S44319-026-00957-4.
