# Supplementary material for: PTEN-L inhibits mitophagy and GSDME-dependent pyroptosis via Parkin dephosphorylation
Source: EMBO Rep. 2026 Sep 28;27(21):6166–90. doi: 10.1038/s44319-026-00957-4 (PMC13743760; doi:10.1038/s44319-026-00957-4)
Supplement: Supplementary file 1 [file 44319_2026_957_MOESM1_ESM.pdf]

EMBO reports

PTEN-L inhibits mitophagy and GSDME-dependent pyroptosis via Parkin dephosphorylation

Liming Wang, Chenghao Yan, Cainian Huang, Xu Liu, Yifan Liu, Yancheng Tang, Zhixuan Zhu, Jiaqi Qi, Hao Guo, Ying Li, Yuyuan Zhu, Lijie Wang, Yufei Luo, Linlin Xing, Haiji Wang, Faxiang Li, Bo Sun, Sulai Liu, Guang Lu, Haodong Xu, Bo Zhou, and Han-Ming Shen

Corresponding author(s): Liming Wang (wangliming@hnu.edu.cn)

Review Timeline:

Submission Date:	8th Dec 25
Editorial Decision:	9th Feb 26
Revision Received:	12th Jul 26
Editorial Decision:	4th Sep 26
Revision Received:	5th Sep 26
Accepted:	16th Sep 26

Editor: Martina Rembold

Transaction Report:

(Note: With the exception of the correction of typographical or spelling errors that could be a source of ambiguity, letters and reports are not edited. Depending on transfer agreements, referee reports obtained elsewhere may or may not be included in this compilation. Referee reports are anonymous unless the Referee chooses to sign their reports.)

1st Editorial Decision 9th Feb 2026

Dear Prof. Wang

Thank you for the submission of your manuscript to EMBO reports. I apologize for the delay in handling it, but we have now received the full set of referee reports that are all pasted below.

As you will see, the referees acknowledge that the findings are potentially interesting but they also raise numerous concerns regarding the type of cell death, the insufficient analysis of endogenous proteins, missing controls and informatioin of experimental conditions, potentially different kinetics and the specific role of MCL-1. I think that all concerns are pertinent and must be addressed.

While it is clear that a lot of work will be required to substantiate the findings, I would thus like to give you the chance to revise your manuscript with the understanding that the referee concerns must be fully addressed and their suggestions taken on board. Please address all referee concerns in a complete point-by-point response. Acceptance of the manuscript will depend on a positive outcome of a second round of review. It is EMBO reports policy to allow a single round of major revision only and acceptance or rejection of the manuscript will therefore depend on the completeness of your responses included in the next, final version of the manuscript.

We realize that it is difficult to revise to a specific deadline. In the interest of protecting the conceptual advance provided by the work, we recommend a revision within 3 months (12th May 2026). Please discuss the revision progress ahead of this time with the editor if you require more time to complete the revisions.

You can either publish the study as a short report or as a full article. For short reports, the revised manuscript should not exceed 27,000 characters (including spaces but excluding materials & methods and references) and 5 main plus 5 expanded view figures. The results and discussion sections must further be combined, which will help to shorten the manuscript text by eliminating some redundancy that is inevitable when discussing the same experiments twice. For a normal article there are no length limitations, but it should have more than 5 main figures and the results and discussion sections must be separate. In both cases, the entire materials and methods must be included in the main manuscript file.

IMPORTANT NOTE: we perform an initial quality control of all revised manuscripts before re-review. Your manuscript will FAIL this control and the handling will be DELAYED if the following APPLIES:
1) A data availability section providing access to data deposited in public databases is missing. If you have not deposited any data, please add a sentence to the data availability section that explains that.
2) Your manuscript contains statistics and error bars based on n=2. Please use scatter blots in these cases. No statistics should be calculated if n=2.

When submitting your revised manuscript, please review the submission guidelines (https://link.springer.com/journal/44319/submission-guidelines#cms-Revised-submissions) and carefully review the instructions that follow below. Failure to include requested items will delay the evaluation of your revision.

1) a .docx formatted version of the manuscript text (including legends for main figures, EV figures and tables). Please make sure that the changes are highlighted to be clearly visible.

2) individual production quality figure files as .eps, .tif, .jpg (one file per figure). See hhttps://media.springernature.com/original/springer-cms/rest/v1/content/27825798/data/v1 for more info on how to prepare your figures.

3) We replaced Supplementary Information with Expanded View (EV) Figures and Tables that are collapsible/expandable online. A maximum of 5 EV Figures can be typeset. EV Figures should be cited as 'Figure EV1, Figure EV2" etc... in the text and their respective legends should be included in the main text after the legends of regular figures.

- For the figures that you do NOT wish to display as Expanded View figures, they should be bundled together with their legends in a single PDF file called *Appendix*, which should start with a short Table of Content. Appendix figures should be referred to in the main text as: "Appendix Figure S1, Appendix Figure S2" etc. See 'Expanded View' section of the submission guidelines.

- Additional Tables/Datasets should be labeled and referred to as Table EV1, Dataset EV1, etc. Legends have to be provided in a separate tab in case of .xls files. Alternatively, the legend can be supplied as a separate text file (README) and zipped together with the Table/Dataset file.

4) a .docx formatted letter INCLUDING the reviewers' reports and your detailed point-by-point responses to their comments. As part of the EMBO Press transparent editorial process, the point-by-point response is part of the Review Process File (RPF), which will be published alongside your paper.

5) a complete author checklist, which you can download from our author guidelines. Please insert information in the checklist that is also reflected in the manuscript. The completed author checklist will also be part of the RPF.

6) Please note that all corresponding authors are required to supply an ORCID ID for their name upon submission of a revised manuscript (<https://orcid.org/>), which can be linked to your profile in the manuscript submission system.

7) Before submitting your revision, primary datasets produced in this study need to be deposited in an appropriate public database. Please remember to provide a reviewer password if the datasets are not yet public. The accession numbers and database should be listed in a formal "Data Availability" section placed after Methods. Please note that the Data Availability Section is restricted to new primary data that are part of this study. * Note - All links should resolve to a page where the data can be accessed. *

If your study has not produced novel datasets, please mention this fact in the Data Availability Section.

8) At EMBO Press we ask authors to provide source data for the main manuscript figures. You will receive a separate email with instructions for providing source data with your revised manuscript, including how to upload and organize the files.

9) Our journal also encourages inclusion of *data citations in the reference list* to directly cite datasets that were re-used and obtained from public databases. Data citations in the article text are distinct from normal bibliographical citations and should directly link to the database records from which the data can be accessed. In the main text, data citations are formatted as follows: "Data ref: Smith et al, 2001" or "Data ref: NCBI Sequence Read Archive PRJNA342805, 2017". In the Reference list, data citations must be labeled with "[DATASET]". A data reference must provide the database name, accession number/identifiers and a resolvable link to the landing page from which the data can be accessed at the end of the reference.

10) Regarding data quantification, the following points must be specified in each figure legend:

- the name of the statistical test used to generate error bars and P values,

- the number (n) of independent experiments (please specify technical or biological replicates) underlying each data point,

- the nature of the bars and error bars (s.d., s.e.m.),

- If the data are obtained from n Program fragment delivered error ``Can't locate object method "less" via package "than" (perhaps you forgot to load "than"?) at //ejpvfs23/sites23b/embor_www/letters/embor_decision_revise_and_review.txt line 54.' 2, use scatter blots showing the individual data points.

Discussion of statistical methodology can be reported in the materials and methods section, but figure legends should contain a basic description of n, P and the test applied.

- Please also include scale bars in all microscopy images.

11) The journal requires a statement specifying whether or not authors have competing interests (defined as all potential or actual interests that could be perceived to influence the presentation or interpretation of an article). In case of competing interests, this must be specified in your disclosure statement.

12) All Materials and Methods need to be described in the main text using our 'Structured Methods' format, which is required for all research articles. According to this format, the Methods section includes a Reagents and Tools Table (listing key reagents, experimental models, software and relevant equipment and including their sources and relevant identifiers) followed by a Methods and Protocols section describing the methods using a step-by-step protocol format. The aim is to facilitate adoption of the methodologies across labs. More information on how to adhere to this format as well as a downloadable template (.docx) for the Reagents and Tools Table can be found in our author guidelines.

We would also welcome the submission of cover suggestions, or motifs to be used by our Graphics Illustrator in designing a cover.

As part of the EMBO publication's Transparent Editorial Process, EMBO reports publishes online a Review Process File (RPF) to accompany accepted manuscripts. This File will be published in conjunction with your paper and will include the referee reports, your point-by-point response and all pertinent correspondence relating to the manuscript.

You are able to opt out of this by letting the editorial office know (emboreports@embo.org). If you do opt out, the Review Process File link will point to the following statement: "No Review Process File is available with this article, as the authors have chosen not to make the review process public in this case."

I look forward to seeing a revised form of your manuscript when it is ready.

Yours sincerely,

Martina Rembold, PhD
Senior Editor
EMBO reports

==================

Referee #1:

In this study, Yan and colleagues identify a new mitochondrial pathway which can induce GSDME-dependent pyroptosis via activation of Parkin. In this pathway, they show that PTEN-L can act as an inhibitor, by dephosphorylating and inactivating Parkin, and disrupting the interaction between Parkin and MCL1. PTEN-L expression thus blocks MCL1 ubiquitination and degradation, blocking MOMP and pyroptosis.
The authors provide a lot of data to backup these findings. However, I have some suggestions for where the conclusions could be strengthened.
1. Throughout the authors claim that they see GSDME-dependent pyroptosis. This is based on the "bubble" morphology of the cells (e.g. in Figure 1A), and the cleavage of GSDME (Figure 2A) which is found at the plasma membrane (Figure 2H). siRNA against GSDME is used in Figure 2K to block this pyroptosis, but has minimal effects on LDH release (despite the significance), from ~25% to ~20% (Figure 2K). Does GSDME knockout completely block the pyroptotic morphology?
2. The timing of these events are also a bit puzzling to me. The authors clearly see cytochrome c release (evidence of MOMP) at 6h. According to established dogma, this should drive rapid caspase-3/7 activation and apoptotic cell death. Yet, it is not until 24h that GSDME cleavage is seen. What is happening in the hours between 6-24h? Why are the cells not simply undergoing apoptosis? The authors only knockdown BAX in Figure 3I - is the death completely blocked in BAX/BAK double knockdowns?
3. Along these lines, an outstanding question is why CCCP induces pyroptosis, and not apoptosis. Is it because of ROS levels in the cells? If so, does removing the ROS block the pyroptosis?
4. The data shown is all performed in HeLa cells, either with or without YFP-Parkin. It would be nice to see some key experiments also performed in other cells, particularly in cells which express endogenous levels of Parkin. Also, what happens in cells which do not express GSDME?
5. The authors show that MCL1 is rapidly degraded, but BCL2, BAX and BAK are not. MCL1 is known to have a very short half life - could this be the reason why MCL1 is seen to disappear from cells fast? PTEN-L overexpression also does not fully block the degradation of MCL1 (Figure 4A) - is there an explanation for this? There is also an increase in MCL1 degradation in vector cells compared to PTEN-L cells (Figure 2A+B), but could this simply be because the vector cells die more readily than the PTEN-L cells? Can these experiments be performed in BAX/BAK knockout cells, or caspase inhibitor to control for this? A lot of the data which uses the S63845 MCL1 inhibitor does not show data for S63845 alone, only DMSO, CCCP and CCCP+S63845. Given a dose of 3 µM S63845 (used here) is quite high, it would be important to see the effect of this alone. Similarly, in SFigure 5J, the authors show YFP-Parkin translocation to the mitochondria, but do not show CCCP alone. CCCP should cause YFP-Parkin translocation, so it is unclear what impact S63845 is really having here. What happens if MCL1 is knocked down, rather than inhibited?
6. What is the role of the MCL1-NOXA complex here? For instance, MCL1's degradation has been shown to dependent on NOXA following treatment with microtubule targeting agents. If MCL1 is inhibited or degraded, what happens to the NOXA? Does it induce MOMP? Does it bind A1? Does knocking down or knocking out NOXA increase resistance to CCCP-induced MOMP and cell death?

Minor points
• In Figure 2E, PTEN-L overexpression blocks GSDME cleavage after CCCP, and in Figure 2F PTEN-L KO activates GSDME cleavage. Does re-expression of PTEN-L in PTEN-L KO cells block this?
• In Figure 2M, the PTEN-L cells look a different morphology to the control cells. Is there a reason for this?
• In Figure 3C, the cytochrome c is seen outside the mitochondria. Usually cytochrome c is degraded quite rapidly in cells once it has exited the mitochondria. Is there an explanation for this?
• The IPs shown in FIgure 5H rely on transfections into HEK293 cells, which could influence the results. Is (for example) endogenous MCL1 detected in a FLAG-Parkin pulldown? Likewise, is it possible to test some endogenous protein pulldowns in figure 5J?

Referee #2:

This manuscript proposes that PTEN-L functions as a protein phosphatase for Parkin, regulating mitochondrial stress responses

and GSDME-dependent cell death. While the mechanism is potentially important, several conceptual and experimental issues require clarification before publication.
1. The physiological relevance of PTEN-L is unclear. Protective effects are primarily observed under overexpression, whereas endogenous PTEN-L appears to have limited impact. It may be that PTEN-L overexpression amplifies an otherwise ongoing process, but this point is neither clearly visualized nor explicitly explained.

2. Throughout the manuscript, the comparison between PTEN-L overexpression and knockout systems is confusing and potentially misleading. In overexpression experiments, the protective effect is attributed to PTEN-L relative to endogenous levels, implying that endogenous PTEN-L has little role. In knockout systems, however, the protective effect is attributed to endogenous PTEN-L, suggesting that it is functionally important. This discrepancy may be justified by the fact that the authors analyze different time points (typically ~24 h for overexpression and {less than or equal to}12 h for knockout). However, this makes it difficult to interpret whether PTEN-L truly has distinct effects or whether the observed differences are simply due to kinetics. Related, in Figure 1, comparing panels a-b to n-o, CCCP appears less effective. This is likely due to different time points (24 h vs 12 h). The authors should provide matched time-course analyses (e.g. 12 h and 24 h) comparing endogenous, overexpression, and knockout conditions to distinguish kinetic acceleration from mechanistic differences.

3. CCCP concentrations and timepoints vary across experiments without justification. A systematic time- and dose-response analysis would clarify interpretation, particularly given variable GSDME cleavage across cell lines. Time points (always) and concentrations (FigS7C, D, E, F I) should be clearly indicated in the figure itself.

4. The mechanistic relationship between PTEN-L, Parkin, and MCL-1 is not clearly resolved. It remains unclear whether PTEN-L directly stabilizes MCL-1 or indirectly does so via Parkin dephosphorylation. Co-immunoprecipitation does not establish direct functional interaction. Figure 7 oversimplifies this distinction and should be revised. BAK should also be included alongside BAX.

5. There is substantial conceptual confusion between apoptosis and pyroptosis. In lines 132 and 134, the authors refer to pyroptosis at a stage where only cell death has been demonstrated. If BAX/BAK pore formation occurs upstream, canonical apoptotic hallmarks (e.g. PARP cleavage) should be present. GSDME activation downstream of BAX/BAK implies that apoptosis precedes plasma membrane permeabilization. These processes cannot be treated as independent. The terminology "pyroptosis inducers" and "tBid/BAX-induced pyroptosis" is misleading and should be corrected.
The fact that compound S63845 alone does not induce a pyroptotic phenotype may depend on dosage, timing, or cellular caspase-3 levels, a possibility the authors themselves mention (line 340). This needs to be explicitly acknowledged and discussed, and the manuscript's conclusions revised accordingly.

6. The proposed two-hit model (lines 337-342) is overly compressed. The authors should clearly define the model, separate Parkin signaling from mitophagy, and justify the proposed caspase-3 threshold for GSDME cleavage. The apoptosis-pyroptosis switch may be context-dependent and should be discussed as such.

7. Specificity for PTEN-L over canonical PTEN is not sufficiently explained. A truncation analysis or at least a mechanistic rationale is needed.

Other points:
8. The Introduction inaccurately states that GSDME integrates into mitochondria (Rogers et al. demonstrated localization, not integration). The transition to GSDME is abrupt and should include clarification of gasdermin family function and lytic vs non-lytic death.
9. Several figures are overly dense or redundant. The authors should consider moving OA treatments and Sytox assays to the Supplementary Information. SYTOX and PI are used in parallel without clear justification. Sample order inconsistencies and missing timepoint annotations reduce clarity.
10. Some data presentation requires clarification:
- Figure 2H: The localization of GSDME at mitochondria should be addressed.
- Figure 2I: the PTEN-L seems low expressed for the DMSO ctrl in SiGSDME cells. Why?
- Figure 2J: In CCCP-treated cells with GSDME knockdown, what is the cause of cell death? If PTEN-L still provides protection under these conditions, additional death pathways may be involved. This should be discussed.
- Figure S3K: Cleavage of GSDME is quite low in this cell line with CCCP compared to HeLa cells. What could be the reason? Why did the authors choose this timepoint and a higher concentration of CCCP (40µM compared to the usual 20µM?)
- I would suggest to remove Figure 3A and keep Figure 3B as they prvide the same information.
- Figure 4g and related experiments: To demonstrate specificity for MCL-1, the authors should show that ABT-737 (which inhibits BCL-2 and BCL-xL but not MCL-1) does not reverse the PTEN-L effect. Lines 326-328: ABT-737 does not inhibit MCL-1. This statement is incorrect and should be corrected (see DOI: 10.1016/j.ccr.2006.08.027).
- Reproducibility of all western blot and imaging data should be explicitly stated in all figure and supplementary figure legends.
- Figures are sometimes incorrectly labeled in the text (e.g. S3).
- Cell lines used should be mentioned in the text not only in the figure legend.
- In Figure 7 the GSDME pore is depicted in the wrong direction (globular domain should face inside the cell).
11. Lines 175-177 and 203-205: To definitively demonstrate the exclusive role of cleaved GSDME, an inactive GSDME mutant should be used.

12. Line 308-309: The statement refers to Figure 5h but is supported by Figure 5j.
13. Lines 326-327: Sentence should be revised for clarity.
14. Line 400-403: clarify that this is under conditions of mitochondrial depolarization treatments
15. Important methodological details are missing (microscopy settings, mitochondrial fractionation protocol, gel percentages, SYTOX concentration, number of cells analyzed).

Re: MS EMBOR-2025-63377-T

Point-to-Point Responses to Reviewers' Comments

Referee #1:

In this study, Yan and colleagues identify a new mitochondrial pathway which can induce GSDME-dependent pyroptosis via activation of Parkin. In this pathway, they show that PTEN-L can act as an inhibitor, by dephosphorylating and inactivating Parkin, and disrupting the interaction between Parkin and MCL1. PTEN-L expression thus blocks MCL1 ubiquitination and degradation, blocking MOMP and pyroptosis.
The authors provide a lot of data to backup these findings. However, I have some suggestions for where the conclusions could be strengthened.

Response: We are grateful to the Reviewer for your nice summary, positive evaluation, and constructive suggestions to our work. We have thoroughly revised our manuscript in response, as detailed below.

1. Throughout the authors claim that they see GSDME-dependent pyroptosis. This is based on the "bubble" morphology of the cells (e.g. in Figure 1A), and the cleavage of GSDME (Figure 2A) which is found at the plasma membrane (Figure 2H). siRNA against GSDME is used in Figure 2K to block this pyroptosis, but has minimal effects on LDH release (despite the significance), from ~25% to ~20% (Figure 2K). Does GSDME knockout completely block the pyroptotic morphology?

Response: We sincerely appreciate the Reviewer for this constructive suggestion.
(1) To directly address whether GSDME knockout abolishes the pyroptotic morphology, we re-examined the cell morphology in our GSDME knockout cells following CCCP treatment. The bubble-like membrane protrusions characteristic of pyroptosis were clearly eliminated in GSDME knockout cells, accompanied by markedly reduced PI uptake and LDH release. These results are consistent with our siRNA knockdown data and confirm that the morphological features we observed are indeed GSDME-dependent. The new data have been included as Appendix Fig. S5B-D for HeLa GSDME knockout cells and Appendix Fig. S7I-K for SH-SY5Y GSDME knockdown cells.

(2) Regarding the Reviewer's observation about the relatively modest LDH reduction (~25% to ~20%) in the PTEN-L overexpression background shown in Fig. 2K (now presented as Fig. 2H). We would like to point out that in the vector control group, LDH release decreased from ~45% to ~28%, a more substantial reduction, under the same conditions with PTEN-L overexpression. To further confirm the reproducibility of these results, we repeated the experiment three more times. The new data showed a reduction from ~52% to ~24% in the vector control group and from ~26% to ~22% in the PTEN-L overexpression group, consistent with our original findings. The new data have been

included below as Rev Fig. 1 for the reference.

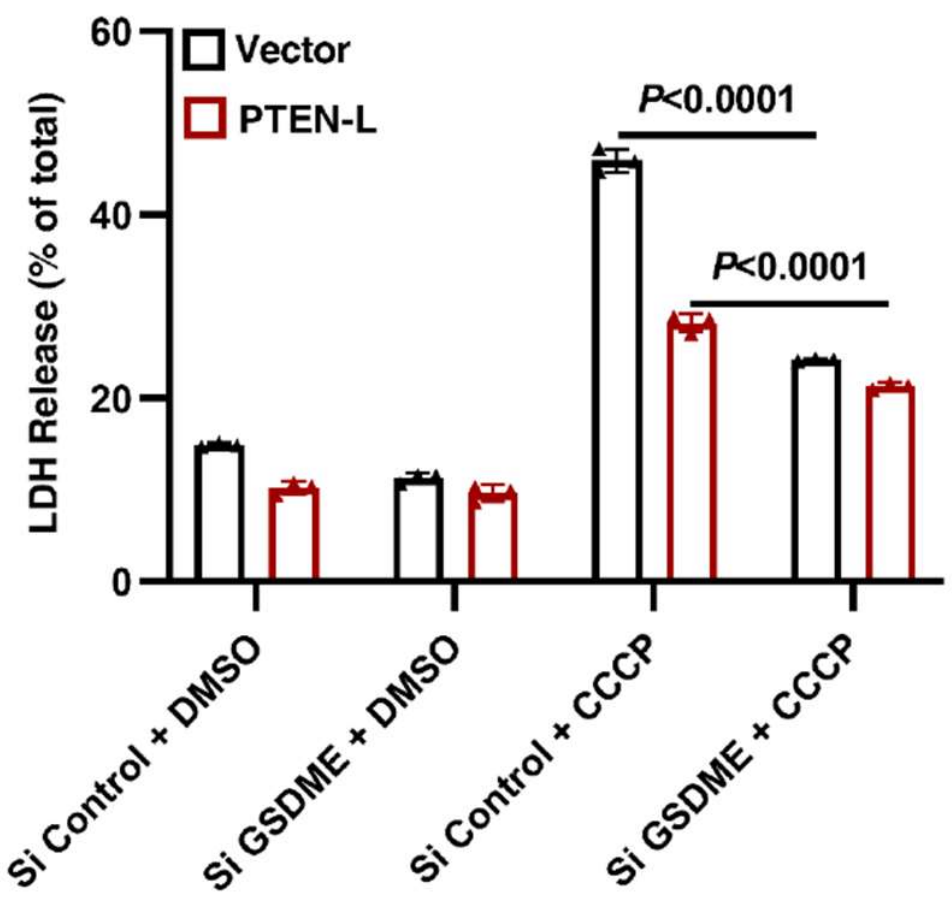

Rev Figure 1-Evaluation of LDH Release in Si GSDME cells. LDH release assay of HeLa YFP-Parkin Vector/PTEN-L cells transfected with nontargeting control siRNA (Si Control) or siRNA targeted against GSDME (Si GSDME) treated with 20 μM CCCP for 16 h. Data are presented as mean ± SD from three independent biological replicates. Statistical significance was assessed by two-way ANOVA.

We believe that the smaller reduction in the PTEN-L overexpression background is reasonable. This is because PTEN-L overexpression itself already strongly inhibits GSDME cleavage and pyroptosis. Under these circumstances, the baseline level of GSDME-mediated pyroptosis is low, which leaves limited scope for further suppression by GSDME knockdown. The fact that the reduction is small in this context does not undermine the conclusion; instead, it is consistent with PTEN-L acting upstream to restrict GSDME activation. Importantly, the substantial reduction in LDH release observed in the vector control group, where GSDME is actively cleaved, indicates that GSDME is the primary executor of pyroptosis in our system.

2. The timing of these events are also a bit puzzling to me. The authors clearly see cytochrome c release (evidence of MOMP) at 6h. According to established dogma, this should drive rapid caspase-3/7 activation and apoptotic cell death. Yet, it is not until 24h that GSDME cleavage is seen. What is happening in the hours between 6-24h? Why are the cells not simply undergoing apoptosis? The authors only knockdown BAX in Figure 3I - is the death completely blocked in BAX/BAK double knockdowns?

Response: We thank the Reviewer for raising these important points. We have addressed each of the points raised, as detailed below.

(1) Regarding the events between 6 and 24 hours

We agree with the Reviewer that we are unable to entirely rule out the possibility of apoptosis occurring at early time points following CCCP treatment. To better understand the temporal progression of cell death in our system, we conducted a comprehensive time-course analysis examining cell morphology, PI uptake, LDH release, caspase-3 activity, and GSDME cleavage. At early time points (6-9 h), we noted sporadic cells presenting apoptotic morphology, along with slight increases in PI uptake, LDH release, and caspase-3 activity. From 9 to 12 h, cells started to show pyroptotic morphology, with more significant increases in these parameters and the emergence of

GSDME-N cleavage fragments. By 24 h, the majority of cells exhibited pronounced pyroptotic morphology, accompanied by robust GSDME cleavage and massively elevated pyroptotic markers. The new data have been included as Appendix Fig. S9B-G.

These observations suggest that when GSDME is present, a moderate degree of mitochondrial damage initially triggers a low level of caspase-3 activation sufficient for mild apoptosis. However, when the damage becomes more severe or persistent, caspase-3 activity rises to a level that efficiently cleaves GSDME, shifting the cell death outcome toward pyroptosis. This is consistent with the concept of a caspase-3 activity threshold for GSDME cleavage, which we now discuss in the revised manuscript at line 292 to 317, on page 14 and 15.

(2) Regarding BAX/BAK double knockdown

As suggested by the Reviewer, we generated stable BAX/BAK double knockdown cells and examined cell death morphology, GSDME cleavage, and SYTOX Green uptake following CCCP treatment. Consistent with the Reviewer's expectation, BAX/BAK double knockdown completely blocked CCCP-induced cell death. These results confirm that the mitochondrial pathway mediated by BAX/BAK is crucial for the pyroptotic cell death we observed. The new data have been added as Fig. 3G-K.

In addition, we have revised Fig. 7 to incorporate BAK along with BAX in the schematic model.

(3) Regarding the Reviewer's comment on the 6 h cytochrome c release data

We sincerely apologize for an inaccuracy in our previous flow cytometry analysis. Now, we re-examined our cytochrome c release data following the approach described in Pan et al. (Cell, 2022; PMID: 35447071) [1]. After re-analysis using the correct gating strategy, we found that approximately 7% of cells exhibited cytochrome c release at 6 hours, which is consistent with our immunofluorescence imaging results. The new analyzed data was presented as Appendix Fig. S8E. We propose that this low percentage of cytochrome c may serve as evidence of "minority MOMP", a sublethal mitochondrial permeabilization event affecting only a fraction of mitochondria [2,3]. Under these conditions, the limited cytochrome c release leads to a low level of caspase-3 activation, which is insufficient to trigger extensive cell death but may prime the cells for subsequent responses. As mitochondrial damage accumulates over time, caspase-3 activity gradually rises beyond the threshold required for GSDME cleavage, ultimately committing the cells to pyroptosis. This threshold model is further supported by our time-course data showing the progressive increase in caspase-3 activity and the delayed appearance of GSDME cleavage. The new data have been added as Appendix Fig. S9B-G.

(4) Regarding the type of cell death

To further confirm the nature of the cell death we observed, we performed high-

resolution scanning electron microscopy (SEM) to examine the ultrastructural morphology of CCCP-treated cells. In vector control cells, we clearly observed numerous bubble-like protrusions (pyroptotic bodies, indicated by red circles) on the cell surface, along with visible membrane pores and plasma membrane disruption (red circles). In contrast, PTEN-L OE effectively prevented these pyroptotic features. These ultrastructural observations provide further direct visual evidence that the cell death induced by CCCP in our system is indeed pyroptosis. The new data have been added as Fig. 1D.

3. Along these lines, an outstanding question is why CCCP induces pyroptosis, and not apoptosis. Is it because of ROS levels in the cells? If so, does removing the ROS block the pyroptosis?

Response: We thank the Reviewer for this important advice. Our data suggest that two pivotal factors determine the reason why CCCP predominantly triggers pyroptosis instead of apoptosis in our system: the presence of GSDME and the degree of mitochondrial damage.

First, the expression of GSDME is essential for the pyroptotic outcome. In cells where GSDME is absent (either through knockout or knockdown), treatment with CCCP induced apoptosis rather than pyroptosis, as demonstrated in our HeLa, SH-SY5Y, and HEK293T cells. This is in line with the well-established role of GSDME in converting caspase-3-mediated apoptosis to pyroptosis when GSDME is present and cleaved [4-6]. The new data have been added as Appendix Fig. S5B-D and Appendix Fig. S9A for HeLa cells, Appendix Fig. S7I-K for SH-SY5Y cells, and Appendix Fig. S9H, K for HEK293T cells.

Second, we fully agree with the Reviewer that ROS play a critical role in this process. As the Reviewer suggested, we pre-treated cells with the ROS scavenger N-acetylcysteine (NAC) before CCCP exposure. We observed that CCCP treatment notably increased ROS levels in vector control cells. Significantly, treatment with the ROS scavenger NAC substantially reversed CCCP-induced pyroptosis, as evidenced reduced cell swelling, decreased PI uptake, and diminished GSDME cleavage (Appendix Fig. S10A-E). This observation is consistent with multiple studies demonstrating that ROS accumulation promotes GSDME-dependent pyroptosis through the mitochondrial pathway [7-9].

In addition, we have incorporated a discussion of the role of ROS as a critical mitochondrial damage signal in promoting GSDME-dependent pyroptosis to the revised manuscript at line 319 to 324, on page 15.

4. The data shown is all performed in HeLa cells, either with or without YFP-Parkin. It would be nice to see some key experiments also performed in other cells, particularly in cells which express endogenous levels of Parkin. Also, what happens in cells which

do not express GSDME?

Response: We thank the Reviewer for these constructive suggestions. Following the suggestion, we have now extended our key findings to SH-SY5Y cells, which endogenously express Parkin, and HEK293T cells.

(1) Regarding experiments in cells with endogenous Parkin

We generated SH-SY5Y cells with stable overexpression of PTEN-L. In these cells, we found that PTEN-L overexpression significantly suppressed CCCP-induced pyroptosis and inhibited GSDME cleavage. Conversely, our original data showed that PTEN-L knockout in SH-SY5Y cells markedly enhanced pyroptosis (Appendix Fig. S7F-H). These results are completely consistent with our observations in HeLa YFP-Parkin cells and confirm that the regulatory role of PTEN-L is not restricted to HeLa cells or to Parkin-overexpressing systems. The new data have been added as Appendix Fig. S7C-E.

(2) Regarding cells lacking GSDME and other cells

In HeLa YFP-Parkin cells, either knockout or knockdown of GSDME led to the abolition of pyroptosis and shifted cell death towards apoptosis (Fig. 2G, H, Appendix Fig. S5B-D and Appendix Fig. S9A). The new data have been added as Appendix Fig. S5B-D and Appendix Fig. S9A.

To verify this in a different cellular context, we generated stable GSDME knockdown SH-SY5Y cells. Similar to the situation in HeLa cells, the depletion of GSDME in SH-SY5Y cells changed CCCP-induced cell death from pyroptosis to apoptosis. To further verify this in a different cellular context, we also examined HEK293T cells, which have been reported to lack endogenous GSDME expression. In these cells, CCCP treatment induced apoptosis. However, when GSDME was ectopically expressed in HEK293T cells, CCCP treatment switched the cell death modality to pyroptosis; and more importantly this effect was inhibited by PTEN-L overexpression. These findings are consistent with previous reports indicating that GSDME is necessary for pyroptosis and that its absence causes cell death to default to apoptosis [4-6]. The new data have been added as Appendix Fig. S7I-K and Fig. S9H-L.

Together, these results demonstrate that our conclusions are not cell line-specific, and GSDME serves as the key executor of the pyroptotic cell death observed in our system across diverse cellular backgrounds.

5. The authors show that MCL1 is rapidly degraded, but BCL2, BAX and BAK are not. MCL1 is known to have a very short half life - could this be the reason why MCL1 is seen to disappear from cells fast? PTEN-L overexpression also does not fully block the degradation of MCL1 (Figure 4A) - is there an explanation for this? There is also an increase in MCL1 degradation in vector cells compared to PTEN-L cells (Figure 2A+B), but could this simply be because the vector cells die more readily than the PTEN-L

cells? Can these experiments be performed in BAX/BAK knockout cells, or caspase inhibitor to control for this? A lot of the data which uses the S63845 MCL1 inhibitor does not show data for S63845 alone, only DMSO, CCCP and CCCP+S63845. Given a dose of 3 µM S63845 (used here) is quite high, it would be important to see the effect of this alone. Similarly, in SFigure 5J, the authors show YFP-Parkin translocation to the mitochondria, but do not show CCCP alone. CCCP should cause YFP-Parkin translocation, so it is unclear what impact S63845 is really having here. What happens if MCL1 is knocked down, rather than inhibited?

Response: We thank the Reviewer for these thoughtful and constructive comments. We have addressed each point below.

(1) MCL-1 short half-life

We agree with the Reviewer. MCL-1 is known to have a short half-life due to its PEST-rich sequences that target it for rapid proteasomal degradation [10]. In our system, we think two factors contribute to the rapid degradation of MCL-1 upon CCCP treatment: its intrinsically short half-life, and the fact that mitochondrial depolarization activates Parkin, which ubiquitinates MCL-1 and targets it for accelerated proteasomal degradation. Consistent with this, treatment with the proteasome inhibitor MG132 significantly inhibited CCCP-induced MCL-1 degradation (Fig. 5B-E), confirming that the rapid loss of MCL-1 is primarily mediated by the ubiquitin-proteasome pathway.

(2) PTEN-L overexpression does not fully block MCL-1 degradation

We agree that PTEN-L overexpression does not fully block MCL-1 degradation. While Parkin is the major E3 ligase for MCL-1 upon mitochondrial depolarization, we noted that even in the absence of Parkin, MCL-1 still underwent some degradation (Figure 5F, G), suggesting the involvement of additional E3 ligases. To test this, we treated cells with TAK-243, a specific inhibitor of the E1 ubiquitin-activating enzyme. TAK-243 completely blocked MCL-1 degradation regardless of Parkin or PTEN-L status. The new data have been added as Appendix Fig. S14A-E.

Furthermore, we co-transfected PTEN-L with either Parkin or the corresponding empty vectors and treated the cells with CCCP. In the absence of Parkin, MCL-1 exhibited similar levels of degradation with or without PTEN-L expression. When Parkin was expressed and phosphorylated upon CCCP treatment, MCL-1 was robustly degraded. However, when both Parkin and PTEN-L were expressed, the phosphorylation of Parkin was markedly reduced, and the degradation of MCL-1 was significantly suppressed. To further validate this mechanism, we introduced the phospho-mimetic Parkin S65D mutant, which cannot be dephosphorylated by PTEN-L. In the absence of PTEN-L, both wild-type Parkin and Parkin S65D promoted MCL-1 degradation. In the presence of PTEN-L, wild-type Parkin no longer promoted MCL-1 degradation, whereas the S65D mutant remained active and continued to promote degradation. The new data have been added as Fig. 6E, F and Appendix Fig. S16B, C.

Collectively, these data further demonstrate that PTEN-L mainly functions by dephosphorylating Parkin and inhibiting Parkin-mediated MCL-1 ubiquitination and degradation. However, it cannot impede the partial degradation of MCL-1 mediated by other E3 ligases. This explains why PTEN-L overexpression reduces, but does not fully abolish MCL-1 degradation.

(3) MCL-1 degradation in vector cells versus PTEN-L overexpression cells: is it simply due to more cell death?

We considered this possibility. To address it, following the suggestion, we treated cells with the caspase inhibitor Q-VD-OPh to block cell death and examined MCL-1 levels. Even with Q-VD-OPh present, MCL-1 degradation in vector cells remained significantly greater than in PTEN-L overexpression cells. This indicates that the differential MCL-1 degradation between vector and PTEN-L overexpression cells is not merely a consequence of differential cell death. The new data have been added as Appendix Fig. S11C, D.

(4) S63845 alone controls and concentration, and CCCP controls

(A) We apologize for the oversight of these controls. As the Reviewer suggested, we have repeated the relevant experiments and now include the S63845 alone and CCCP alone controls in the revised manuscript. CCCP treatment induced Parkin translocation to mitochondria and increased Parkin phosphorylation at Ser65, consistent with its role in activating the PINK1-Parkin axis. Notably, S63845 treatment, when combined with CCCP, further enhanced Parkin mitochondrial translocation and phosphorylation, suggesting that MCL-1 inhibition amplifies mitochondrial damage and Parkin activation. The new data have been added as Fig. 4G-L, Appendix Fig. S11E-I, and Appendix Fig. S15A-D.

(B) Regarding the Reviewer's concern about the 3 μM concentration of S63835

S63845 has a Kd of 0.19 nM for human MCL-1, and concentrations ranging from 1 to 5 μM are commonly used in the literature [11, 12]. To directly address this, we repeated key experiments using 1 μM S63845 and obtained results comparable to those with 3 μM. Thus, the conclusions drawn from the S63845 experiments are not dependent on using a high concentration. The new data have been included below as (Rev Fig. 2A-D).

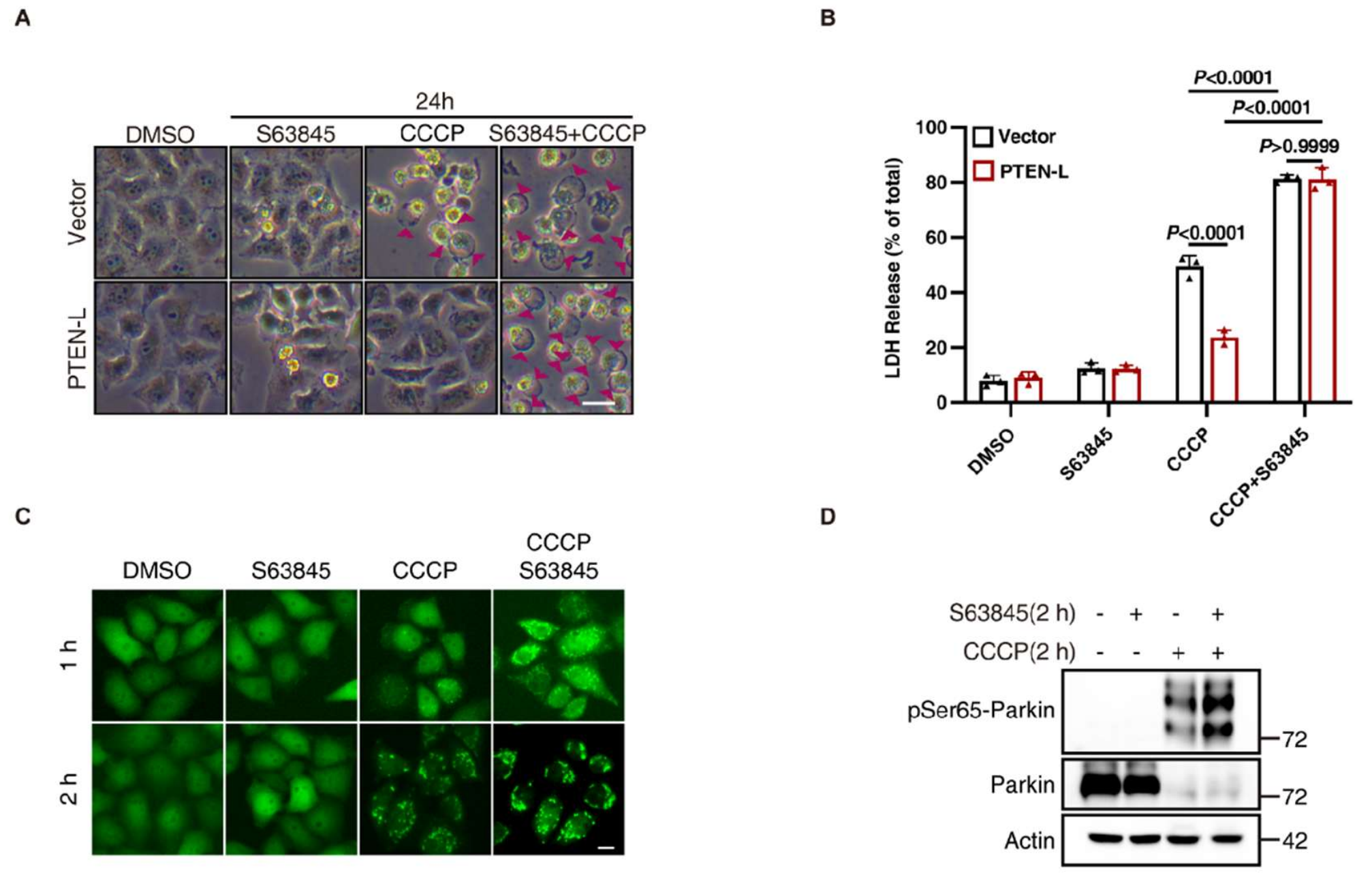

Rev Figure 2-MCL-1 inhibition induces pyroptosis following mitochondrial depolarization. (A and B). Representative microscopy images and LDH release assay of HeLa YFP-Parkin Vector/PTEN-L cells treated with 1 μM S63845 in combination or not with 20 μM CCCP for 24 h. Red arrows indicates pyroptotic cells. Scale bar, 25 μm. **(C and D).** Representative microscopy images of Parkin translocation and immunoblotting analysis of HeLa YFP-Parkin cells treated with 1 μM S63845 in combination or not with 20 μM CCCP for 2 h. Data are presented as mean ± SD from three independent biological replicates. Statistical significance was assessed by two-way ANOVA.

(5) MCL-1 knockdown versus inhibition

As the Reviewer suggested, we generated stable MCL-1 knockdown cell lines and examined pyroptotic morphology, LDH release, SYTOX Green uptake, BAX activation, GSDME cleavage, Parkin mitochondrial translocation, and Parkin phosphorylation. MCL-1 knockdown phenocopied the effects of S63845 treatment, which abolished PTEN-L-mediated cytoprotection and restored pyroptosis. These results confirm that the effects observed with S63845 are indeed due to MCL-1 loss of function, rather than off-target effects of the inhibitor. The new data have been added as Appendix Fig. S12A-G and Appendix Fig. S15G, H.

Collectively, our data show that S63845 treatment or MCL-1 knockdown, when combined with CCCP, exacerbates mitochondrial damage, accelerates Parkin translocation to mitochondria and its phosphorylation-dependent activation, and potentiates pyroptosis. These findings are consistent with earlier reports that MCL-1 can negatively regulate Parkin activity [13]. More importantly, MCL-1 inhibition or knockdown reverses the cytoprotective effect of PTEN-L, placing MCL-1 downstream of PTEN-L in this regulatory axis.

6. What is the role of the MCL1-NOXA complex here? For instance, MCL1's degradation has been shown to dependent on NOXA following treatment with microtubule targeting agents. If MCL1 is inhibited or degraded, what happens to the NOXA? Does it induce MOMP? Does it bind A1? Does knocking down or knocking

out NOXA increase resistance to CCCP-induced MOMP and cell death?

Response: We thank the Reviewer for raising these important questions.

To determine whether NOXA plays a role in our system, we initially examined the levels of NOXA protein after CCCP treatment. It was evident that NOXA was upregulated upon CCCP treatment, regardless of PTEN-L expression. Subsequently, we conducted immunoprecipitation experiments for endogenous proteins and discovered that CCCP treatment enhanced the interaction between MCL-1 and NOXA, as well as between MCL-1 and A1 (BFL-1). These findings are consistent with the well-known ability of NOXA to bind MCL-1 and A1, and with the reported role of NOXA in facilitating MCL-1 degradation [14, 15]. The new data have been added as Appendix Fig. S13A-C.

To directly examine whether NOXA contributes to CCCP-induced MCL-1 degradation, MOMP, and cell death, we generated stable NOXA knockdown cells. NOXA knockdown notably inhibited MCL-1 degradation, decreased BAX activation, and suppressed CCCP-induced pyroptosis, as demonstrated by the morphology changes, reduced LDH release, decreased SYTOX Green uptake, and diminished GSDME cleavage, both in the presence and absence of PTEN-L expression. These results position NOXA upstream of PTEN-L in this pathway. NOXA promotes MCL-1 degradation, presumably by binding to MCL-1 and facilitating its ubiquitination and proteasomal turnover. PTEN-L acts downstream by dephosphorylating Parkin and inhibiting MCL-1 ubiquitination. The new data have been added as Appendix Fig. S13D-K.

Regarding the Reviewer's question about whether NOXA binds A1, we conducted reciprocal co-immunoprecipitation experiments using antibodies against NOXA and A1. Regrettably, we failed to obtain definitive results, presumably because the antibodies were inappropriate for immunoprecipitation assays. However, our MCL-1 immunoprecipitation data offer robust indirect evidence. We noted that CCCP treatment augmented the interaction between MCL-1 and NOXA, as well as between MCL-1 and A1. Since it is well-documented that NOXA selectively binds to MCL-1 and A1, and its direct binding to A1 has been demonstrated [14], our data strongly corroborate the idea that NOXA and A1 associate with MCL-1 in a CCCP-inducible manner. Thus, we have recognized this technical limitation and clarified the interpretation in the revised manuscript at line 588 to 602, on page 27 and 28.

Minor points

• In Figure 2E, PTEN-L overexpression blocks GSDME cleavage after CCCP, and in Figure 2F PTEN-L KO activates GSDME cleavage. Does re-expression of PTEN-L in PTEN-L KO cells block this?

Response: Thanks again for the Reviewer's comment. Yes, re-expression of PTEN-L in PTEN-L knockout cells not only blocked GSDME cleavage but also suppressed pyroptotic morphology and LDH release. The figures were presented as Fig. 1K, 1L, Appendix Fig. S3I-K, and Appendix Fig. S4H.

Moreover, in this revision, to examine the physiological relevance of endogenous PTEN-L, we generated two additional cell lines using CRISPR/Cas9 technology. The first is a PTEN-L only cell line, in which we mutated the ATG start codon of canonical PTEN to ATA, enabling only expression of PTEN-L rather than canonical PTEN. The second is a PTEN/PTEN-L double knockout (DKO) cell line, in which both isoforms are eliminated. By comparing these two cell lines, the PTEN-L only cells effectively serve as a system in which endogenous PTEN-L is restored in a background lacking both PTEN and PTEN-L. We found that PTEN-L only cells exhibited significantly lower levels of pyroptosis, LDH release, and GSDME cleavage compared to double knockout cells. These data indirectly demonstrate that restoration of endogenous PTEN-L is sufficient to suppress pyroptosis. The new data have been added as Fig. 2L-O, and Appendix Fig. S6D, E.

• In Figure 2M, the PTEN-L cells look a different morphology to the control cells. Is there a reason for this?

Response: We sincerely apologize for the subpar image quality in our original Fig. 2M. After a thorough re-examination of our original data, we have determined that, under normal culture conditions, the morphology of PTEN-L cells and control cells is essentially similar. The discrepancy in the originally presented image was probably attributable to an uneven field or an artifact during image acquisition. We have substituted the image with a more representative one that clearly demonstrates comparable morphology between the two groups. The new data have been added as Fig. 2J. Moreover, additional representative fields are provided in Rev. Fig. 3 for comparison purposes.

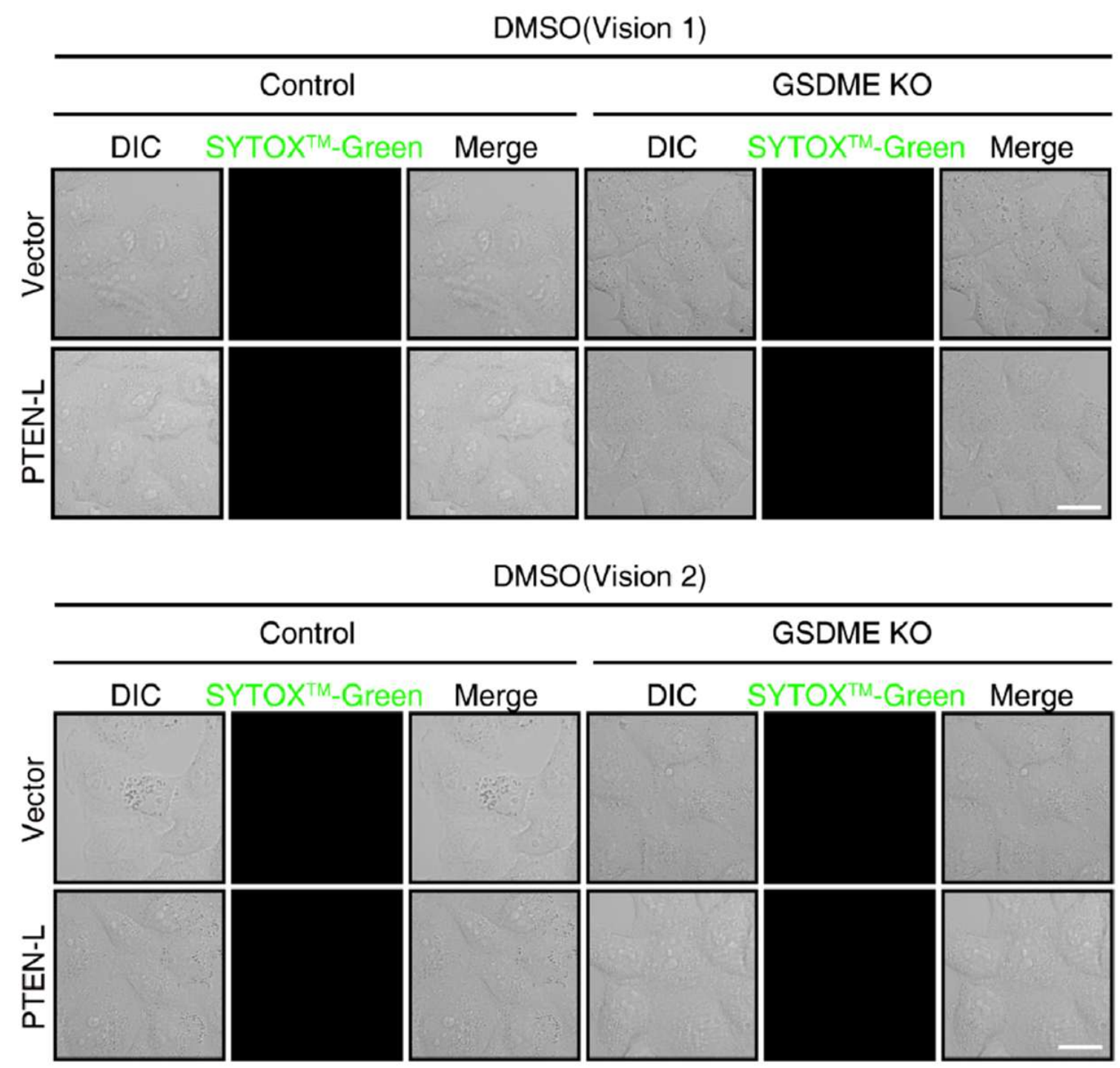

Rev Figure 3-Regarding the differences in DIC fields of vision between control cells and PTEN-L cells. Representative images of SYTOX Green staining in HeLa YFP-Parkin Vector/PTEN-L cells with GSDME knockout (GSDME KO) created via CRISPR/Cas9 system. Scale bars, 20 μm.

• In Figure 3C, the cytochrome c is seen outside the mitochondria. Usually cytochrome c is degraded quite rapidly in cells once it has exited the mitochondria. Is there an explanation for this?

Response: We thank the Reviewer for raising this point. We agree that under certain conditions, cytochrome c can undergo degradation upon its release from mitochondria. In post-mitotic neurons, PARC/CUL9 has been identified as an E3 ligase that targets cytochrome c for proteasome-mediated degradation, thus preventing apoptosis and facilitating cell survival [16]. In that study, the authors found that HeLa cells did not display endogenous degradation of mitochondrially released cytochrome c. Only overexpression of PARC/CUL9, rather than its endogenous expression, promoted cytochrome c degradation and cell survival after FCCP treatment, an agent comparable to CCCP (Figure 5C in PMID: 25028717) [17]. Conversely, cytochrome c accumulated in cells with low or absent PARC/CUL9 expression. Consistently, according to The Human Protein Atlas database, PARC/CUL9 expression is undetectable in HeLa cells, and another study also failed to detect its expression using PARC/CUL9 antibodies (Figure 1C in PMID: 33037272) [18]. Another possibility is that we are detecting an early, sublethal response after 6 hours of CCCP treatment. The low level of cytochrome c release results in weak caspase-3 activation that does not reach the threshold for extensive cell death or rapid protein degradation, as shown in Appendix Fig. S9B-G.

In addition, our results are consistent with numerous published studies. They also detected cytochrome c outside mitochondria through either immunofluorescence or by Western blot following mitochondrial fractionation after mitochondrial damage (Figure 1F in PMID 25739811; Figure 4M and 4N in PMID 34551295; Figure 3B in PMID 16888645) [19-21]. Therefore, we conclude that the presence of extra-mitochondrial cytochrome c at 6 h' treatment is not an experimental artifact. Instead, it reflects a kinetic window during which minority MOMP has occurred while the downstream lytic and degradative machinery has not yet been fully activated.

• The IPs shown in FIgure 5H rely on transfections into HEK293 cells, which could influence the results. Is (for example) endogenous MCL1 detected in a FLAG-Parkin pulldown? Likewise, is it possible to test some endogenous protein pulldowns in figure 5J?

Response: We thank the Reviewer for this constructive suggestion. Based on the suggestions, we have repeated the key immunoprecipitation experiments to detect endogenous proteins. In the revised experiments, we performed Flag-Parkin immunoprecipitation and found that endogenous MCL-1 was easily detected in the immunoprecipitates. In addition, we also examined the interaction between Flag-PTEN-L and endogenous MCL-1 and confirmed that they associate with each other (Fig. 5I; this is a new panel that was not included in the original submission). These results confirm that the interactions we observed are reproducible. The new data have been added as Fig. 5H and Appendix Fig. S14F.

We are grateful to the Reviewer for the careful and constructive evaluation of our work. The revisions have significantly strengthened the manuscript.

Referee #2:

This manuscript proposes that PTEN-L functions as a protein phosphatase for Parkin, regulating mitochondrial stress responses and GSDME-dependent cell death. While the mechanism is potentially important, several conceptual and experimental issues require clarification before publication.

Response: We thank the Reviewer for the positive feedback and all the insightful comments. We have revised our manuscript accordingly.

1. The physiological relevance of PTEN-L is unclear. Protective effects are primarily observed under overexpression, whereas endogenous PTEN-L appears to have limited

impact. It may be that PTEN-L overexpression amplifies an otherwise ongoing process, but this point is neither clearly visualized nor explicitly explained.

Response: We thank the Reviewer for raising this important point. We have carefully considered this issue and performed additional experiments to directly address the physiological relevance of endogenous PTEN-L.

(1) First, in our original submission, we have generated PTEN-L knockout cells using CRISPR/Cas9 in both HeLa and SH-SY5Y cells. PTEN-L knockout significantly accelerated CCCP-induced pyroptosis in both cell lines, as demonstrated by increased cell death and GSDME cleavage (Fig. 1H-J, Fig. 2D, 2E, Appendix Fig. S7F-H). Importantly, re-expression of PTEN-L in the knockout cells reversed this effect (Fig. 1K, 1L, Appendix Fig. S3I-K, and Appendix Fig. S4H). These experiments, including both loss-of-function and re-expression approaches, indicated that endogenous PTEN-L plays a genuine role in suppressing pyroptosis.

(2) Second, to further strengthen this conclusion, we generated two additional cell lines using CRISPR/Cas9 technology. The first is a PTEN-L only cell line, in which we mutated the ATG start codon of canonical PTEN to ATA, enabling only expression of PTEN-L rather than canonical PTEN. The second is a PTEN/PTEN-L double knockout (DKO) cell line, in which both isoforms are eliminated by targeting a shared region. We then systematically compared pyroptosis levels among wild-type cells (expressing both PTEN-L and PTEN), PTEN-L only cells, and double knockout cells. Wild-type and PTEN-L only cells displayed comparable levels of pyroptosis, as assessed by pyroptotic morphology, LDH release, and GSDME cleavage. In contrast, double knockout cells showed significantly elevated pyroptosis. These data strongly demonstrate that endogenous PTEN-L, in the absence of canonical PTEN, is sufficient to suppress pyroptosis, and that loss of endogenous PTEN-L sensitizes cells to pyroptotic cell death. The new data have been added as Fig. 2L-O, and Appendix Fig. S6D, E.

(3) Additionally, we also agree with the Reviewer that PTEN-L might also impact other ongoing processes. In the current study, we have not conducted a systematic exploration of these possibilities. In the revised manuscript, we have incorporated a discussion regarding this limitation and indicated that this remains an open question for future research at line 664 to 670, on page 31.

2. Throughout the manuscript, the comparison between PTEN-L overexpression and knockout systems is confusing and potentially misleading. In overexpression experiments, the protective effect is attributed to PTEN-L relative to endogenous levels, implying that endogenous PTEN-L has little role. In knockout systems, however, the protective effect is attributed to endogenous PTEN-L, suggesting that it is functionally important. This discrepancy may be justified by the fact that the authors analyze different time points (typically ~24 h for overexpression and {less than or equal to}12

h for knockout). However, this makes it difficult to interpret whether PTEN-L truly has distinct effects or whether the observed differences are simply due to kinetics. Related, in Figure 1, comparing panels a-b to n-o, CCCP appears less effective. This is likely due to different time points (24 h vs 12 h). The authors should provide matched time-course analyses (e.g. 12 h and 24 h) comparing endogenous, overexpression, and knockout conditions to distinguish kinetic acceleration from mechanistic differences.

Response: We thank the Reviewer for this critical observation and apologize for the confusion caused by using different time points for the overexpression and knockout experiments.

(1) We agree with the Reviewer that the apparent difference in CCCP efficacy between Fig.1 panels a-b (24 h) and original n-o (12 h) is mainly due to the different treatment durations, rather than a biological inconsistency. During this revision, to better understand the temporal progression of pyroptosis in our system, we performed a detailed time-course analysis examining cell morphology, PI uptake, LDH release, caspase-3 activity, and GSDME cleavage. The data showed that CCCP-induced pyroptosis increased progressively with treatment time. The new data have been added as Appendix Fig. S9B-G.

(2) Following the Reviewer's advice to directly address the concern, we performed matched time-course experiments comparing wild-type, PTEN-L overexpression, and PTEN-L knockout cells at 12 h and 24 h after CCCP treatment. We evaluated pyroptotic morphology, LDH release, and GSDME cleavage at both time points. As expected, the degree of pyroptosis was significantly higher at 24 h than at 12 h in all three conditions. At both time points, PTEN-L knockout cells consistently exhibited the highest level of pyroptosis, wild-type cells showed intermediate levels, and PTEN-L overexpression cells showed the lowest levels. The relative differences among the three genotypes were sustained at both time points. The new data have been added as Appendix Fig. S4I-K.

These well-matched time-course data indicate that the observed effects of PTEN-L are not simply due to differences in kinetics. Instead, PTEN-L overexpression consistently suppressed pyroptosis, whereas PTEN-L knockout promoted it, regardless of the time point examined. The comparison between overexpression and knockout systems is now clearer and fully supported by the well-matched data. We have incorporated these new data into the revised manuscript and updated the relevant figures accordingly.

3. CCCP concentrations and timepoints vary across experiments without justification. A systematic time- and dose-response analysis would clarify interpretation, particularly given variable GSDME cleavage across cell lines. Time points (always) and concentrations (FigS7C, D, E, F I) should be clearly indicated in the figure itself.

Response: We express our sincere gratitude to the Reviewer for this constructive suggestion. We deeply apologize for the lack of clearer justification regarding the

different CCCP concentrations and time points employed across the experiments. We agree with the Reviewer that a systematic analysis will enhance the interpretation of our data. As the Reviewer noted, different cell lines and genetic backgrounds exhibit variable sensitivity to CCCP-induced pyroptosis. These differences probably reflect variations in Parkin expression levels, which are closely linked to the degree of mitochondrial damage after CCCP treatment. To address these concerns, we have now performed a series of additional experiments.

(1) To better understand the temporal progression of pyroptosis in our system, we performed a detailed time-course analysis examining cell morphology, PI uptake, LDH release, caspase-3 activity, and GSDME cleavage. The data showed that CCCP-induced pyroptosis increased progressively with treatment time. The new data have been added as Appendix Fig. S9B-G.

Based on these observations, we then investigated whether the relative differences among wild-type, PTEN-L overexpression, and PTEN-L knockout cells remained consistent across time points. We performed matched time-course experiments in HeLa YFP-Parkin cells comparing these three genotypes at 12 h and 24 h after CCCP treatment. The degree of pyroptosis was greater at 24 h than at 12 h in all three conditions. However, even at the 12 h time point, PTEN-L knockout cells already showed significantly higher pyroptosis than wild-type cells, which accounts for our choice of the 12 h time point for the knockout experiments. The new data have been added as Appendix Fig. S4I-K.

(2) Regarding the concentrations, we newly generated SH-SY5Y cells stably expressing YFP-Parkin (SH-SY5Y YFP-Parkin). We then treated parental SH-SY5Y cells (which express endogenous Parkin) and SH-SY5Y YFP-Parkin cells with either 20 μM or 40 μM CCCP for 24 h and compared the levels of pyroptosis. SH-SY5Y cells expressing only endogenous Parkin displayed weaker pyroptosis than SH-SY5Y YFP-Parkin cells at both concentrations. To better understand the regulatory effect of PTEN-L on pyroptosis in cells with endogenous Parkin, we employed a higher concentration of CCCP (40 μM) for these experiments. This concentration was selected to ensure a robust pyroptotic response while maintaining the specificity of the effect. The new data have been added as Appendix Fig. S7A, B.

To further confirm that the chosen concentration and time points for SH-SY5Y cells produce consistent results, we treated these cells with 40 μM CCCP for 24 h and 48 h and examined pyroptotic cell death. The data showed that pyroptosis increased with longer treatment duration, consistent with our time-course observations in HeLa YFP-Parkin cells. The new data have been added as Appendix Fig. S9H.

(3) In addition, in response to the Reviewer's suggestion, we have clearly specified the treatment times in all relevant figure panels and CCCP concentrations (original Figures S7C, S7D, S7E, and S7F now are presented as Appendix Fig 17C-F).   For experiments

conducted under the same conditions, detailed time annotations are provided only in the bright-field images to save figure space. We believe these additions make the experimental conditions transparent and the results easier to interpret.

4. The mechanistic relationship between PTEN-L, Parkin, and MCL-1 is not clearly resolved. It remains unclear whether PTEN-L directly stabilizes MCL-1 or indirectly does so via Parkin dephosphorylation. Co-immunoprecipitation does not establish direct functional interaction. Figure 7 oversimplifies this distinction and should be revised. BAK should also be included alongside BAX.

Response: We sincerely thank the Reviewer for raising these important points. We have performed additional experiments to determine whether PTEN-L directly stabilizes MCL-1 or acts indirectly through Parkin dephosphorylation.

(1) To test whether PTEN-L directly stabilizes MCL-1, we co-transfected Flag-PTEN-L with either GST-Parkin or the corresponding empty vectors and treated the cells with CCCP. In the absence of Parkin, MCL-1 exhibited similar levels of partial degradation with or without PTEN-L expression. This indicates that PTEN-L does not directly stabilize MCL-1, which is consistent with our previous observation that other E3 ligases contribute to part of MCL-1 degradation independently of PTEN-L and Parkin. The new data have been added as Appendix Fig. S16B, C.

(2) To test whether PTEN-L indirectly stabilizes MCL-1 via Parkin dephosphorylation, we performed experiments in the presence of Parkin. When Parkin was expressed and phosphorylated upon CCCP treatment, MCL-1 was robustly degraded. However, when both Parkin and PTEN-L were expressed, the phosphorylation of Parkin was markedly reduced, and the degradation of MCL-1 was significantly suppressed. To further validate this mechanism, we introduced the phospho-mimetic Parkin S65D mutant, which cannot be dephosphorylated by PTEN-L. In the absence of PTEN-L, both wild-type Parkin and Parkin S65D promoted MCL-1 degradation. In the presence of PTEN-L, wild-type Parkin no longer promoted MCL-1 degradation, whereas the S65D mutant remained active and continued to promote degradation. These results demonstrate that PTEN-L stabilizes MCL-1 indirectly through dephosphorylation and inactivation of Parkin. The new data have been added as Fig. 6E, F and Appendix Fig. S16B, C.

(3) Following the Reviewer's suggestion, we have revised Fig. 7 to depict this indirect mechanism more clearly. In addition, we have included BAK alongside BAX in the schematic model. Moreover, to further support the functional involvement of BAX and BAK, we generated stable BAX/BAK double knockdown cells and examined cell death morphology, GSDME cleavage, and SYTOX Green uptake following CCCP treatment. The BAX/BAK double knockdown almost completely blocked CCCP-induced pyroptosis, confirming that the mitochondrial pathway mediated by BAX and BAK is crucial for the cell death we observed. The new data have been added as Fig. 3G-K.

5. There is substantial conceptual confusion between apoptosis and pyroptosis. In lines 132 and 134, the authors refer to pyroptosis at a stage where only cell death has been demonstrated. If BAX/BAK pore formation occurs upstream, canonical apoptotic hallmarks (e.g. PARP cleavage) should be present. GSDME activation downstream of BAX/BAK implies that apoptosis precedes plasma membrane permeabilization. These processes cannot be treated as independent. The terminology "pyroptosis inducers" and "tBid/BAX-induced pyroptosis" is misleading and should be corrected. The fact that compound S63845 alone does not induce a pyroptotic phenotype may depend on dosage, timing, or cellular caspase-3 levels, a possibility the authors themselves mention (line 340). This needs to be explicitly acknowledged and discussed, and the manuscript's conclusions revised accordingly.

Response: We thank the Reviewer for raising these important points. We have addressed each of them as detailed below.

(1) Conceptual confusion between apoptosis and pyroptosis

We agree with the Reviewer that we should not mention pyroptosis at a stage where only cell death has been demonstrated. We have made corresponding revisions to the text. At line 128 and 129, we changed "CCCP-induced cell death displayed characteristic pyroptotic morphology" to "CCCP-induced cell death displayed morphological features that are reminiscent of pyroptosis (now at line 130 and 131)." At lines 132 and 134, we replaced "pyroptosis" with "cell death (now at line 134, 136 and 137)" to avoid over-interpretation at this early stage.

(2) Relationship between apoptosis and pyroptosis

We agree with the Reviewer that apoptosis and pyroptosis are not independent processes when BAX/BAK-mediated MOMP occurs upstream. To better understand the temporal progression of cell death in our system, we performed a detailed time-course analysis examining cell morphology, PI uptake, LDH release, caspase-3 activity, and GSDME cleavage. At early time points (6-9 h), we observed occasional cells with apoptotic morphology, accompanied by a slight increase in PI uptake, LDH release, and caspase-3 activity. Between 9 and 12 h, we began to see cells displaying pyroptotic morphology, with more substantial increases in PI uptake and LDH release, further elevation of caspase-3 activity, and the emergence of GSDME-N cleavage fragments. By 24 h, the majority of cells exhibited pronounced pyroptotic morphology, with massively elevated PI uptake, LDH release, and caspase-3 activity, alongside robust GSDME cleavage. We also compared the cleavage of PARP and GSDME at 24 h after CCCP treatment. In cells expressing GSDME, PARP cleavage was relatively weak, whereas in GSDME knockout or deficient cells, PARP cleavage was markedly enhanced. This indicates that GSDME plays a critical role in redirecting the cell death outcome from apoptosis to pyroptosis, consistent with earlier studies [4-6]. Based on these observations, we have added a discussion of the relationship between apoptosis and pyroptosis in the revised manuscript at line 292 to 317 on page 14 and 15. The new data have been added as Appendix Fig. S9A-J.

Moreover, to further confirm the nature of the cell death we observed, we performed high-resolution scanning electron microscopy (SEM) to examine the ultrastructural morphology of CCCP-treated cells. In vector control cells, we clearly observed numerous bubble-like protrusions on the cell surface, along with visible membrane pores and plasma membrane disruption. In contrast, PTEN-L overexpression effectively prevented these pyroptotic features. These ultrastructural observations provide direct visual evidence that the cell death induced by CCCP at late time points in our system is indeed pyroptosis. The new data have been added as Fig. 1D.

(3) Misleading terminology "pyroptosis inducers" and "tBid/BAX-induced pyroptosis"

We thank the Reviewer for these corrections. We have revised the terminology throughout the manuscript as follows.

Line 139: "To investigate whether PTEN-L specifically suppresses pyroptosis triggered by mitochondrial depolarization, we treated cells with other stimuli known to induce pyroptosis, including tumor necrosis factor plus cycloheximide (TNFα plus CHX), Cisplatin, and Doxorubicin [37, 41] (now at line 144). "

Line 177: "This inhibitory effect was specific to mitochondrial stress, as PTEN-L did not alter GSDME cleavage induced by Cisplatin or Doxorubicin, and only slightly modulated it upon TNFα+CHX treatment (Figure S3A, B). This modest effect during TNFα+CHX treatment is likely mediated via caspase-3 activation after the engagement of the mitochondrial pathway (now at line 186)."

Line 361: "Notably, the increase in pSer65-Parkin was specifically associated with mitochondrial depolarization and was not observed upon treatment with other cell death inducing agents (now at line 500)."

(4) S63845 alone and the dependence on dosage, timing, or caspase-3 levels

We agree with the Reviewer that varying dosages or treatment durations of S63845, when leading to more severe mitochondrial damage, may potentially trigger pyroptosis. In the revised manuscript, we have included a discussion on this point at line 449 to 453, on page 21, and adjusted our conclusions accordingly. We now clearly state that the capacity of S63845 alone to induce pyroptosis might be contingent upon experimental conditions such as dosage, treatment duration, and cellular caspase-3 levels. If the mitochondrial damage caused by S63845 is severe enough, it is possible that the caspase-3 activity induced by S63845 could become sufficient for GSDME cleavage and pyroptosis induction.

We have integrated all these revisions into the manuscript and believe that they have significantly enhanced the clarity and accuracy of our presentation.

6. The proposed two-hit model (lines 337-342) is overly compressed. The authors should clearly define the model, separate Parkin signaling from mitophagy, and justify the proposed caspase-3 threshold for GSDME cleavage. The apoptosis-pyroptosis switch may be context-dependent and should be discussed as such.

Response: We thank the Reviewer for this great suggestion. We agree that the original description of the two-hit model was too brief. Currently, we have expanded this section to more precisely define the model, clearly distinguish Parkin signaling from mitophagy, justify the caspase-3 threshold, and thoroughly discuss the context-dependent nature of the apoptosis-pyroptosis switch. The revised description is now presented at line 458 to 473 on page 22.

In this revision, following the Reviewer's advice, we have also performed additional experiments to provide stronger data support for our model. We conducted time-course experiments and found that caspase-3 activity increased progressively with CCCP treatment, followed by GSDME cleavage and pyroptotic cell death. In cells lacking GSDME, caspase-3 activity was redirected toward PARP cleavage, resulting in apoptosis. These findings further provide strong evidence for the caspase-3 threshold model and confirm that GSDME diverts the cell death outcome from apoptosis to pyroptosis. The new data have been added as Appendix Fig. S9A-J.

7. Specificity for PTEN-L over canonical PTEN is not sufficiently explained. A truncation analysis or at least a mechanistic rationale is needed.

Response: We thank the Reviewer for raising this important point. To address the specificity of PTEN-L over canonical PTEN, we have performed experiments to directly compare their functions.

(1) We generated PTEN overexpression cells and treated them, along with control and PTEN-L overexpression cells, with CCCP. As the data demonstrated, PTEN-L overexpression significantly suppressed pyroptotic morphology, LDH release, and GSDME cleavage. In contrast, overexpression of canonical PTEN failed to inhibit any of these pyroptotic features. These results suggest that PTEN-L, rather than canonical PTEN, can suppress CCCP-induced pyroptosis. The new data have been added as Appendix Fig. S6A-C.

(2) To investigate the physiological relevance of endogenous PTEN-L in comparison with endogenous canonical PTEN, we generated two additional cell lines using CRISPR/Cas9 technology. The first is a PTEN-L only cell line, in which the ATG start codon of canonical PTEN was mutated to ATA, enabling expression of PTEN-L but not canonical PTEN. The second is a PTEN/PTEN-L double knockout cell line, in which both isoforms are eliminated by targeting a shared region. We then systematically compared pyroptosis levels among these cell lines. PTEN-L only cells exhibited significantly lower levels of pyroptosis, LDH release, and GSDME cleavage compared

to double knockout cells. These data demonstrate that endogenous PTEN-L, rather than canonical PTEN, is responsible for suppressing pyroptosis in our system. The new data have been added as Fig. 2L-O, and Appendix Fig. S6D, E.

We have incorporated these new data into the revised manuscript and added a mechanistic rationale for the specificity of PTEN-L over canonical PTEN in the Discussion section at line 649 to 655, on page 30 and 31.

Other points:
8. The Introduction inaccurately states that GSDME integrates into mitochondria (Rogers et al. demonstrated localization, not integration). The transition to GSDME is abrupt and should include clarification of gasdermin family function and lytic vs non-lytic death.

Response: We thank the Reviewer for these corrections.

(1) We have revised the inaccurate description regarding GSDME-N and mitochondria. The original text stated that GSDME-N "integrates into" the mitochondrial membrane. We have changed this to "can localize to" the mitochondrial membrane, which is more consistent with the findings reported by Rogers et al.

(2) We agree that the transition to GSDME was abrupt. We have added a brief clarification of gasdermin family function and the distinction between lytic and non-lytic cell death at lines 97 to 102, on page 5.

9. Several figures are overly dense or redundant. The authors should consider moving OA treatments and Sytox assays to the Supplementary Information. SYTOX and PI are used in parallel without clear justification. Sample order inconsistencies and missing timepoint annotations reduce clarity.

Response: We thank the Reviewer for these suggestions that can enhance the clarity of our figures. Following these suggestions:

(1) We have moved most of the OA treatment data and SYTOX Green staining results to the Supplementary Information.

(2) We sincerely apologize for the lack of a clear justification for the concurrent use of both SYTOX Green and PI. We have now added a detailed explanation in the Methods section at line 897 to 901, on page 47. Although both dyes are membrane-impermeant nucleic acid stains that detect plasma membrane permeabilization, in our study, PI staining was used for endpoint quantification by flow cytometry. In contrast, SYTOX Green staining was carried out in conjunction with confocal microscopy to concurrently visualize cell morphology and quantify dead cells within the same field of view.

(3) We have carefully reviewed all figures and corrected the inconsistencies in the sample order. We have revised the order of samples in original Fig. 2D (now presented as Appendix Fig. S4B), original Fig. 3I and J (now presented as Appendix Fig. S8H and I), Fig. 5E, original Fig.S3I (now presented as Appendix Fig. S5G), and Fig. S3N and P (now presented as Appendix Fig. S8K and L) to ensure consistency across the manuscript. Moreover, the treatment times have been clearly specified in all relevant figure panels.

We believe these changes have substantially improved the clarity and readability of our figures.

10. Some data presentation requires clarification:
- Figure 2H: The localization of GSDME at mitochondria should be addressed.

Response: We thank the Reviewer for this valuable suggestion. To investigate the localization of GSDME at mitochondria, we performed mitochondrial fractionation experiments. The data indicated that a portion of GSDME-N translocated to the mitochondrial fraction upon CCCP treatment. Importantly, PTEN-L overexpression prevented this translocation. We consider this result to be reasonable. Mitochondria are abundant in cardiolipin, which has been reported to facilitate the membrane binding of GSDME-N [22]. In addition, our observation is consistent with previous studies showing that GSDME-N can localize to mitochondria upon mitochondrial damage [6,23]. The new data have been added as Appendix Fig. S8B.

- Figure 2I: the PTEN-L seems low expressed for the DMSO ctrl in SiGSDME cells. Why?

Response: We apologize for this error. The weak PTEN-L signal in the DMSO control lane of the original Figure 2I was attributed to a loading or transfer artifact during western blotting. To validate our results, we conducted the experiment independently three times. In all replicates, the expression levels of PTEN-L were comparable under all conditions. One representative replicate is now presented in the revised Appendix Fig. S5A, and the other two independent replicates are provided in below Rev. Fig. 4.

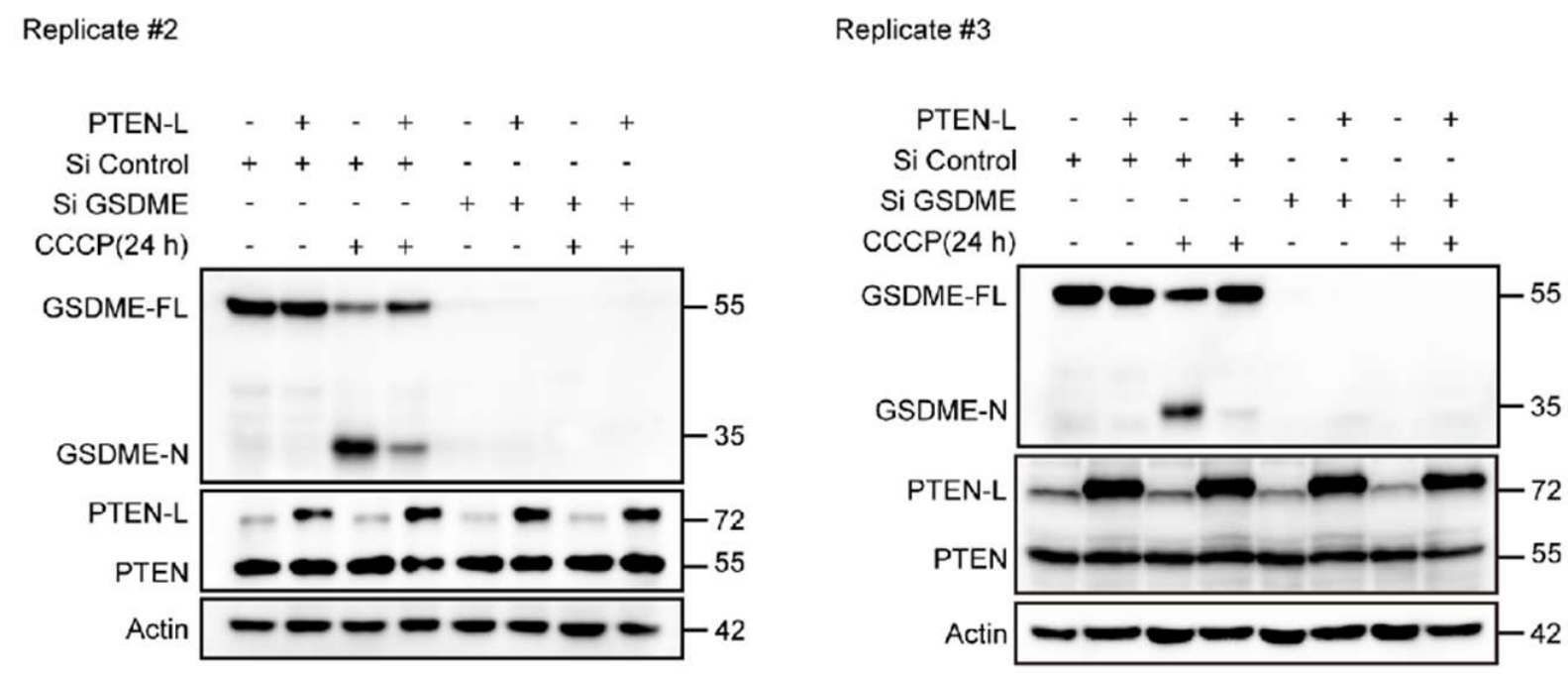

Rev Figure 4-Immunoblotting analysis of GSDME cleavage. Two replicates about immunoblotting analysis of GSDME cleavage and activation in lysates of HeLa YFP-Parkin Vector/PTEN-L cells transfected with nontargeting control siRNA (Si Control) or siRNA targeted against GSDME (Si GSDME) treated with 20 μM CCCP for 24 h.

- Figure 2J: In CCCP-treated cells with GSDME knockdown, what is the cause of cell death? If PTEN-L still provides protection under these conditions, additional death pathways may be involved. This should be discussed.

Response: We thank the Reviewer for this question. In GSDME knockdown or knockout cells, we consider that the residual cell death upon CCCP treatment is predominantly apoptosis. This is supported by our observation that PARP cleavage was markedly elevated in GSDME knockout or deficient cells compared to wild-type cells. The new data have been added as Appendix Fig. S9A, I. The protective effect of PTEN-L in GSDME-deficient cells likely reflects its ability to attenuate the upstream mitochondrial damage signal. By dephosphorylating Parkin, PTEN-L reduces the amplitude of the initial insult, which subsequently suppresses the caspase cascade. This upstream action explains why PTEN-L still offers partial protection in the absence of GSDME. The detailed molecular mechanism through which PTEN-L influences apoptosis under these conditions remain to be investigated. We have added a discussion of this point in the revised manuscript at line 664 to 670, on page 31.

- Figure S3K: Cleavage of GSDME is quite low in this cell line with CCCP compared to HeLa cells. What could be the reason? Why did the authors choose this timepoint and a higher concentration of CCCP (40μM compared to the usual 20μM?)

Response: We thank the Reviewer for this important question. We think this point is closely related to our response to Comment #3, and we hope the following will elucidate the matter.

The weaker GSDME cleavage observed in SH-SY5Y cells compared to HeLa YFP-Parkin cells is presumably attributed to lower endogenous Parkin expression in SH-SY5Y cells. Given that Parkin serves as a key amplifier of mitochondrial damage and the E3 ligase for MCL-1 degradation, its reduced expression leads to a decrease in the intensity of the pyroptotic signal. To verify this hypothesis, we established SH-SY5Y cells that stably express YFP-Parkin and compared them with the parental SH-SY5Y

cells, which only express endogenous Parkin. At concentrations of both 20 μM and 40 μM CCCP, the cells expressing YFP-Parkin exhibited more pronounced pyroptosis and GSDME cleavage than the parental cells. The new data have been added as Appendix Fig. S7A, B.

Regarding the time point selected for SH-SY5Y cells in original Figure S3K, our hypothesis was that PTEN-L knockout would promote pyroptosis. Thus, we selected a time point at which wild-type cells exhibited relatively weak pyroptosis, which enabled us to more effectively detect whether PTEN-L deficiency could accelerate the process. This reasoning is in line with our experimental design for the knockout experiments. We have also provided a more detailed explanation of our choices of time point and concentration in our response to Comment #3.

- I would suggest to remove Figure 3A and keep Figure 3B as they prvide the same information.

Response: We agree with the Reviewer. We have removed Figure 3A and kept Figure 3B in the revised manuscript.

- Figure 4g and related experiments: To demonstrate specificity for MCL-1, the authors should show that ABT-737 (which inhibits BCL-2 and BCL-xL but not MCL-1) does not reverse the PTEN-L effect. Lines 326-328: ABT-737 does not inhibit MCL-1. This statement is incorrect and should be corrected (see DOI: 10.1016/j.ccr.2006.08.027).

Response: We thank the Reviewer for this suggestion and correction.
(1) Following the Reviewer's advice, we treated PTEN-L overexpression and control cells with ABT-737 in combination with CCCP and found that ABT-737 did not reverse the protective effect of PTEN-L. This finding confirms that the protection mediated by PTEN-L is specific to MCL-1, rather than other anti-apoptotic BCL-2 family members such as BCL-2 or BCL-xL. The new data have been added as Appendix Fig. S15I, K, L.

(2) We have corrected the inaccurate statement regarding ABT-737 in the original manuscript. According to the reference provided by the Reviewer (Oltersdorf et al., Cancer Cell, 2006), ABT-737 is a BH3 mimetic that inhibits BCL-2, BCL-xL, and BCL-w, but does not inhibit MCL-1 [24]. We have revised the text accordingly at line 440, on page 21.

- Reproducibility of all western blot and imaging data should be explicitly stated in all figure and supplementary figure legends.

Response: We thank the Reviewer for this suggestion. We have revised the figure legends to clearly indicate that each key experiment was independently repeated at least three times.

- Figures are sometimes incorrectly labeled in the text (e.g. S3).

Response: We apologize for this error. We have corrected the original Figure S3 labeling in the text. Due to the addition of a large amount of new data and in order to achieve a more logical figure layout, the original Fig. S3 has now been reorganized and is presented as Fig. 2, Appendix Fig. S4, S5, S7 and S8. We have also carefully checked all other figure citations throughout the manuscript to minimize similar errors.

- Cell lines used should be mentioned in the text not only in the figure legend.

Response: We thank the Reviewer for this suggestion. We have now included the cell line information in the main text for all the experiments, rather than only in the figure legends.

- In Figure 7 the GSDME pore is depicted in the wrong direction (globular domain should face inside the cell).

Response: We thank the Reviewer for this professional suggestion. We have revised Fig. 7 to correct the orientation of the GSDME pore, with the globular domain now facing the inside of the cell.

11. Lines 175-177 and 203-205: To definitively demonstrate the exclusive role of cleaved GSDME, an inactive GSDME mutant should be used.

Response: We thank the Reviewer for this important suggestion. To unequivocally demonstrate that cleaved GSDME is indispensable for the pyroptotic cell death we observed, we conducted rescue experiments in GSDME knockout cells. We re-introduced either wild-type GSDME or a cleavage-resistant GSDME mutant (D267/270A), which is unable to be processed by caspase-3. Following CCCP treatment, cells reconstituted with wild-type GSDME displayed pyroptotic morphology, elevated LDH release, and substantial GSDME cleavage. Conversely, cells expressing the D267/270A mutant did not exhibit any of these characteristics. These results firmly confirm that caspase-3 mediated cleavage of GSDME is absolutely necessary for pyroptosis in our system. The new data have been added as Appendix Fig. S5H-J.

12. Line 308-309: The statement refers to Figure 5h but is supported by Figure 5j.

Response: We thank the Reviewer for this correction. We have revised the text to ensure that Fig. 5H is now used to describe the interaction between Parkin and MCL-1, whereas Fig. 5J is cited for the ubiquitination data. This revision improves the accuracy of our description.

13. Lines 326-327: Sentence should be revised for clarity.

Response: We thank the Reviewer for this suggestion. We have revised the sentence to improve its clarity. The updated text is now at line 440 to 447 on page 21.

14. Line 400-403: clarify that this is under conditions of mitochondrial depolarization treatments.

Response: We thank the Reviewer for this suggestion. We have revised the sentence to clarify that these events occur under conditions of mitochondrial depolarization. The updated text is now at line 538 on page 25.

15. Important methodological details are missing (microscopy settings, mitochondrial fractionation protocol, gel percentages, SYTOX concentration, number of cells analyzed).

Response: We thank the Reviewer for this important suggestion. We have thoroughly revised the Methods section to incorporate the missing details, such as microscopy settings, the mitochondrial fractionation protocol, gel percentages used in Western blotting, SYTOX Green concentration, and the number of cells analyzed in each experiment. Additionally, we have carefully reviewed the entire Methods section to guarantee that all experimental details are precisely described.

We thank the Reviewer again for the thorough and professional comments. The revisions made in response to these suggestions have substantially improved our manuscript.

References

1 Pan, R., Ryan, J., Pan, D., Wucherpfennig, K. W. & Letai, A. Augmenting NK cell-based immunotherapy by targeting mitochondrial apoptosis. *Cell* **185**, 1521-1538.e1518 (2022). https://doi.org/10.1016/j.cell.2022.03.030

2 Ichim, G. *et al.* Limited mitochondrial permeabilization causes DNA damage and genomic instability in the absence of cell death. *Mol Cell* **57**, 860-872 (2015). https://doi.org/10.1016/j.molcel.2015.01.018

3 Häcker, G. & Haimovici, A. Sub-lethal signals in the mitochondrial apoptosis apparatus: pernicious by-product or physiological event? *Cell death and differentiation* **30**, 250-257 (2023). https://doi.org/10.1038/s41418-022-01058-0

4 Wang, Y. *et al.* Chemotherapy drugs induce pyroptosis through caspase-3 cleavage of a gasdermin. *Nature* **547**, 99-103 (2017). https://doi.org/10.1038/nature22393

5 Rogers, C. *et al.* Cleavage of DFNA5 by caspase-3 during apoptosis mediates progression to secondary necrotic/pyroptotic cell death. *Nat Commun* **8**, 14128 (2017).

https://doi.org/10.1038/ncomms14128

6 Neel, D. V. *et al.* Gasdermin-E mediates mitochondrial damage in axons and neurodegeneration. *Neuron* **111**, 1222-1240.e1229 (2023). https://doi.org/10.1016/j.neuron.2023.02.019

7 An, H. *et al.* Tetraarsenic hexoxide enhances generation of mitochondrial ROS to promote pyroptosis by inducing the activation of caspase-3/GSDME in triple-negative breast cancer cells. *Cell death & disease* **12**, 159 (2021). https://doi.org/10.1038/s41419-021-03454-9

8 Zhang, D. *et al.* Mitochondria-Targeted Photodynamic Agents Drive Robust Pyroptosis by Caspase-3/GSDME Activation and Cardiolipin Externalization. *Angewandte Chemie (International ed. in English)*, e7359094 (2026). https://doi.org/10.1002/anie.7359094

9 Zhao, J. *et al.* GSDME-N-induced mitochondrial neurotoxicity in early neurodegeneration was suppressed by nicotine via enhancing autophagic flux. *Journal of translational medicine* **23**, 1202 (2025). https://doi.org/10.1186/s12967-025-07247-y

10 Senichkin, V. V., Streletskaia, A. Y., Gorbunova, A. S., Zhivotovsky, B. & Kopeina, G. S. Saga of Mcl-1: regulation from transcription to degradation. *Cell death and differentiation* **27**, 405-419 (2020). https://doi.org/10.1038/s41418-019-0486-3

11 Kotschy, A. *et al.* The MCL1 inhibitor S63845 is tolerable and effective in diverse cancer models. *Nature* **538**, 477-482 (2016). https://doi.org/10.1038/nature19830

12 Diepstraten, S. T. *et al.* Lymphoma cells lacking pro-apoptotic BAX are highly resistant to BH3-mimetics targeting pro-survival MCL-1 but retain sensitivity to conventional DNA-damaging drugs. *Cell Death & Differentiation* **30**, 1005-1017 (2023). https://doi.org/10.1038/s41418-023-01117-0

13 Hollville, E., Carroll, R. G., Cullen, S. P. & Martin, S. J. Bcl-2 family proteins participate in mitochondrial quality control by regulating Parkin/PINK1-dependent mitophagy. *Mol Cell* **55**, 451-466 (2014). https://doi.org/10.1016/j.molcel.2014.06.001

14 Chen, L. *et al.* Differential Targeting of Prosurvival Bcl-2 Proteins by Their BH3-Only Ligands Allows Complementary Apoptotic Function. *Molecular Cell* **17**, 393-403 (2005). https://doi.org/10.1016/j.molcel.2004.12.030

15 Haschka, M. D. *et al.* The NOXA–MCL1–BIM axis defines lifespan on extended mitotic arrest. *Nature Communications* **6** (2015). https://doi.org/10.1038/ncomms7891

16 Gama, V. *et al.* The E3 ligase PARC mediates the degradation of cytosolic cytochrome c to promote survival in neurons and cancer cells. *Science signaling* **7**, ra67 (2014). https://doi.org/10.1126/scisignal.2005309

17 Gama, V. *et al.* The E3 ligase PARC mediates the degradation of cytosolic cytochrome c to promote survival in neurons and cancer cells. *Science Signaling* **7**, ra67-ra67 (2014). https://doi.org/doi:10.1126/scisignal.2005309

18 Hollville, E. *et al.* Characterization of a Cul9-Parkin double knockout mouse model for Parkinson's disease. *Sci Rep* **10**, 16886 (2020). https://doi.org/10.1038/s41598-020-73854-y

19 Liang, J. R. *et al.* USP30 deubiquitylates mitochondrial Parkin substrates and restricts apoptotic cell death. *The EMBO Reports* **16**, 618-627 (2015). https://doi.org/10.15252/embr.201439820

20 Hung, Y.-C. *et al.* UQCRC1 engages cytochrome c for neuronal apoptotic cell death. *Cell Reports* **36** (2021). https://doi.org/10.1016/j.celrep.2021.109729

21 Obexer, P., Geiger, K., Ambros, P. F., Meister, B. & Ausserlechner, M. J. FKHRL1-mediated expression of Noxa and Bim induces apoptosis via the mitochondria in neuroblastoma cells. *Cell Death & Differentiation* **14**, 534-547 (2006). https://doi.org/10.1038/sj.cdd.4402017

22 Teran, E. *et al.* Structural basis and regulation of GSDME pore formation. *Nat Commun* **17**, 4148 (2026). https://doi.org/10.1038/s41467-026-70643-5

23 Rogers, C. *et al.* Gasdermin pores permeabilize mitochondria to augment caspase-3 activation during apoptosis and inflammasome activation. *Nat Commun* **10**, 1689 (2019). https://doi.org/10.1038/s41467-019-09397-2

24 van Delft, M. F. *et al.* The BH3 mimetic ABT-737 targets selective Bcl-2 proteins and efficiently induces apoptosis via Bak/Bax if Mcl-1 is neutralized. *Cancer cell* **10**, 389-399 (2006). https://doi.org/10.1016/j.ccr.2006.08.027

1st Revision - Editorial Decision **4th Sep 2026**

Dear Prof. Wang

Thank you for the submission of your revised manuscript to EMBO reports. We have now received the full set of referee reports that is copied below.

As you will see, both referees are very positive about the study and request only minor changes to clarify text and figures.

From the editorial side, there are also a few things that we need before we can proceed with the official acceptance of your study. Please provide a point-by-point response to the remaining referee concerns and to the editorial points listed below.

- The Disclosure Statement needs to be renamed to Disclosure and Competing Interests Statement and placed after the Acknowledgments.

- The references need to be alphabetical, not numerical; et al needs to be used after 10 author names.

- Please remove the separate Funding title and include the information on funding in the Acknowledgments.

- Please remove the Reagents and Tools table from the manuscript and upload it as individual word file. The typesetters will insert it during production.

- The Data Availability statement needs to be placed before the Acknowledgments.

- The Data Availability section may only refer to the datasets deposited in public repositories. Therefore, please remove the following text: "The GSEA analysis in this study was performed using these proteomic data to assess enrichment against the GO gene sets GO:0043069 and GO:2001234. These GO terms are part of the public Gene Ontology Resource (https://www.geneontology.org) and do not require any access codes. Other data are available from the corresponding author upon reasonable request."
If relevant, the information on the GO gene sets can/should be included in the relevant methods section.

- Materials and Methods should be Methods.

- BioRender should be acknowledged at the end of the Methods section in the following way:
Graphics:
(some of the... OR Figure #... OR synopsis) Graphics were created with BioRender.com.

- The Appendix file needs to be in PDF and clean (no track changes, highlighted parts, etc.). The title page should only state Appendix for "TITLE" and otherwise contain the table of contents.

- Author Checklist, line 73, refers to pre-registered protocols with a DOI or clinical trials. Please check again if this indeed applies to your study.

- Author Checklist, line 77, refers to protocols that were published as step-by-step protocol and can be cited (e.g., a publication in a methods-oriented journal or an online methods resource. Please check again whether this indeed applies to your study.

- Please provide the exact p values in the legends of figures 1B, C, F, G, I, J, L; 2B, H, K, N; 3F, H, J; 4H, I, K; 5C, 6D, F, I, K, N, P.
If the p-values are small ($p<0.0001$), these can be reported as inequalities.

- Please upload the source data as one ZIP folder per figure.

- Source Data: it is not necessary to supply the source data for the model figure shown in Figure 7 as source data.

- The source data supplied for immunofluorescent images, e.g., Figure 2J, 3B, matches exactly the image shown in the panel. Of course it should match, this is perfect, but often, a larger field of view was recorded and a part of this larger field of view is shown in the image panel. I.e., the original image obtained at the microscope shows more cells but was cropped to show a relevant part or chosen cells in the panel. If this is the case, it would be beneficial to show the original uncropped image obtained at the microscope to give an overview of what the original data looks like rather than a reproduction of the image shown in the panel. Source data is most valuable if it indeed shows the minimally processed original data. The source data for Figure 3B, e.g., are a reproduction of the images shown in the panel, including the arrows. Thank you for taking this into consideration.

- Your response to the referees contains unpublished data in form of reviewer figures. We publish the referee reports and your response to these alongside the paper as Peer Review File. Please let us know in case you want to remove any of the reviewer

figures. Please note that all data in the Peer Review File counts as published and that you would have to cite this document were your to re-use these in future publications.

- Finally, EMBO Reports papers are accompanied online by
A) a short (1-2 sentences) summary of the findings and their significance,
B) 2-3 bullet points highlighting key results and
C) a schematic summary figure that provides a sketch of the major findings (not a data image).
Please provide the summary figure as a separate file in PNG or JPG format at a size of 550x300 pixels (width x height). Please note that the size is rather small and that text needs to be readable at the final size. Please send us this information along with the revised manuscript.

I look forward to seeing a new revised version of your manuscript as soon as possible.

With kind regards,

Martina Rembold, PhD
Senior Editor
EMBO reports

==================

Referee #1:

The authors have adequately addressed all of the concerns I raised in my initial review, and I am satisfied with the revisions made to the manuscript. I have no further comments.

Referee #2:

The authors have made a substantial effort to address my previous concerns and have added an extensive set of new experiments. In particular, the matched comparison of PTEN-L knockout, wild-type, and PTEN-L-overexpressing cells shows a graded phenotype, clarifying that PTEN-L overexpression amplifies a protective function already exerted by endogenous PTEN-L. The matched time-course analyses, GSDME knockout and cleavage-resistant rescue experiments, BAX/BAK double-knockdown data, experiments in SH-SY5Y cells, and comparison between canonical PTEN and PTEN-L have further strengthened the physiological and mechanistic relevance of the study. My original concerns have therefore been adequately addressed.
Overall, the revised manuscript has been substantially improved, and I would support publication once the following minor issues have been addressed:

1. The manuscript still describes PTEN-L as acting through a "dual mechanism", involving both Parkin dephosphorylation and competitive disruption of the Parkin-MCL-1 complex. However, the co-immunoprecipitation experiments demonstrate association but do not establish direct competition. Moreover, the new experiments in Fig. 6E-F and Appendix Fig. S16B-C indicate that PTEN-L stabilizes MCL-1 indirectly through Parkin dephosphorylation and inactivation. In particular, the inability of PTEN-L to protect MCL-1 in the presence of Parkin-S65D argues that Parkin dephosphorylation is required for this effect. The statements that PTEN-L "competitively disrupts" the Parkin-MCL-1 complex and acts through an independent dual mechanism should therefore be removed or revised throughout the abstract, results and discussion. The wording currently used in Fig. 7, describing indirect stabilization of MCL-1 through Parkin dephosphorylation, appears more consistent with the data.

2. Reproducibility of Appendix Fig. S7A should be reported.

Re: EMBOR-2025-63377V2

Point-to-Point Responses to Editors' and Reviewers' Comments

Editor:

Thank you for the submission of your revised manuscript to EMBO reports. We have now received the full set of referee reports that is copied below. As you will see, both referees are very positive about the study and request only minor changes to clarify text and figures. From the editorial side, there are also a few things that we need before we can proceed with the official acceptance of your study. Please provide a point-by-point response to the remaining referee concerns and to the editorial points listed below.

Response: We are grateful to the Editor and Reviewer for their constructive comments and valuable suggestions on our work. We have thoroughly revised our manuscript in response, as detailed below.

1. The Disclosure Statement needs to be renamed to Disclosure and Competing Interests Statement and placed after the Acknowledgments.

Response: We thank the Editor for raising this important point. We have made this change precisely as specified. The statement is now titled "Disclosure and Competing Interests Statement" and appears after the Acknowledgments.

2. The references need to be alphabetical, not numerical; et al needs to be used after 10 author names.

Response: We thank the Editor for this important advice. We have reformatted all references throughout the manuscript to strictly follow EMBO Reports style. Specifically, we have changed the reference list from numerical order to alphabetical order, and have ensured that et al. is used after 10 author names, as required. These changes have been applied consistently to both the in-text citations and the final reference list. All corresponding modifications have been clearly highlighted in the revised manuscript for your verification.

3. Please remove the separate Funding title and include the information on funding in the Acknowledgments.

Response: We thank the Editor for this important advice. We have removed the previously separate Funding title and have now incorporated all funding-related information into the Acknowledgments section.

4. Please remove the Reagents and Tools table from the manuscript and upload it as

individual word file. The typesetters will insert it during production.

Response: We thank the Editor for this expert comment. We have removed the Reagents and Tools table from the main manuscript and have uploaded it as a separate Word file (named "Reagents and tools table.docx") along with the revised submission.

5. The Data Availability statement needs to be placed before the Acknowledgments.

Response: We sincerely thank the Editor for raising this important point. We have moved the Data Availability statement to the position immediately preceding the Acknowledgments section in the revised manuscript.

6. The Data Availability section may only refer to the datasets deposited in public repositories. Therefore, please remove the following text: "The GSEA analysis in this study was performed using these proteomic data to assess enrichment against the GO gene sets GO:0043069 and GO:2001234. These GO terms are part of the public Gene Ontology Resource (https://www.geneontology.org) and do not require any access codes. Other data are available from the corresponding author upon reasonable request." If relevant, the information on the GO gene sets can/should be included in the relevant methods section.

Response: Thanks again for the Editor's comment. We have removed the specified text from the Data Availability section entirely, as the section now refers exclusively to datasets deposited in public repositories.

7. Materials and Methods should be Methods.

Response: We sincerely appreciate the Editor for this constructive suggestion. We have changed the section heading from "Materials and Methods" to "Methods" in the revised manuscript.

8. BioRender should be acknowledged at the end of the Methods section in the following way:
Graphics:
(some of the... OR Figure #... OR synopsis) Graphics were created with BioRender.com.

Response: We thank the Editor for raising this important point. We have added the exact acknowledgment text "Figure 2L, Figure 7, and the synopsis graphics were created with BioRender.com." at the end of the Methods section in the revised manuscript.

9. The Appendix file needs to be in PDF and clean (no track changes, highlighted parts, etc.). The title page should only state Appendix for "TITLE" and otherwise contain the table of contents.

Response: We thank the Editor for this important advice. We have converted the Appendix file to PDF format and have removed all track changes, highlighted parts, and any other editing marks to ensure that the file is clean. We have also reformatted the title page so that it now states only Appendix for "TITLE" and otherwise contains the table of contents as specified.

10. Author Checklist, line 73, refers to pre-registered protocols with a DOI or clinical trials. Please check again if this indeed applies to your study.

Response: We thank the Editor for this expert comment. We have carried out the relevant actions and confirmed that this item is not applicable. Consequently, we have selected "not applicable" on the checklist.

11. Author Checklist, line 77, refers to protocols that were published as step-by-step protocol and can be cited (e.g., a publication in a methods-oriented journal or an online methods resource. Please check again whether this indeed applies to your study.

Response: We sincerely thank the Editor for raising this important point. We have re-examined this item and confirmed that it does not apply to our study, as we did not have any step-by-step protocols published in a methods-oriented journal or online methods resource that could be cited. Accordingly, we have changed the response to "not applicable" in the revised Author Checklist.

12. Please provide the exact p values in the legends of figures 1B, C, F, G, I, J, L; 2B, H, K, N; 3F, H, J; 4H, I, K; 5C, 6D, F, I, K, N, P. If the p-values are small ($p<0.0001$), these can be reported as inequalities.

Response: Thanks again for the Editor's comment. We have revised the legends of figures 1B, C, F, G, I, J, L; 2B, H, K, N; 3F, H, J; 4H, I, K; 5C, 6D, F, I, K, N, P to include the exact p values. For p-values smaller than 0.0001, we have reported them as inequalities ($P < 0.0001$) as permitted.

13. Please upload the source data as one ZIP folder per figure.

Response: We thank the Editor for raising this important point. We have now packaged the source data for each figure into individual ZIP folders, with each folder named according to its corresponding figure number (e.g., Source Data Figure 1, Source Data Figure 2, etc.). All ZIP folders have been uploaded as separate files along with the revised submission.

14. Source Data: it is not necessary to supply the source data for the model figure shown in Figure 7 as source data.

Response: We thank the Editor for this important advice. We have removed the source data file that was previously submitted for Figure 7 and have confirmed that no source data is required for this model figure.

15. The source data supplied for immunofluorescent images, e.g., Figure 2J, 3B, matches exactly the image shown in the panel. Of course it should match, this is perfect, but often, a larger field of view was recorded and a part of this larger field of view is shown in the image panel. I.e., the original image obtained at the microscope shows more cells but was cropped to show a relevant part or chosen cells in the panel. If this is the case, it would be beneficial to show the original uncropped image obtained at the microscope to give an overview of what the original data looks like rather than a reproduction of the image shown in the panel. Source data is most valuable if it indeed shows the minimally processed original data. The source data for Figure 3B, e.g., are a reproduction of the images shown in the panel, including the arrows. Thank you for taking this into consideration.

Response: We thank the Editor for this expert comment. We fully appreciate your concern regarding the source data and the importance of providing the original uncropped field of view to reflect the raw data. In accordance with your request, we have thoroughly revised the source data for all relevant immunofluorescent images as follows.

For Figures 1K, 2J, 3B, and 3I, we have now included the original uncropped images directly exported from the microscope without any modification or processing, which show the full field of view prior to any cropping. To facilitate clear navigation and verification, we have organized the source data files into two separate subfolders within each figure's ZIP folder: a subfolder named "Original data" containing the unprocessed raw uncropped images, and a subfolder named "Show data" containing the cropped images that correspond exactly to what is presented in the main figure panels. This structure allows the editor to readily compare the original data with the final presented images.

For Figures 3E, 4J, and 6C, we have kept the source data unchanged, as these images were originally acquired and presented without any cropping; the field of view shown in the main figure panels already represents the full original acquisition field.

16. Your response to the referees contains unpublished data in form of reviewer figures. We publish the referee reports and your response to these alongside the paper as Peer Review File. Please let us know in case you want to remove any of the reviewer figures. Please note that all data in the Peer Review File counts as published and that you would have to cite this document were your to re-use these in future publications.

Response: Thanks again for the Editor's comment. We do not intend to remove any of the reviewer figures from the Review Process File. We are amenable to all figures

included in our response to the referees being published as part of the Peer Review File.

17. Finally, EMBO Reports papers are accompanied online by
A) a short (1-2 sentences) summary of the findings and their significance,
B) 2-3 bullet points highlighting key results and
C) a schematic summary figure that provides a sketch of the major findings (not a data image).
Please provide the summary figure as a separate file in PNG or JPG format at a size of 550x300 pixels (width x height). Please note that the size is rather small and that text needs to be readable at the final size. Please send us this information along with the revised manuscript.

Response: We thank the Editor for raising this important point. We have prepared all the three requisite items, as detailed below. For the schematic summary figure, we have created a figure that provides a sketch of the major findings and have uploaded it as a separate PNG file at the specified size of 550x300 pixels (width x height), with text kept readable at the final size. For the short summary of the findings, significance, and 3 bullet points highlighting the key results, the detailed text information is as follows:

Summary
Mitochondrial damage activates the Parkin–MCL-1–BAX/BAK–GSDME axis to trigger pyroptosis. PTEN-L acts as a crucial regulator of this pathway by directly dephosphorylating Parkin and inhibiting its E3 ligase activity, thereby maintaining the level of MCL-1 and concurrently suppressing both mitophagy and pyroptosis. This discovery reveals a phosphorylation switch that links organelle quality control to inflammatory cell death.

Highlights
1) Mitochondrial depolarization activates the PINK1–Parkin axis to drive GSDME-dependent pyroptosis through Parkin-mediated ubiquitination and degradation of MCL-1, which unleashes the BAX/BAK–caspase-3 cascade.
2) PTEN-L functions as a critical brake on this pathway by directly dephosphorylating Parkin and restraining its E3 ligase activity, thereby preserving MCL-1 and suppressing both mitophagy and pyroptosis.
3) This study identifies a phosphorylation-dependent regulatory switch centered on Parkin, which couples organelle quality control to inflammatory cell death and expands the functional repertoire of the PINK1 – Parkin axis beyond mitophagy.

Referee #1:

The authors have adequately addressed all of the concerns I raised in my initial review, and I am satisfied with the revisions made to the manuscript. I have no further comments.

Response: We are happy that you are satisfied with the revised manuscript.

Referee #2:

The authors have made a substantial effort to address my previous concerns and have added an extensive set of new experiments. In particular, the matched comparison of PTEN-L knockout, wild-type, and PTEN-L-overexpressing cells shows a graded phenotype, clarifying that PTEN-L overexpression amplifies a protective function already exerted by endogenous PTEN-L. The matched time-course analyses, GSDME knockout and cleavage-resistant rescue experiments, BAX/BAK double-knockdown data, experiments in SH-SY5Y cells, and comparison between canonical PTEN and PTEN-L have further strengthened the physiological and mechanistic relevance of the study. My original concerns have therefore been adequately addressed.
Overall, the revised manuscript has been substantially improved, and I would support publication once the following minor issues have been addressed:

Response: We are grateful to the Reviewer for your nice summary, positive evaluation, and constructive suggestions to our work. We have thoroughly revised our manuscript in response, as detailed below.

1. The manuscript still describes PTEN-L as acting through a "dual mechanism", involving both Parkin dephosphorylation and competitive disruption of the Parkin-MCL-1 complex. However, the co-immunoprecipitation experiments demonstrate association but do not establish direct competition. Moreover, the new experiments in Fig. 6E-F and Appendix Fig. S16B-C indicate that PTEN-L stabilizes MCL-1 indirectly through Parkin dephosphorylation and inactivation. In particular, the inability of PTEN-L to protect MCL-1 in the presence of Parkin-S65D argues that Parkin dephosphorylation is required for this effect. The statements that PTEN-L "competitively disrupts" the Parkin-MCL-1 complex and acts through an independent dual mechanism should therefore be removed or revised throughout the abstract, results and discussion. The wording currently used in Fig. 7, describing indirect stabilization of MCL-1 through Parkin dephosphorylation, appears more consistent with the data.

Response: We sincerely appreciate the Reviewer for this constructive suggestion. We fully agree with the reviewer's assessment. Accordingly, we have carefully revised the manuscript to remove all statements describing PTEN-L as acting through a “dual mechanism” or “competitively disrupting” the Parkin-MCL-1 complex, as these are not directly supported by our co-immunoprecipitation data. The revised text now consistently describes PTEN-L function through dephosphorylation and inactivation of Parkin, which indirectly stabilizes MCL-1. These changes have been made in the abstract (line 33), introduction (line 118), results (line 432), and discussion (line 549). We believe this revision aligns the text with the experimental evidence and with the model presented in Figure 7.

2. Reproducibility of Appendix Fig. S7A should be reported.

Response: We thank the Reviewer for this important question. We have added a statement regarding the reproducibility of Appendix Fig. S7A directly in the figure legend. The legend now specifies: “Microscopy images in (**A**) are representative of three independent biological replicates.”

Prof. Liming Wang
Hunan University
School of biomedical sciences
Changsha, Hunan
China

Dear Prof. Wang,

I am very pleased to accept your manuscript for publication in the next available issue of EMBO reports. Thank you for your contribution to our journal.

Your manuscript will be processed for publication by EMBO Press. It will be copy edited and you will receive page proofs prior to publication. Please note that you will be contacted by Springer Nature Author Services to complete licensing and payment information.

You may qualify for financial assistance for your publication charges - either via a Springer Nature fully open access agreement or an EMBO initiative. Check your eligibility: https://link.springer.com/journal/44319/how-to-publish-with-us

Should you be planning a Press Release on your article, please notify the production team via this form in order to coordinate publication and release dates: https://forms.cloud.microsoft/e/eH4A8JVP6g

If you have any questions, please do not hesitate to contact the Editorial Office. Thank you for your contribution to EMBO Reports.

Yours sincerely,

Martina Rembold, PhD
Senior Editor
EMBO reports

>>> Please note that it is EMBO Reports policy for the transcript of the editorial process (containing referee reports and your response letter) to be published as an online supplement to each paper. If you do NOT want this, you will need to inform the Editorial Office via email immediately. More information is available here: https://link.springer.com/partners/embo-press/editorial-policies#Peer%20review

EMBO Press Author Checklist

Corresponding Author Name: Liming Wang	
Journal Submitted to: EMBO Reports	
Manuscript Number: EMBOR-2025-63377	

USEFUL LINKS FOR COMPLETING THIS FORM
The EMBO Journal - Author Guidelines
EMBO Reports - Author Guidelines
Molecular Systems Biology - Author Guidelines
EMBO Molecular Medicine - Author Guidelines

Reporting Checklist for Life Science Articles (updated January
This checklist is adapted from Materials Design Analysis Reporting (MDAR) Checklist for Authors. MDAR establishes a minimum set of requirements in transparent reporting in the life sciences (see Statement of Task: 10.31222/osf.io/9sm4x). Please follow the journal's guidelines in preparing your
Please note that a copy of this checklist will be published alongside your article.

Abridged guidelines for figures
1. Data
The data shown in figures should satisfy the following conditions:

- the data were obtained and processed according to the field's best practice and are presented to reflect the results of the experiments in an accurate and unbiased manner.
- ideally, figure panels should include only measurements that are directly comparable to each other and obtained with the same assay.
- plots include clearly labeled error bars for independent experiments and sample sizes. Unless justified, error bars should not be shown for technical
- if n<5, the individual data points from each experiment should be plotted. Any statistical test employed should be justified.
- Source Data should be included to report the data underlying figures according to the guidelines set out in the authorship guidelines on Data

2. Captions
Each figure caption should contain the following information, for each panel where they are relevant:

- a specification of the experimental system investigated (eg cell line, species name).
- the assay(s) and method(s) used to carry out the reported observations and measurements.
- an explicit mention of the biological and chemical entity(ies) that are being measured.
- an explicit mention of the biological and chemical entity(ies) that are altered/varied/perturbed in a controlled manner.
- the exact sample size (n) for each experimental group/condition, given as a number, not a range;
- a description of the sample collection allowing the reader to understand whether the samples represent technical or biological replicates (including how many animals, litters, cultures, etc.).
- a statement of how many times the experiment shown was independently replicated in the laboratory.
- definitions of statistical methods and measures:
  - common tests, such as t-test (please specify whether paired vs. unpaired), simple χ2 tests, Wilcoxon and Mann-Whitney tests, can be unambiguously identified by name only, but more complex techniques should be described in the methods section;
  - are tests one-sided or two-sided?
  - are there adjustments for multiple comparisons?
  - exact statistical test results, e.g., P values = x but not P values < x;
  - definition of 'center values' as median or average;
  - definition of error bars as s.d. or s.e.m.

Please complete ALL of the questions below.
Select "Not Applicable" only when the requested information is not relevant for your study.

Materials

Newly Created Materials	Information included in the manuscript?	In which section is the information available? (Reagents and Tools Table, Materials and Methods, Figures, Data Availability Section)
New materials and reagents need to be available; do any restrictions apply?	Not Applicable	

Antibodies	Information included in the manuscript?	In which section is the information available? (Reagents and Tools Table, Materials and Methods, Figures, Data Availability Section)
For **antibodies** provide the following information:<br>- Commercial antibodies: RRID (if possible) or supplier name, catalogue number and or/clone number<br>- Non-commercial: RRID or citation	Yes	Reagents and Tools Table

DNA and RNA sequences	Information included in the manuscript?	In which section is the information available? (Reagents and Tools Table, Materials and Methods, Figures, Data Availability Section)
**Short novel DNA or RNA including primers, probes**: provide the sequences.	Yes	Reagents and Tools Table

Cell materials	Information included in the manuscript?	In which section is the information available? (Reagents and Tools Table, Materials and Methods, Figures, Data Availability Section)
**Cell lines:** Provide species information, strain. Provide accession number in repository **OR** supplier name, catalog number, clone number, and/**OR** RRID.	Yes	Reagents and Tools Table
**Primary cultures:** Provide species, strain, sex of origin, genetic modification status.	Not Applicable	
Report if the cell lines were recently **authenticated** (e.g., by STR profiling) and tested for mycoplasma contamination.	Yes	Methods

Experimental animals	Information included in the manuscript?	In which section is the information available? (Reagents and Tools Table, Materials and Methods, Figures, Data Availability Section)
**Laboratory animals or Model organisms:** Provide species, strain, sex, age, genetic modification status. Provide accession number in repository **OR** supplier name, catalog number, clone number, **OR** RRID.	Not Applicable	
**Animal observed in or captured from the field:** Provide species, sex, and age where possible.	Not Applicable	
Please detail **housing and husbandry conditions**.	Not Applicable	

Plants and microbes	Information included in the manuscript?	In which section is the information available? (Reagents and Tools Table, Materials and Methods, Figures, Data Availability Section)
**Plants:** provide species and strain, ecotype and cultivar where relevant, unique accession number if available, and source (including location for collected wild specimens).	Not Applicable	
**Microbes:** provide species and strain, unique accession number if available, and source.	Yes	methods

Human research participants	Information included in the manuscript?	In which section is the information available? (Reagents and Tools Table, Materials and Methods, Figures, Data Availability Section)
If collected and within the bounds of privacy constraints report on age, sex and gender or ethnicity for all study participants.	Not Applicable	

Core facilities	Information included in the manuscript?	In which section is the information available? (Reagents and Tools Table, Materials and Methods, Figures, Data Availability Section)
If your work benefited from core facilities, was their service mentioned in the acknowledgments section?	Yes	Acknowledgments

Design

Study protocol	Information included in the manuscript?	In which section is the information available? (Reagents and Tools Table, Materials and Methods, Figures, Data Availability Section)
If study protocol has been **pre-registered, provide DOI in the manuscript**. For clinical trials, provide the trial registration number **OR** cite DOI.	Not Applicable	
Report the **clinical trial registration number** (at ClinicalTrials.gov or equivalent), where applicable.	Not Applicable	

Laboratory protocol	Information included in the manuscript?	In which section is the information available? (Reagents and Tools Table, Materials and Methods, Figures, Data Availability Section)
Provide DOI OR other citation details if **external detailed step-by-step protocols** are available.	Not Applicable	

Experimental study design and statistics	Information included in the manuscript?	In which section is the information available? (Reagents and Tools Table, Materials and Methods, Figures, Data Availability Section)
Include a statement about **sample size** estimate even if no statistical methods were used.	Yes	Methods and Figure legends
Were any steps taken to minimize the effects of subjective bias when allocating animals/samples to treatment (e.g. **randomization procedure**)? If yes, have they been described?	Not Applicable	
Include a statement about **blinding** even if no blinding was done.	Not Applicable	
Describe **inclusion/exclusion criteria** if samples or animals were excluded from the analysis. Were the criteria pre-established?<br><br>If sample or data points were omitted from analysis, report if this was due to attrition or intentional exclusion and provide justification.	Not Applicable	
For every figure, are **statistical tests** justified as appropriate? Do the data meet the assumptions of the tests (e.g., normal distribution)? Describe any methods used to assess it. Is there an estimate of variation within each group of data? Is the variance similar between the groups that are being statistically compared?	Yes	Methods and Figure legends

Sample definition and in-laboratory replication	Information included in the manuscript?	In which section is the information available? (Reagents and Tools Table, Materials and Methods, Figures, Data Availability Section)
In the figure legends: state number of times the experiment was **replicated** in laboratory.	Yes	Figure legends
In the figure legends: define whether data describe **technical or biological replicates**.	Yes	Figure legends

Ethics

Ethics	Information included in the manuscript?	In which section is the information available? (Reagents and Tools Table, Materials and Methods, Figures, Data Availability Section)
Studies involving **human participants**: State details of **authority granting ethics approval** (IRB or equivalent committee(s), provide reference number for approval.	Not Applicable	
Studies involving **human participants**: Include a statement confirming that **informed consent** was obtained from all subjects and that the experiments conformed to the principles set out in the WMA Declaration of Helsinki and the Department of Health and Human Services Belmont Report.	Not Applicable	
Studies involving **human participants:** For publication of **patient photos**, include a statement confirming that consent to publish was obtained.	Not Applicable	
Studies involving experimental **animals**: State details of **authority granting ethics approval** (IRB or equivalent committee(s), provide reference number for approval. Include a statement of compliance with ethical regulations.	Not Applicable	
Studies involving **specimen and field samples:** State if relevant **permits** obtained, provide details of authority approving study; if none were required, explain why.	Not Applicable	

Dual Use Research of Concern (DURC)	Information included in the manuscript?	In which section is the information available? (Reagents and Tools Table, Materials and Methods, Figures, Data Availability Section)
Could your study fall under dual use research restrictions? Please check biosecurity documents and list of **select agents and toxins** (CDC): https://www.selectagents.gov/sat/list.htm	Not Applicable	
If you used a select agent, is the security level of the lab appropriate and reported in the manuscript?	Not Applicable	
If a study is subject to dual use research of concern regulations, is the name of the **authority granting approval and reference number** for the regulatory approval provided in the manuscript?	Not Applicable	

Reporting

The MDAR framework recommends adoption of discipline-specific guidelines, established and endorsed through community initiatives. Journals have their own policy about requiring specific guidelines and recommendations to complement MDAR.

Adherence to community standards	Information included in the manuscript?	In which section is the information available? (Reagents and Tools Table, Materials and Methods, Figures, Data Availability Section)
State if relevant guidelines or checklists (e.g., **ICMJE, MIBBI, ARRIVE, PRISMA**) have been followed or provided.	Not Applicable	
For **tumor marker prognostic studies,** we recommend that you follow the **REMARK** reporting guidelines (see link list at top right). See author guidelines, under 'Reporting Guidelines'. Please confirm you have followed these guidelines.	Not Applicable	
For **phase II and III randomized controlled trials**, please refer to the **CONSORT** flow diagram (see link list at top right) and submit the CONSORT checklist (see link list at top right) with your submission. See author guidelines, under 'Reporting Guidelines'. Please confirm you have submitted this list.	Not Applicable	

Data Availability

Data availability	Information included in the manuscript?	In which section is the information available? (Reagents and Tools Table, Materials and Methods, Figures, Data Availability Section)
Have **primary datasets** been deposited according to the journal's guidelines (see 'Data Deposition' section) and the respective accession numbers provided in the Data Availability Section?	Yes	Data Availability Section
Were **human clinical and genomic datasets** deposited in a public access-controlled repository in accordance to ethical obligations to the patients and to the applicable consent agreement?	Not Applicable	
Are **computational models** that are central and integral to a study available without restrictions in a machine-readable form? Were the relevant accession numbers or links provided?	Not Applicable	
If publicly available data were reused, provide the respective **data citations in the reference list**.	Not Applicable	
