# Supplementary material for: PTEN-L inhibits mitophagy and GSDME-dependent pyroptosis via Parkin dephosphorylation
Source: EMBO Rep. 2026 Sep 28;27(21):6166–90. doi: 10.1038/s44319-026-00957-4 (PMC13743760; doi:10.1038/s44319-026-00957-4)
Supplement: Supplementary file 2 [file 44319_2026_957_MOESM2_ESM.pdf]

**Appendix**

# PTEN-L inhibits mitophagy and GSDME-dependent pyroptosis via Parkin dephosphorylation

**Table of contents**

Appendix Figure S1………………..page 3
Appendix Figure S2………………..page 5
Appendix Figure S3………………..page 8
Appendix Figure S4………………..page 11
Appendix Figure S5………………..page 14
Appendix Figure S6………………..page 17
Appendix Figure S7………………..page 19
Appendix Figure S8………………..page 22
Appendix Figure S9………………..page 25
Appendix Figure S10………………page 27
Appendix Figure S11………………page 29
Appendix Figure S12………………page 31
Appendix Figure S13………………page 33
Appendix Figure S14………………page 36
Appendix Figure S15………………page 38
Appendix Figure S16………………page 41
Appendix Figure S17………………page 44

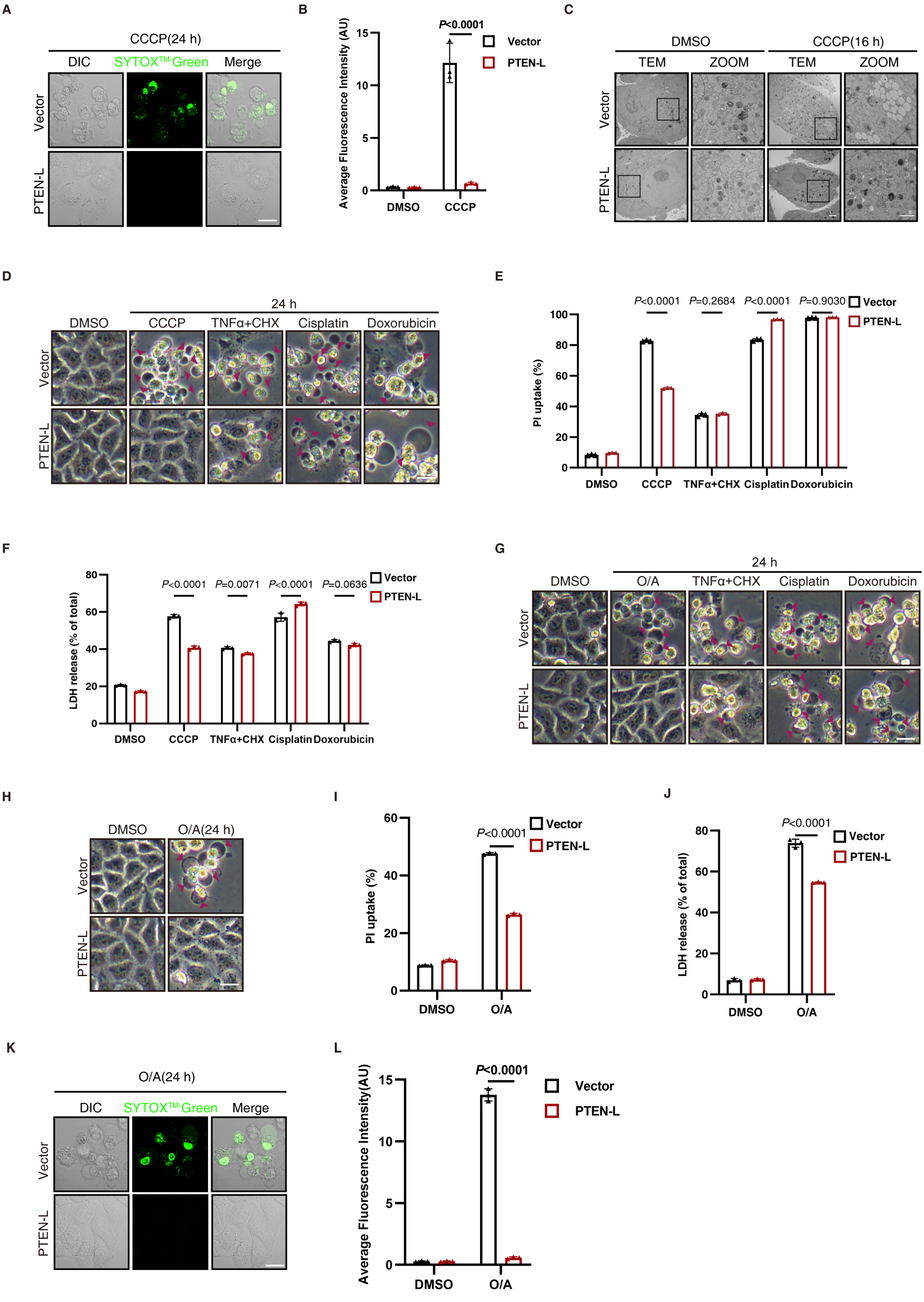
A
CCCP(24 h)
DIC
SYTOX™ Green
Merge
Vector
PTEN-L
B
P<0.0001
Average Fluorescence Intensity (AU)
15
10
5
0
DMSO
CCCP
Vector
PTEN-L
C
DMSO
CCCP(16 h)
TEM
ZOOM
TEM
ZOOM
Vector
PTEN-L
D
24 h
DMSO
CCCP
TNFα+CHX
Cisplatin
Doxorubicin
Vector
PTEN-L
E
P<0.0001
P=0.2684
P<0.0001
P=0.9030
Vector
PTEN-L
PI uptake (%)
100
80
60
40
20
0
DMSO
CCCP
TNFα+CHX
Cisplatin
Doxorubicin
F
LDH release (% of total)
80
60
40
20
0
P<0.0001
P=0.0071
P<0.0001
P=0.0636
Vector
PTEN-L
DMSO
CCCP
TNFα+CHX
Cisplatin
Doxorubicin
G
24 h
DMSO
O/A
TNFα+CHX
Cisplatin
Doxorubicin
Vector
PTEN-L
H
DMSO
O/A(24 h)
Vector
PTEN-L
I
PI uptake (%)
60
40
20
0
P<0.0001
Vector
PTEN-L
DMSO
O/A
J
LDH release (% of total)
80
60
40
20
0
P<0.0001
Vector
PTEN-L
DMSO
O/A
K
O/A(24 h)
DIC
SYTOX™ Green
Merge
Vector
PTEN-L
L
P<0.0001
Average Fluorescence Intensity(AU)
15
10
5
0
Vector
PTEN-L
DMSO
O/A

**Appendix Figure S1. PTEN-L suppress cell death following mitochondrial depolarization.** (**A** and **B**) Representative images and average fluorescence intensity (AU) of SYTOX Green staining in HeLa YFP-Parkin Vector/PTEN-L cells following treatment with 20 μM CCCP for 24 h. Scale bars, 20 μm. (**C**) Representative and zoom high-resolution transmission electron microscopy (TEM) images of HeLa YFP-Parkin Vector/PTEN-L cells following treatment with 20 μM CCCP for 16 h. Scale bars, 2 μm. (**D** to **F**) Representative microscopy images, PI uptake and LDH release assay of HeLa YFP-Parkin Vector/PTEN-L cells following treatment with 20 μM CCCP, 20 ng/mL TNFα plus 10 ug/mL CHX, 20 μg/mL Cisplatin or 1 μM Doxorubicin for 24 h. Scale bar, 25 μm. (**G**) Representative microscopy images for HeLa YFP-Parkin Vector/PTEN-L cells following treatment with 20 nM/ 200 nM O/A, 20 ng/mL TNFα plus 10 ug/mL CHX, 20 μg/mL Cisplatin or 1 μM Doxorubicin for 24 h. Scale bar, 25 μm. (**H** to **J**) Representative microscopy images, PI uptake and LDH release assay of HeLa YFP-Parkin Vector/PTEN-L cells following treatment with 20 nM oligomycin plus 200 nM antimycin A (O/A) for 24 h. Scale bar, 25 μm. (**K** and **L**) Representative images and average fluorescence intensity (AU) of SYTOX Green staining in HeLa YFP-Parkin Vector/PTEN-L cells following treatment with 20 nM/ 200 nM O/A for 24 h. Scale bar, 20 μm. Red arrows in (**D**), (**G**), and (**H**) indicate pyroptotic cells. Data in (**B**), (**E**), (**F**), (**I**), (**J**), and (**L**) are presented as mean ± SD from three independent biological replicates. Statistical significance was assessed by two-way ANOVA.

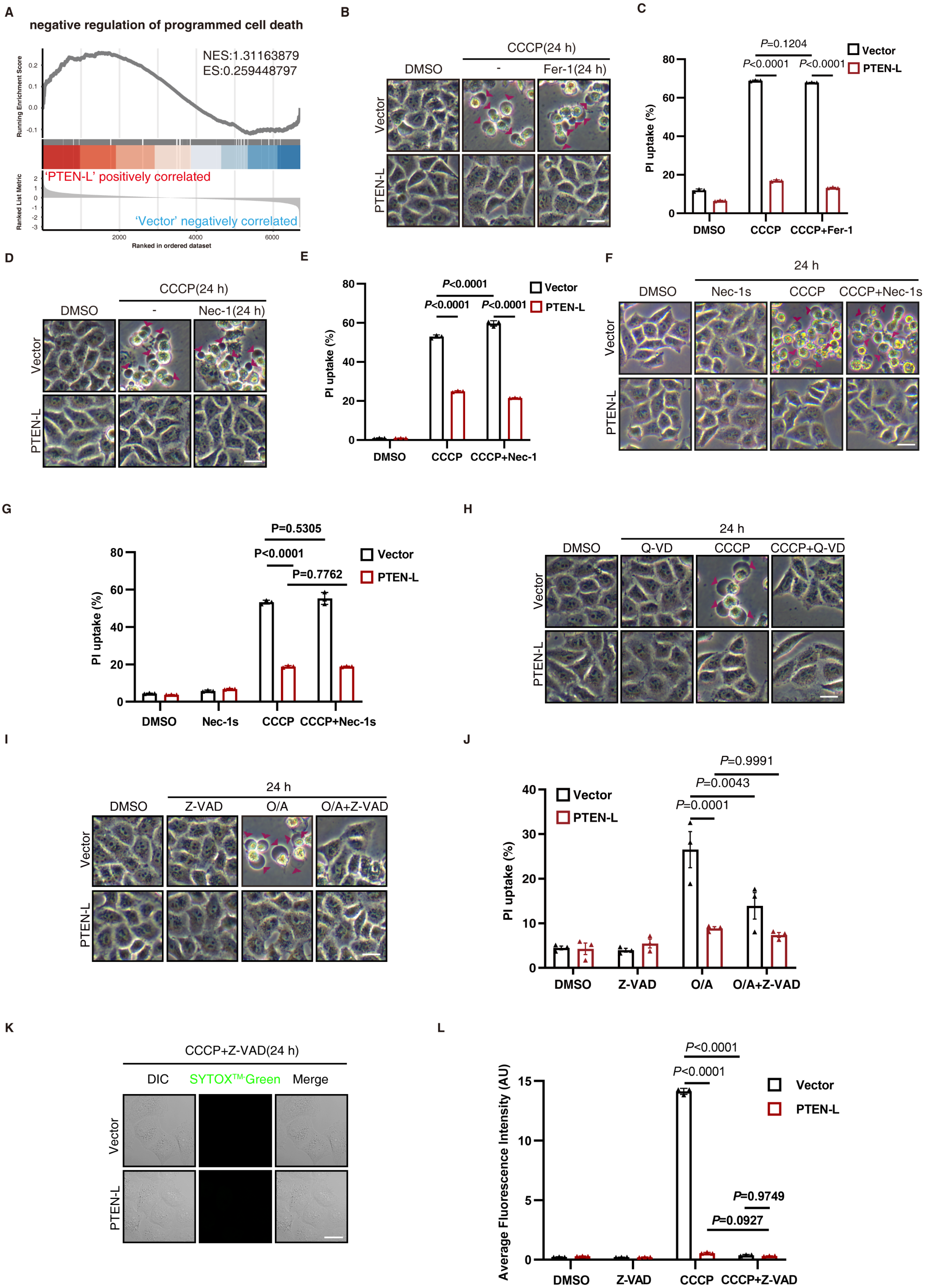
A
negative regulation of programmed cell death
NES:1.31163879
ES:0.259448797
Running Enrichment Score
'PTEN-L' positively correlated
'Vector' negatively correlated
Ranked List Metric
Ranked in ordered dataset
B
CCCP(24 h)
DMSO
-
Fer-1(24 h)
Vector
PTEN-L
C
P=0.1204
P<0.0001
P<0.0001
Vector
PTEN-L
PI uptake (%)
DMSO
CCCP
CCCP+Fer-1
D
CCCP(24 h)
DMSO
-
Nec-1(24 h)
Vector
PTEN-L
E
P<0.0001
P<0.0001
P<0.0001
Vector
PTEN-L
PI uptake (%)
DMSO
CCCP
CCCP+Nec-1
F
24 h
DMSO
Nec-1s
CCCP
CCCP+Nec-1s
Vector
PTEN-L
G
P=0.5305
P<0.0001
P=0.7762
Vector
PTEN-L
PI uptake (%)
DMSO
Nec-1s
CCCP
CCCP+Nec-1s
H
24 h
DMSO
Q-VD
CCCP
CCCP+Q-VD
Vector
PTEN-L
I
24 h
DMSO
Z-VAD
O/A
O/A+Z-VAD
Vector
PTEN-L
J
P=0.9991
P=0.0043
P=0.0001
Vector
PTEN-L
PI uptake (%)
DMSO
Z-VAD
O/A
O/A+Z-VAD
K
CCCP+Z-VAD(24 h)
DIC
SYTOX™ Green
Merge
Vector
PTEN-L
L
P<0.0001
P<0.0001
Vector
PTEN-L
Average Fluorescence Intensity (AU)
P=0.9749
P=0.0927
DMSO
Z-VAD
CCCP
CCCP+Z-VAD

**Appendix Figure S2. Caspase inhibitors suppress cell death following mitochondrial depolarization.** (**A**) Gene Set Enrichment Analysis (GSEA) of proteomics data of Hela YFP-Parkin Vector/PTEN-L cell lysates following treatment with 20 μM CCCP for 18 h. The data was acquired from the Gene Ontology (GO) database. ES is an abbreviation of Enrichment Score, and NES represents Normalized Enrichment Score. (**B** and **C**) Representative microscopy images and PI uptake assay of HeLa YFP-Parkin Vector/PTEN-L cells following treatment with 20 μM CCCP in combination with or without 1 μM ferrostatin-1 (Fer-1) for 24 h. Scale bar, 25 μm. (**D** and **E**) Representative microscopy images and PI uptake assay of HeLa YFP-Parkin Vector/PTEN-L cells following treatment with 20 μM CCCP in combination with or without 20 μM necrostatin-1 (Nec-1) for 24 h. Scale bar, 25 μm. (**F** and **G**) Representative microscopy images and PI uptake assay of HeLa YFP-Parkin Vector/PTEN-L cells following treatment with 20 μM CCCP in combination with or without 10 μM Necrostatin 2 racemate (Nec-1s) for 24 h. Scale bar, 25 μm. (**H**) Representative microscopy images of HeLa YFP-Parkin Vector/PTEN-L cells following treatment with 20 μM CCCP in combination with or without 40 μM Q-VD-OPh (Q-VD) for 24 h. Scale bar, 25 μm. (**I** and **J**) Representative microscopy images and PI uptake assay of HeLa YFP-Parkin Vector/PTEN-L cells following treatment with 20 nM/200 nM O/A in combination with or without 40 μM Z-VAD for 24 h. Scale bar, 25 μm. (**K** and **L**) Representative images and average fluorescence intensity (AU) of SYTOX Green staining in HeLa YFP-Parkin Vector/PTEN-L cells following treatment with 20 μM CCCP in combination with 40 μM Z-VAD for 24 h.

Scale bars, 20 μm. Red arrows in (**B**), (**D**), (**F**), (**H**), and (**I**) indicate pyroptotic cells. Data in (**C**), (**E**), (**G**), (**J**), and (**L**) are presented as mean ± SD from three independent biological replicates. Statistical significance was assessed by two-way ANOVA.

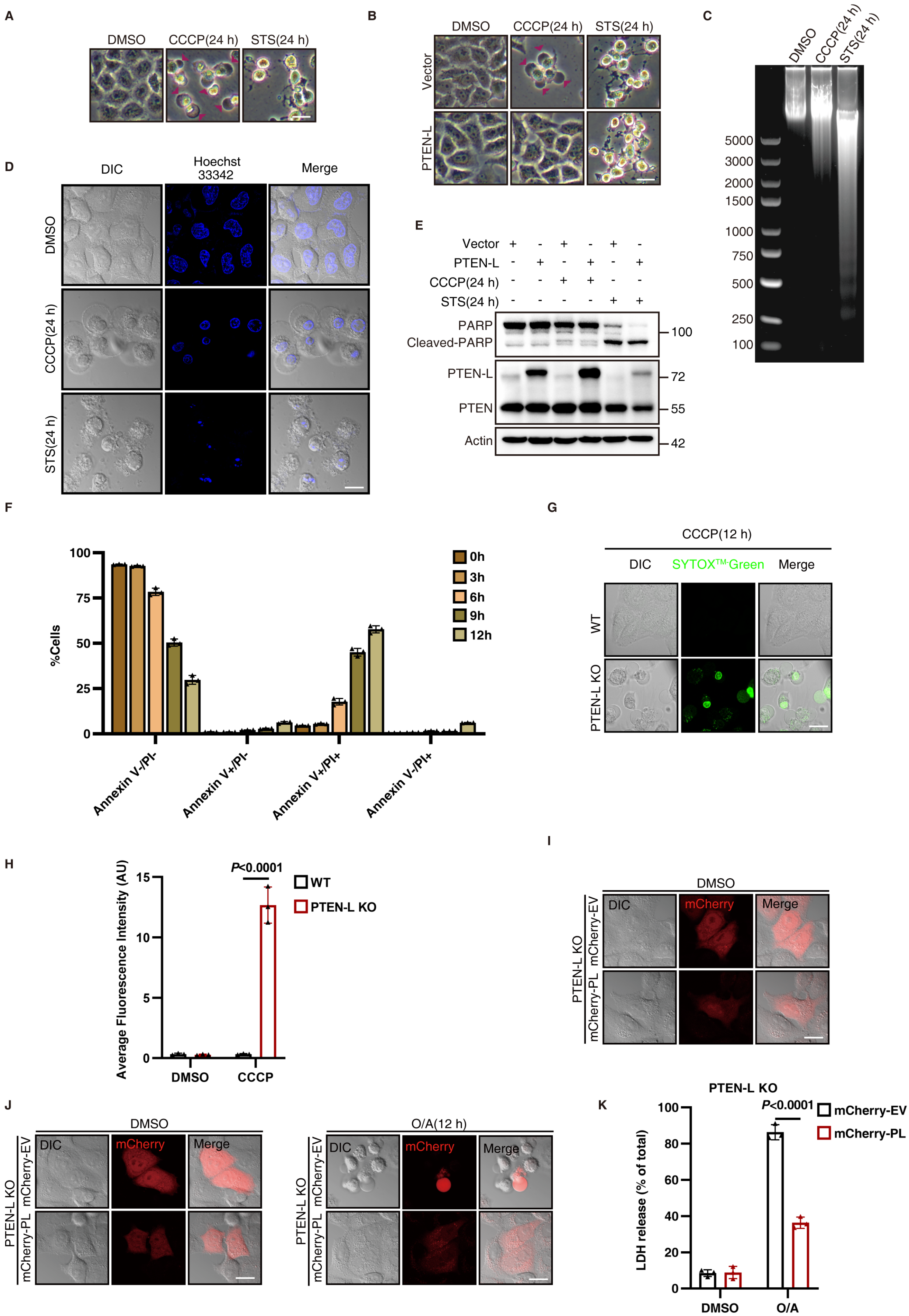

A
DMSO
CCCP(24 h)
STS(24 h)
B
DMSO
CCCP(24 h)
STS(24 h)
Vector
PTEN-L
C
DMSO
CCCP(24 h)
STS(24 h)
5000
3000
2000
1500
1000
750
500
250
100
D
DIC
Hoechst 33342
Merge
DMSO
CCCP(24 h)
STS(24 h)
E
Vector + - + - + -
PTEN-L - + - + - +
CCCP(24 h) - - + + - -
STS(24 h) - - - - + +
PARP
Cleaved-PARP
PTEN-L
PTEN
Actin
100
72
55
42
F
%Cells
100
75
50
25
0
0h
3h
6h
9h
12h
Annexin V-/PI-
Annexin V+/PI-
Annexin V+/PI+
Annexin V-/PI+
G
CCCP(12 h)
DIC
SYTOX™ Green
Merge
WT
PTEN-L KO
H
Average Fluorescence Intensity (AU)
P<0.0001
15
10
5
0
DMSO
CCCP
WT
PTEN-L KO
I
DMSO
DIC
mCherry
Merge
PTEN-L KO mCherry-EV
PTEN-L KO mCherry-PL
J
DMSO
O/A(12 h)
DIC
mCherry
Merge
PTEN-L KO mCherry-EV
PTEN-L KO mCherry-PL
K
PTEN-L KO
LDH release (% of total)
P<0.0001
100
80
60
40
20
0
DMSO
O/A
mCherry-EV
mCherry-PL

**Appendix Figure S3. Treatment with CCCP for 24 h does not elicit discernible apoptotic characteristics and Rescue of PTEN-L expression in PTEN-L KO cells inhibits cell death.** (**A**) Representative microscopy images of HeLa YFP-Parkin cells following treatment with 20 μM CCCP or 5 μM Staurosporine (STS) for 24 h. STS was used as a positive control. Scale bar, 25 μm. (**B**) Representative microscopy images of HeLa YFP-Parkin Vector/PTEN-L cells following treatment with 20 μM CCCP or 5 μM Staurosporine (STS) for 24 h. Scale bar, 25 μm. (**C**) DNA ladder formation of HeLa YFP-Parkin cells treated with 20 μM CCCP or 5 μM STS for 24 h. (**D**)The detection of nuclear fragmentation using Hoechst 33342 for HeLa YFP-Parkin cells following treatment with 20 μM CCCP or 5 μM STS for 24 h. Scale bar, 20 μm. (**E**) Immunoblotting analysis of HeLa YFP-Parkin Vector/PTEN-L cells following treatment with 20 μM CCCP or 5 μM Staurosporine (STS) for 24 h. (**F**) Annexin V/PI staining of HeLa HA-Parkin cells treated with 20 μM CCCP for indicated timepoints. Percentage of apoptosis (stained with Annexin V+/PI-) or necrotosis/pyroptosis (stained with Annexin V-/PI+ or Annexin V+/PI+) was indicated. (**G** and **H**) Representative images and average fluorescence intensity (AU) of SYTOX Green staining in HeLa YFP-Parkin WT/PTEN-L KO cells following treatment with 20 μM CCCP for 12 h. Scale bar, 20 μm. (**I**) Representative microscopy images snapped by confocal microscopy of HeLa YFP-Parkin PTEN-L KO cells rescued with mCherry empty control (mCherry-EV) or mCherry-PTEN-L (mCherry-PL) constructs following treatment with DMSO for 12 h, as control of **Figure 1K**. Scale bar, 20 μm. (**J** and **K**) Representative microscopy images snapped by confocal microscopy and

LDH release assay of HeLa YFP-Parkin PTEN-L KO cells rescued with mCherry empty control (mCherry-EV) or mCherry-PTEN-L (mCherry-PL) constructs following treatment with 20 nM/200 nM O/A for 12h. Scale bar, 20 μm. Red arrows in (**A**) and (**B**) indicate pyroptotic cells. Immunoblots in (**E**) are representative of three independent biological replicates. Data in (**F**), (**H**) and (**K**) are presented as mean ± SD from three independent biological replicates. Statistical significance was assessed by two-way ANOVA.

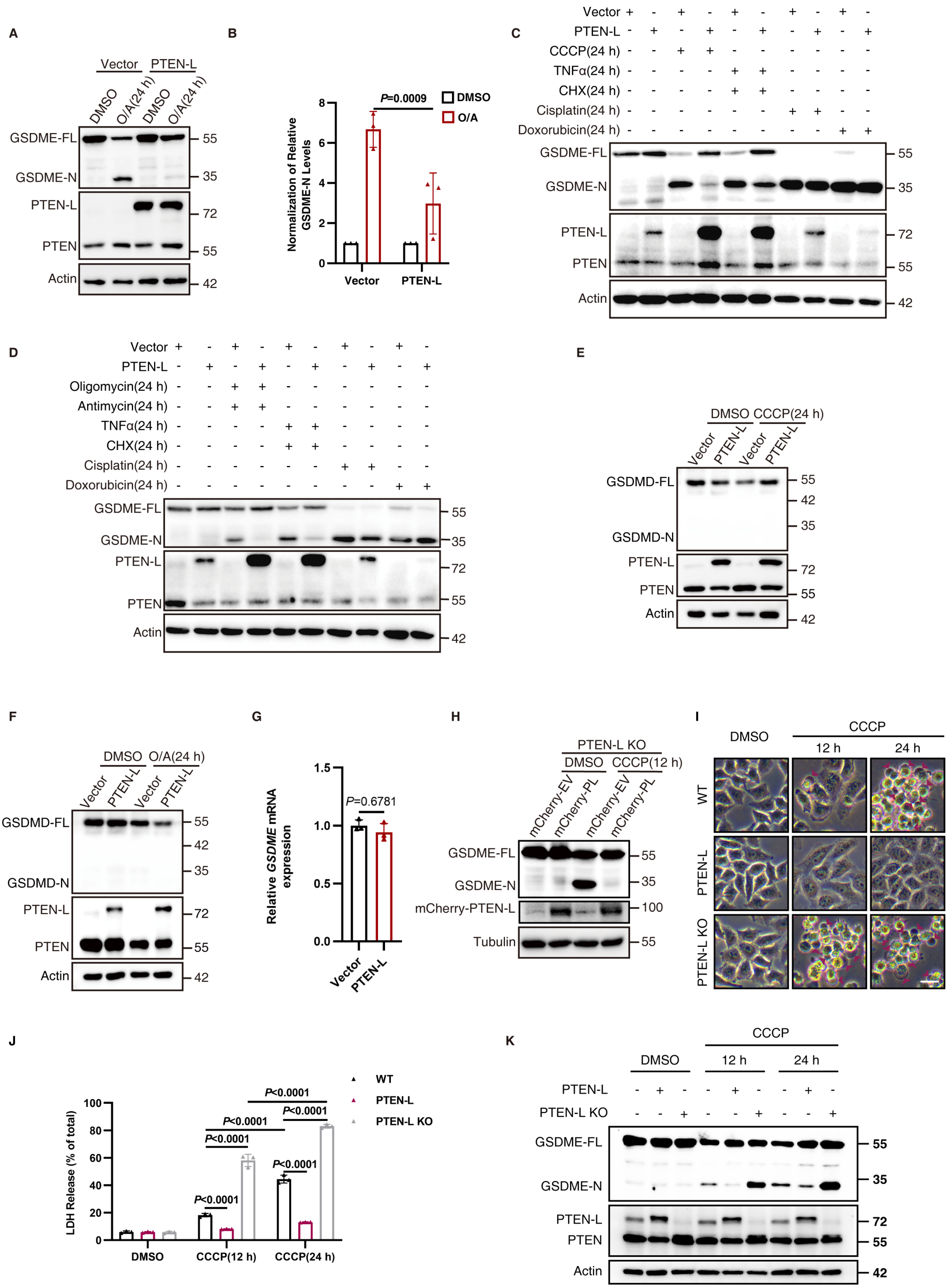
A
Vector
PTEN-L
DMSO
O/A(24 h)
DMSO
O/A(24 h)
GSDME-FL
GSDME-N
PTEN-L
PTEN
Actin
55
35
72
55
42
B
P=0.0009
DMSO
O/A
Normalization of Relative GSDME-N Levels
0
2
4
6
8
Vector
PTEN-L
C
Vector + - + - + - + - + -
PTEN-L - + - + - + - + - +
CCCP(24 h) - - + + - - - - - -
TNFα(24 h) - - - - + + - - - -
CHX(24 h) - - - - + + - - - -
Cisplatin(24 h) - - - - - - + + - -
Doxorubicin(24 h) - - - - - - - - + +
GSDME-FL
GSDME-N
PTEN-L
PTEN
Actin
55
35
72
55
42
D
Vector + - + - + - + - + -
PTEN-L - + - + - + - + - +
Oligomycin(24 h) - - + + - - - - - -
Antimycin(24 h) - - + + - - - - - -
TNFα(24 h) - - - - + + - - - -
CHX(24 h) - - - - + + - - - -
Cisplatin(24 h) - - - - - - + + - -
Doxorubicin(24 h) - - - - - - - - + +
GSDME-FL
GSDME-N
PTEN-L
PTEN
Actin
55
35
72
55
42
E
DMSO
CCCP(24 h)
Vector
PTEN-L
Vector
PTEN-L
GSDMD-FL
GSDMD-N
PTEN-L
PTEN
Actin
55
42
35
72
55
42
F
DMSO
O/A(24 h)
Vector
PTEN-L
Vector
PTEN-L
GSDMD-FL
GSDMD-N
PTEN-L
PTEN
Actin
55
42
35
72
55
42
G
P=0.6781
Relative GSDME mRNA expression
0.0
0.5
1.0
1.5
Vector
PTEN-L
H
PTEN-L KO
DMSO
CCCP(12 h)
mCherry-EV
mCherry-PL
mCherry-EV
mCherry-PL
GSDME-FL
GSDME-N
mCherry-PTEN-L
Tubulin
55
35
100
55
I
DMSO
CCCP
12 h
24 h
WT
PTEN-L
PTEN-L KO
J
WT
PTEN-L
PTEN-L KO
LDH Release (% of total)
0
20
40
60
80
100
P<0.0001
P<0.0001
P<0.0001
P<0.0001
P<0.0001
P<0.0001
DMSO
CCCP(12 h)
CCCP(24 h)
K
CCCP
DMSO
12 h
24 h
PTEN-L - + - - + - - + -
PTEN-L KO - - + - - + - - +
GSDME-FL
GSDME-N
PTEN-L
PTEN
Actin
55
35
72
55
42

**Appendix Figure S4. PTEN-L inhibits GSDME cleavage and activation.** (**A** and **B**) Immunoblotting and quantitative analysis of GSDME cleavage and activation in lysates of HeLa YFP-Parkin Vector/PTEN-L cells following treatment with 20 nM/200 nM O/A for 24 h. (**C**) Immunoblotting analysis of GSDME cleavage and activation in lysates of HeLa YFP-Parkin Vector/PTEN-L cells following treatment with 20 μM CCCP, 20 ng/mL TNFα plus 10 ug/mL CHX, 20 μg/mL Cisplatin or 1 μM Doxorubicin for 24 h. (**D**) Immunoblotting analysis of GSDME cleavage and activation in lysates of HeLa YFP-Parkin Vector/PTEN-L cells following treatment with 20 nM/200 nM O/A, 20 ng/mL TNFα plus 10 ug/mL CHX, 20 μg/mL Cisplatin or 1 μM Doxorubicin for 24 h. (**E** and **F**) Immunoblotting analysis of GSDMD cleavage and activation in lysates of HeLa YFP-Parkin Vector/PTEN-L cells following treatment with 20 μM CCCP or 20 nM/200 nM O/A for 24 h. (**G**) Quantitative polymerase chain reaction to evaluate the relative mRNA expression of *GSDME* genes in HeLa YFP-Parkin Vector/PTEN-L cells. (**H**) Immunoblotting analysis of GSDME cleavage and activation in lysates of HeLa YFP-Parkin PTEN-L KO cells rescued with mCherry empty control (mCherry-EV) or mCherry-PTEN-L (mCherry-PL) constructs following treatment with 20 μM CCCP for 12 h. (**I** and **J**) Representative microscopy images and LDH release assay of HeLa YFP-Parkin WT/HeLa YFP-Parkin PTEN-L/PTEN-L KO cells following treatment with 20 μM CCCP for 12h or 24 h. Scale bar, 25 μm. (**K**) Immunoblotting analysis of GSDME cleavage and activation in lysates of HeLa YFP-Parkin PTEN-L/PTEN-L KO cells following treatment with 20 μM CCCP for 12h or 24 h. Red arrows in (**I**) indicate

pyroptotic cells. Immunoblots in (**C**) and (**D**) are representative of three independent biological replicates. Immunoblots in (**A**), (**E**), (**F**), (**H**) and (**K**) are representative of two independent biological replicates. Data in (**B**), (**G**), and (**J**) are presented as mean ± SD from three independent biological replicates. Statistical significance was assessed by two-way ANOVA (**B**), (**J**) and two-tailed unpaired Student's-t test (**G**).

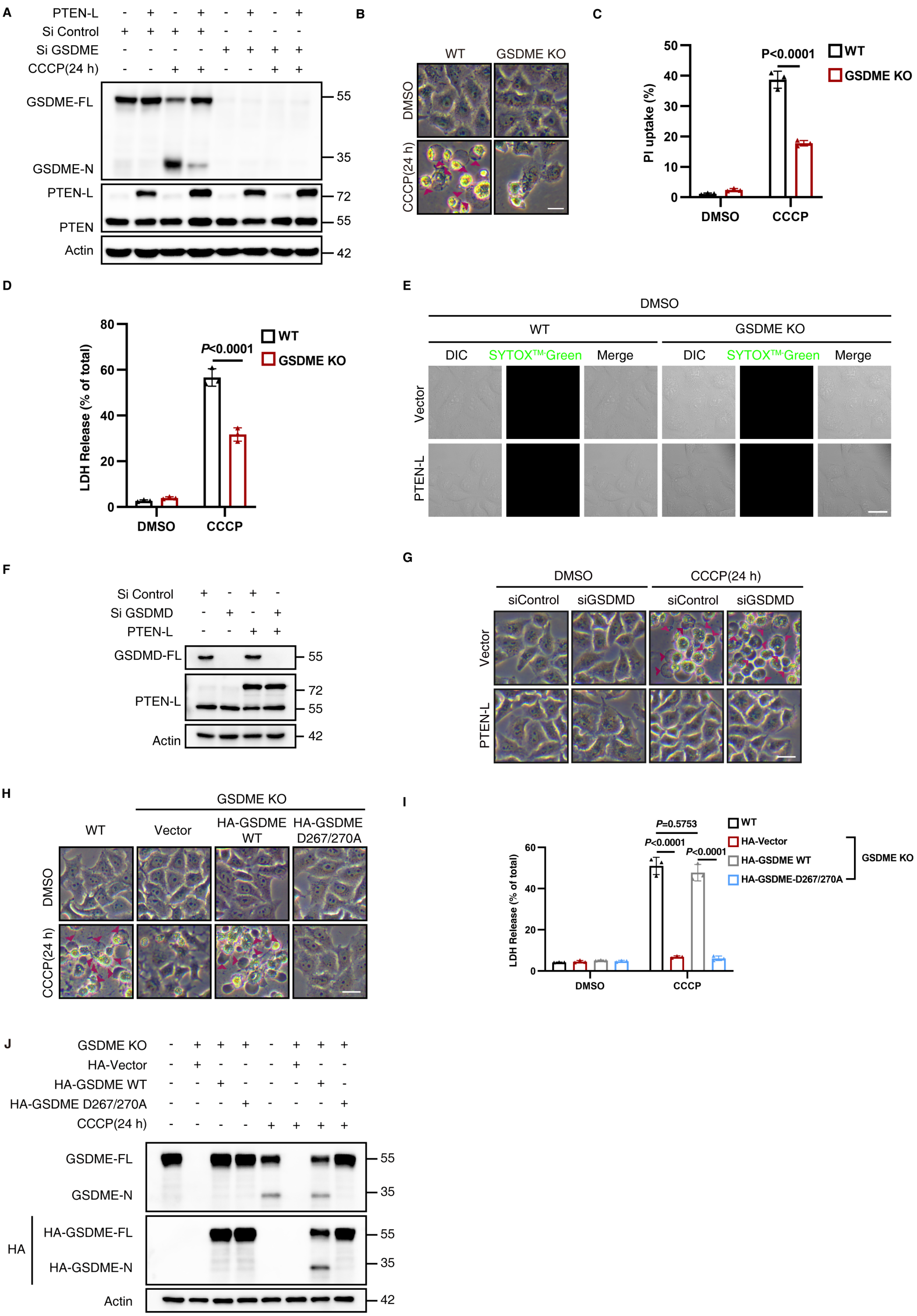
A
PTEN-L
Si Control
Si GSDME
CCCP(24 h)
GSDME-FL
GSDME-N
PTEN-L
PTEN
Actin
55
35
72
55
42
B
WT
GSDME KO
DMSO
CCCP(24 h)
C
PI uptake (%)
P<0.0001
WT
GSDME KO
DMSO
CCCP
D
LDH Release (% of total)
P<0.0001
WT
GSDME KO
DMSO
CCCP
E
DMSO
WT
GSDME KO
DIC
SYTOXTM-Green
Merge
Vector
PTEN-L
F
Si Control
Si GSDMD
PTEN-L
GSDMD-FL
PTEN-L
Actin
55
72
55
42
G
DMSO
CCCP(24 h)
siControl
siGSDMD
Vector
PTEN-L
H
GSDME KO
WT
Vector
HA-GSDME WT
HA-GSDME D267/270A
DMSO
CCCP(24 h)
I
LDH Release (% of total)
P=0.5753
P<0.0001
P<0.0001
WT
HA-Vector
HA-GSDME WT
HA-GSDME-D267/270A
GSDME KO
DMSO
CCCP
J
GSDME KO
HA-Vector
HA-GSDME WT
HA-GSDME D267/270A
CCCP(24 h)
GSDME-FL
GSDME-N
HA
HA-GSDME-FL
HA-GSDME-N
Actin
55
35
55
35
42

**Appendix Figure S5. Knockdown or knockout of GSDME, rather than GSDMD, suppresses CCCP-induced pyroptosis.** (**A**) Immunoblotting analysis of GSDME cleavage and activation in lysates of HeLa YFP-Parkin Vector/PTEN-L cells transfected with nontargeting control siRNA (Si Control) or siRNA targeted against GSDME (Si GSDME) treated with 20 μM CCCP for 24 h. (**B** to **D**) Representative microscopy images, PI uptake and LDH release assay of HeLa YFP-Parkin WT/GSDME KO cells treated with 20 μM CCCP for 24 h. Scale bar, 25 μm. (**E**) Representative images of SYTOX Green staining in HeLa YFP-Parkin Vector/PTEN-L cells with GSDME knockout (GSDME KO) created via CRISPR/Cas9 system. Scale bars, 20 μm. (**F**) Immunoblotting analysis of HeLa YFP-Parkin Vector/PTEN-L cells transfected with nontargeting control siRNA (Si Control) or siRNA targeted against GSDMD (Si GSDMD). (**G**) Representative microscopy images of HeLa YFP-Parkin Vector/PTEN-L cells transfected with nontargeting control siRNA (Si Control) or siRNA targeted against GSDMD (Si GSDMD) with 20 μM CCCP for 24 h. Scale bar, 25 μm. (**H** and **I**) Representative microscopy images and LDH release assay of HeLa YFP-Parkin WT and HeLa YFP-Parkin GSDME KO rescued with HA empty control (pCDH-HA-Vector), pCDH-HA-GSDME-WT or pCDH-HA-GSDME-D267/270A constructs following treatment with 20 μM CCCP for 24 h. Scale bar, 25 μm. (**J**) Immunoblotting analysis of GSDME cleavage and activation in lysates of HeLa YFP-Parkin WT and HeLa YFP-Parkin GSDME KO rescued with HA empty control (pCDH-HA-Vector), pCDH-HA-GSDME-WT or ppCDH-HA-GSDME-D267/270A constructs following

treatment with 20 μM CCCP for 24 h. Red arrows in (**B**), (**G**), and (**H**) indicate pyroptotic cells. Immunoblots in (**F**) are representative of two independent biological replicates. Immunoblots in (**A**) and (**J**) are representative of three independent biological replicates. Data in (**C**), (**D**) and (**I**) are presented as mean ± SD from three independent biological replicates. Statistical significance was assessed by two-way ANOVA.

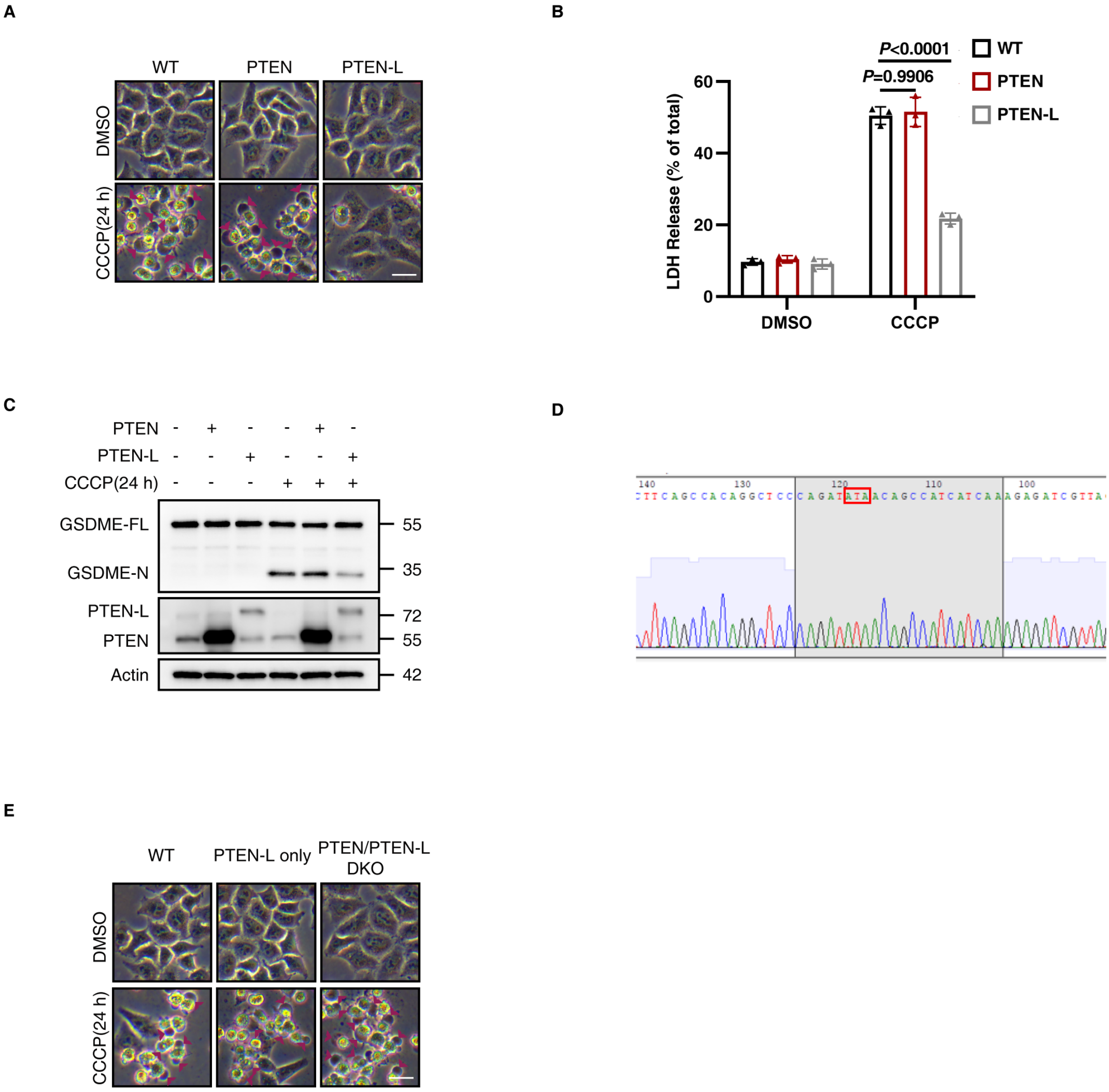
A
WT
PTEN
PTEN-L
DMSO
CCCP(24 h)
B
P<0.0001
P=0.9906
WT
PTEN
PTEN-L
LDH Release (% of total)
0
20
40
60
DMSO
CCCP
C
PTEN - + - - + -
PTEN-L - - + - - +
CCCP(24 h) - - - + + +
GSDME-FL
GSDME-N
PTEN-L
PTEN
Actin
55
35
72
55
42
D
140
130
120
110
100
E
WT
PTEN-L only
PTEN/PTEN-L
DKO
DMSO
CCCP(24 h)

**Appendix Figure S6. PTEN-L, but not canonical PTEN, is responsible for suppressing cell death upon mitochondrial depolarization.** (**A** and **B**) Representative microscopy images and LDH release assay of HeLa YFP-Parkin WT, HeLa YFP-Parkin PTEN or PTEN-L cells following treatment with 20 μM CCCP for 24 h. Scale bar, 25 μm. (**C**) Immunoblotting analysis of GSDME cleavage and activation in lysates of HeLa YFP-Parkin WT, HeLa YFP-Parkin PTEN or PTEN-L cells following treatment with 20 μM CCCP for 24 h. (**D**) Validation of PTEN-L only construct by Sanger sequencing. (**E**) Representative microscopy images of HeLa YFP-Parkin WT, HeLa YFP-Parkin PTEN-L only and HeLa YFP-Parkin PTEN/PTEN-L DKO cells with 20 μM CCCP for 24 h. Scale bar, 25 μm. Red arrows in (**A**), and (**E**) indicate pyroptotic cells. Immunoblots in (**C**) are representative of three independent biological replicates. Data in (**B**) are presented as mean ± SD from three independent biological replicates. Statistical significance was assessed by two-way ANOVA.

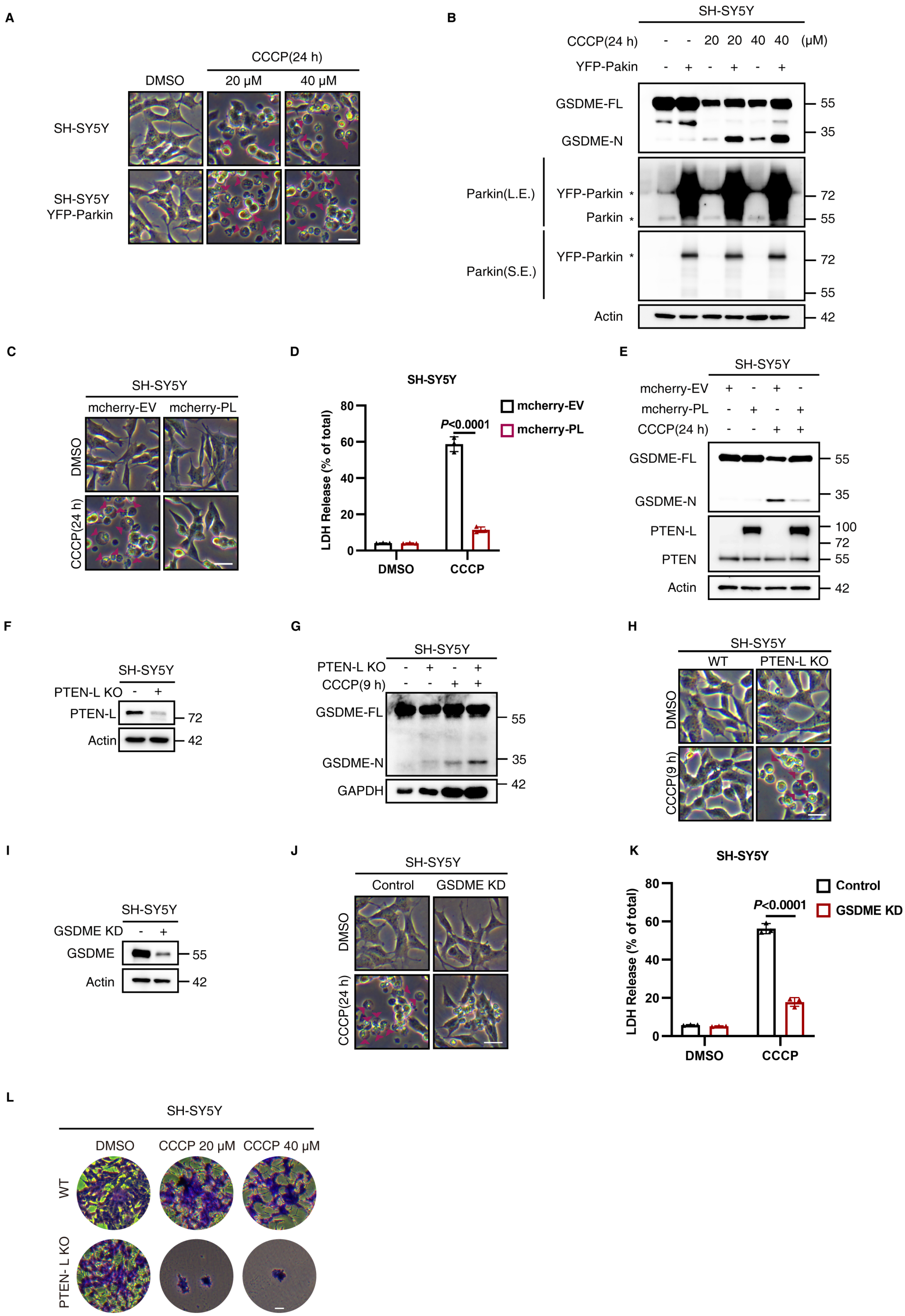

A
CCCP(24 h)
DMSO
20 μM
40 μM
SH-SY5Y
SH-SY5Y YFP-Parkin
B
SH-SY5Y
CCCP(24 h) - - 20 20 40 40 (μM)
YFP-Pakin - + - + - +
GSDME-FL
GSDME-N
55
35
Parkin(L.E.)
YFP-Parkin *
Parkin *
72
55
Parkin(S.E.)
YFP-Parkin *
72
55
Actin
42
C
SH-SY5Y
mcherry-EV
mcherry-PL
DMSO
CCCP(24 h)
D
SH-SY5Y
LDH Release (% of total)
80
60
40
20
0
P<0.0001
mcherry-EV
mcherry-PL
DMSO
CCCP
E
SH-SY5Y
mcherry-EV + - + -
mcherry-PL - + - +
CCCP(24 h) - - + +
GSDME-FL
GSDME-N
PTEN-L
PTEN
Actin
55
35
100
72
55
42
F
SH-SY5Y
PTEN-L KO - +
PTEN-L
Actin
72
42
G
SH-SY5Y
PTEN-L KO - + - +
CCCP(9 h) - - + +
GSDME-FL
GSDME-N
GAPDH
55
35
42
H
SH-SY5Y
WT
PTEN-L KO
DMSO
CCCP(9 h)
I
SH-SY5Y
GSDME KD - +
GSDME
Actin
55
42
J
SH-SY5Y
Control
GSDME KD
DMSO
CCCP(24 h)
K
SH-SY5Y
LDH Release (% of total)
80
60
40
20
0
P<0.0001
Control
GSDME KD
DMSO
CCCP
L
SH-SY5Y
DMSO
CCCP 20 μM
CCCP 40 μM
WT
PTEN- L KO

**Appendix Figure S7. PTEN-L suppresses pyroptosis by inhibiting GSDME cleavage in SH-SY5Y cells.** (**A**) Representative microscopy images of SH-SY5Y WT/ YFP-Parkin cells with 20 μM or 40 μM CCCP for 24 h. Scale bar, 25 μm. (**B**) Immunoblotting analysis of GSDME cleavage and activation in lysates of SH-SY5Y WT/YFP-Parkin cells following treatment with 20 μM or 40 μM CCCP for 24 h. S.E, short exposure. L.E, long exposure. (**C** to **E**) Representative microscopy images, LDH release assay, and immunoblotting analysis of SH-SY5Y mcherry-EV or mcherry-PL (PL) cells following treatment with 40 μM CCCP for 24 h. Scale bar, 25 μm. (**F**) SH-SY5Y cells with PTEN-L knockout (PTEN-L KO) created via CRISPR/Cas9 were subjected to immunoblotting as indicated. (**G** and **H**) Representative microscopy images and immunoblotting analysis of SH-SY5Y WT/PTEN-L KO cells following treatment with 40 μM CCCP for 9 h. Scale bar, 25 μm. (**I**) SH-SY5Y cells with GSDME knockdown (GSDME KD) were subjected to immunoblotting as indicated. (**J** and **K**) Representative microscopy images and LDH release assay of SH-SY5Y Control/GSDME KD cells with 40 μM CCCP for 24 h. Scale bar, 25 μm. (**L**) Colony formation assay of SH-SY5Y WT/PTEN-L KO cells following treatment with 20 μM or 40 μM CCCP for 12 h and 5 more days' normal cultivation. Scale bar, 25 μm. Red arrows in (**A**), (**C**), (**H**) and (**J**) indicate pyroptotic cells. Microscopy images in (**A**) are representative of three independent biological replicates. Immunoblots in (**F**) and (**I**) are representative of two independent biological replicates. Immunoblots in (**B**), (**E**) and (**G**) are representative of three independent biological replicates. Data in (**D**), and (**K**) are presented as mean ± SD from three independent biological replicates.

Statistical significance was assessed by two-way ANOVA.

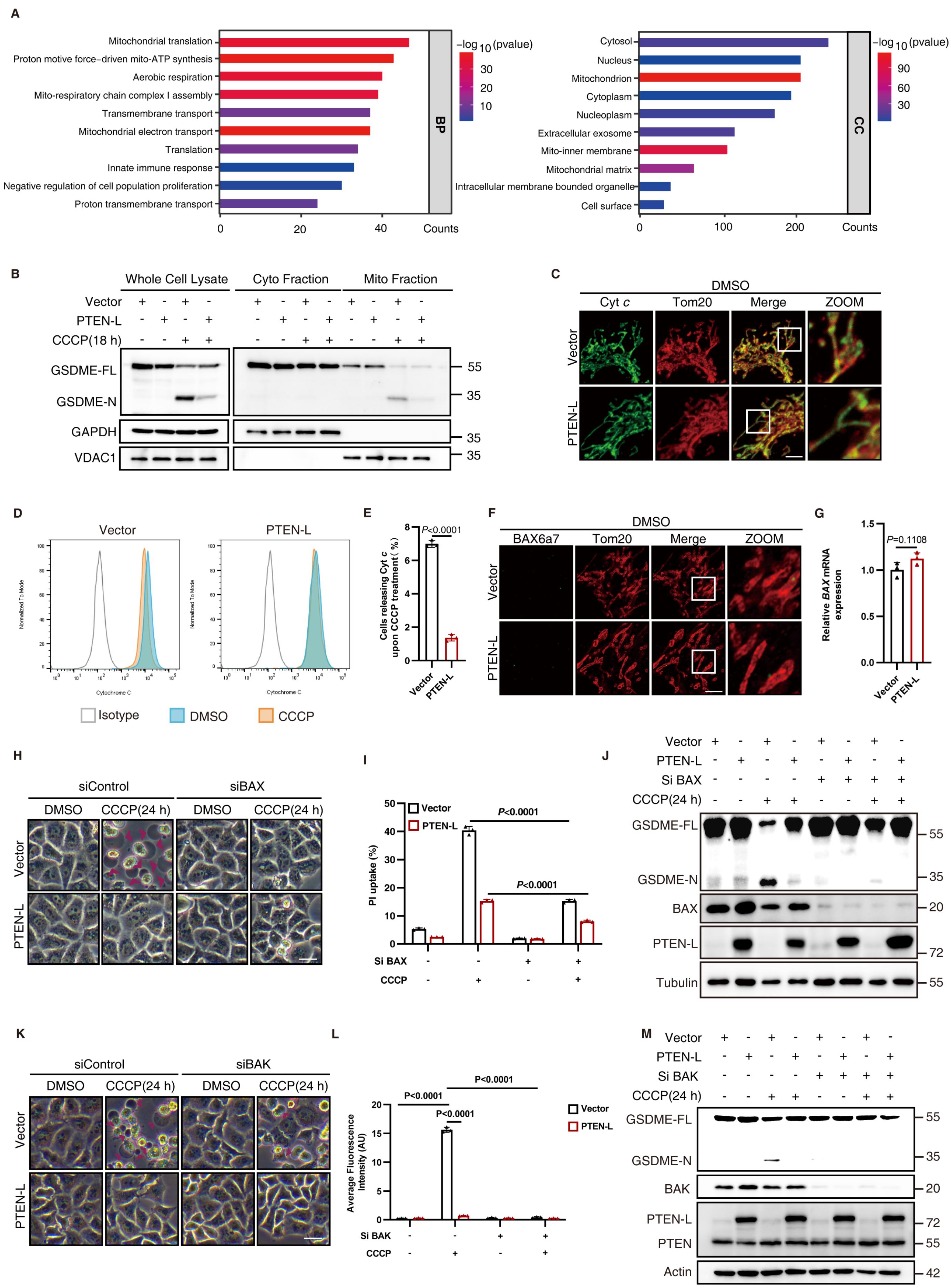
A
Mitochondrial translation
Proton motive force-driven mito-ATP synthesis
Aerobic respiration
Mito-respiratory chain complex I assembly
Transmembrane transport
Mitochondrial electron transport
Translation
Innate immune response
Negative regulation of cell population proliferation
Proton transmembrane transport
BP
Counts
−log10 (pvalue)
Cytosol
Nucleus
Mitochondrion
Cytoplasm
Nucleoplasm
Extracellular exosome
Mito-inner membrane
Mitochondrial matrix
Intracellular membrane bounded organelle
Cell surface
CC
B
Whole Cell Lysate
Cyto Fraction
Mito Fraction
Vector
PTEN-L
CCCP(18 h)
GSDME-FL
GSDME-N
GAPDH
VDAC1
C
DMSO
Cyt c
Tom20
Merge
ZOOM
D
Cytochrome C
Isotype
DMSO
CCCP
E
P<0.0001
Cells releasing Cyt c upon CCCP treatment ( %)
F
BAX6a7
G
P=0.1108
Relative BAX mRNA expression
H
siControl
siBAX
CCCP(24 h)
I
PI uptake (%)
Si BAX
CCCP
J
Si BAX
CCCP(24 h)
BAX
Tubulin
K
siBAK
L
Average Fluorescence Intensity (AU)
Si BAK
M
BAK
PTEN
Actin

**Appendix Figure S8. PTEN-L affects mitochondrial pathway.** (**A**) Gene ontology (GO) analysis of proteomics data of Hela YFP-Parkin Vector/PTEN-L cell lysates following treatment with 20 μM CCCP for 18 h. The data were obtained from the GO database, with the upper one pertaining to Biological Process (BP) and the lower one referring to Cellular Component (CC). (**B**) Immunoblotting detection of GSDME-N mitochondria translocation of HeLa YFP-Parkin Vector/PTEN-L cells following treatment with 20 μM CCCP for 18 h, followed by cell mitochondria isolation. (**C**) Immunostaining assay to detect cytochrome c (Cyt c) release of HeLa YFP-Parkin Vector/PTEN-L cells following treatment with DMSO for 6 h, as control of **Figure 3B**. Scale bar, 5 μm. (**D** and **E**) Detection and quantitative analysis of Cyt c release of HeLa YFP-Parkin Vector/PTEN-L cells following treatment with 20 μM CCCP for 6h by flow cytometry. (**F**) Immunostaining assay to assess the presence of active BAX (BAX6a7) on mitochondria of HeLa YFP-Parkin Vector/PTEN-L cells, as control of **Figure 3E**. Scale bar, 5 μm. (**G**) Quantitative polymerase chain reaction to evaluate the relative mRNA expression of *BAX* genes in HeLa YFP-Parkin Vector/PTEN-L cells. (**H** to **J**) Representative microscopy images, PI uptake, and GSDME cleavage and activation assay of HeLa YFP-Parkin Vector/PTEN-L cells transfected with nontargeting control siRNA (Si Control) or siRNA targeted against BAX (Si BAX) with 20 μM CCCP for 24 h. Scale bar, 25 μm. (**K** to **M**) Representative microscopy images, average fluorescence intensity (AU) of SYTOX Green staining and GSDME cleavage activation assay of HeLa YFP-Parkin Vector/PTEN-L cells transfected with nontargeting control siRNA (Si Control) or siRNA targeted against BAK (Si BAK)

with 20 μM CCCP for 24 h. Scale bar, 25 μm. Red arrows in (**H**), and (**K**) indicate pyroptotic cells. Immunoblots in (**B**), (**J**), and (**M**) are representative of three independent biological replicates. Data in (**E**), (**G**), (**I**), and (**L**) are presented as mean ± SD from three independent biological replicates. Statistical significance was assessed by two-tailed unpaired Student's-t test (**E**), (**G**), and two-way ANOVA (**I**), (**L**).

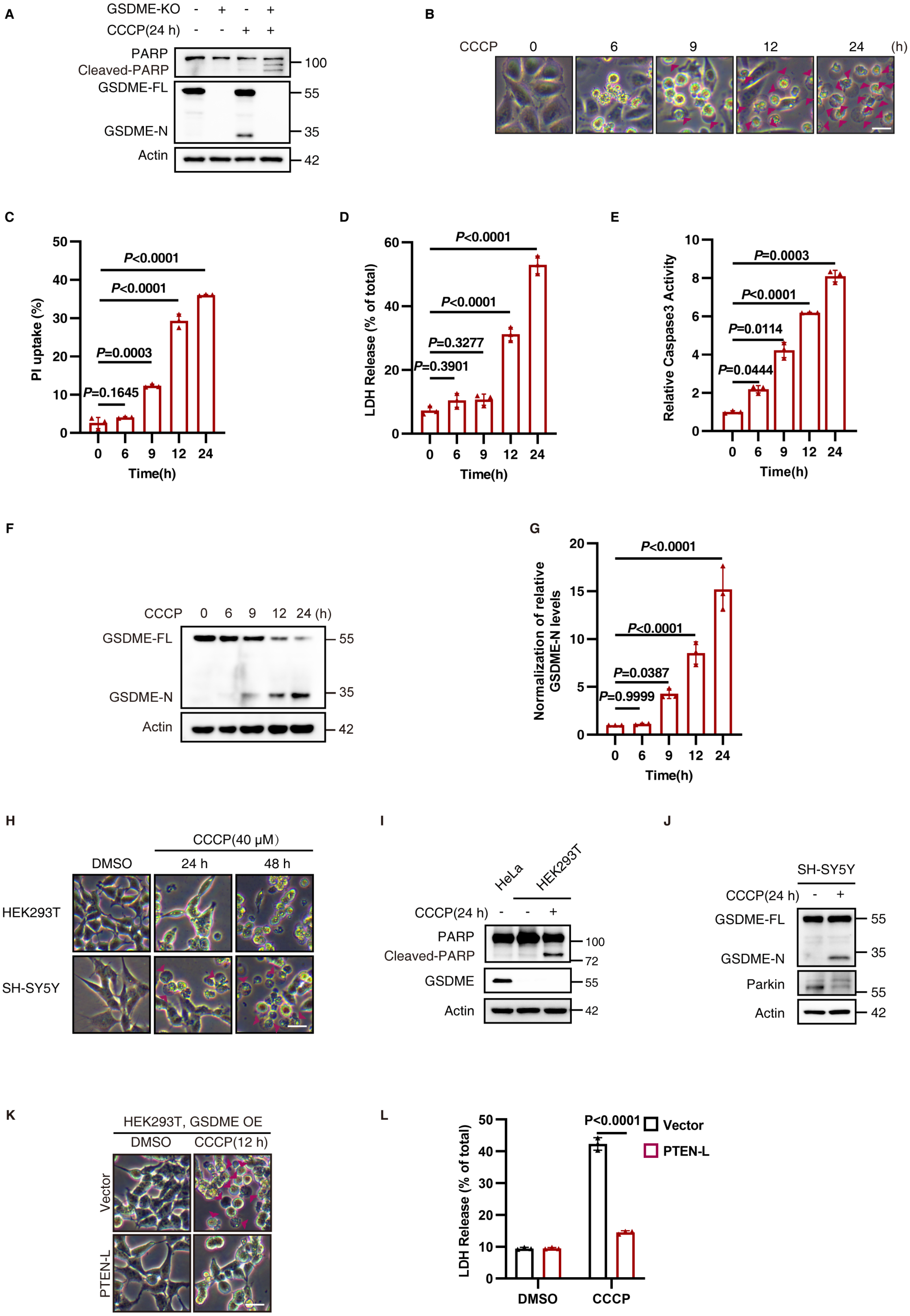
A
GSDME-KO - + - +
CCCP(24 h) - - + +
PARP
Cleaved-PARP
GSDME-FL
GSDME-N
Actin
100
55
35
42
B
CCCP 0 6 9 12 24 (h)
C
PI uptake (%)
P<0.0001
P<0.0001
P=0.0003
P=0.1645
Time(h)
0 6 9 12 24
D
LDH Release (% of total)
P<0.0001
P<0.0001
P=0.3277
P=0.3901
Time(h)
0 6 9 12 24
E
Relative Caspase3 Activity
P=0.0003
P<0.0001
P=0.0114
P=0.0444
Time(h)
0 6 9 12 24
F
CCCP 0 6 9 12 24 (h)
GSDME-FL
GSDME-N
Actin
55
35
42
G
Normalization of relative GSDME-N levels
P<0.0001
P<0.0001
P=0.0387
P=0.9999
Time(h)
0 6 9 12 24
H
CCCP(40 μM)
DMSO 24 h 48 h
HEK293T
SH-SY5Y
I
HeLa HEK293T
CCCP(24 h) - - +
PARP
Cleaved-PARP
GSDME
Actin
100
72
55
42
J
SH-SY5Y
CCCP(24 h) - +
GSDME-FL
GSDME-N
Parkin
Actin
55
35
55
42
K
HEK293T, GSDME OE
DMSO CCCP(12 h)
Vector
PTEN-L
L
LDH Release (% of total)
P<0.0001
Vector
PTEN-L
DMSO CCCP

**Appendix Figure S9. Time-course analysis of cell death upon CCCP treatment.** (**A**) Immunoblotting analysis of PARP cleavage in lysates of HeLa YFP-Parkin cells with GSDME knockout (GSDME KO) created via CRISPR/Cas9 system following treatment with 20 μM CCCP for 24 h. (**B** to **E**) Representative microscopy images, PI uptake, LDH release and relative Caspase3 activity assay of HeLa HA-Parkin cells following treatment with 20 μM CCCP for 0, 6, 9, 12 or 24 h. Scale bar, 25 μm. (**F** and **G**) Immunoblotting and quantitative analysis of GSDME cleavage and activation in lysates of HeLa HA-Parkin cells following treatment with 20 μM CCCP for 0, 6, 9, 12 or 24 h. (**H**) Representative microscopy images of HEK293T and SH-SY5Y cells following treatment with 40 μM CCCP for 24 h or 48h. Scale bar, 25 μm. (**I**) Immunoblotting analysis of PARP cleavage in lysates of HeLa and HEK293T cells treatmented with 20 μM CCCP for 24 h. (**J**) Immunoblotting analysis of GSDME cleavage in lysates of SH-SY5Y cells following treatment with 20 μM CCCP for 24 h. (**K** and **L**) Representative microscopy images and LDH release assay of HEK293T cells transiently transfected with pEGFP-C1 vector/PTEN-L, mCherry-Parkin, 3×Flag-GSDME following treatment with 20 μM CCCP for 12 h. Scale bar, 25 μm. Red arrows in (**B**), (**H**) and (**K**) indicate pyroptotic cells. Immunoblots in (**A**), (**I**), and (**J**) are representative of three independent biological replicates. Immunoblots in (**F**) are representative of four independent biological replicates. Data in (**C**), (**D**), (**E**), (**G**) and (**L**) are presented as mean ± SD from three independent biological replicates. Statistical significance was assessed by two-tailed unpaired Student's-t test (**C**), (**D**), (**E**), (**G**) or two-way ANOVA (**L**).

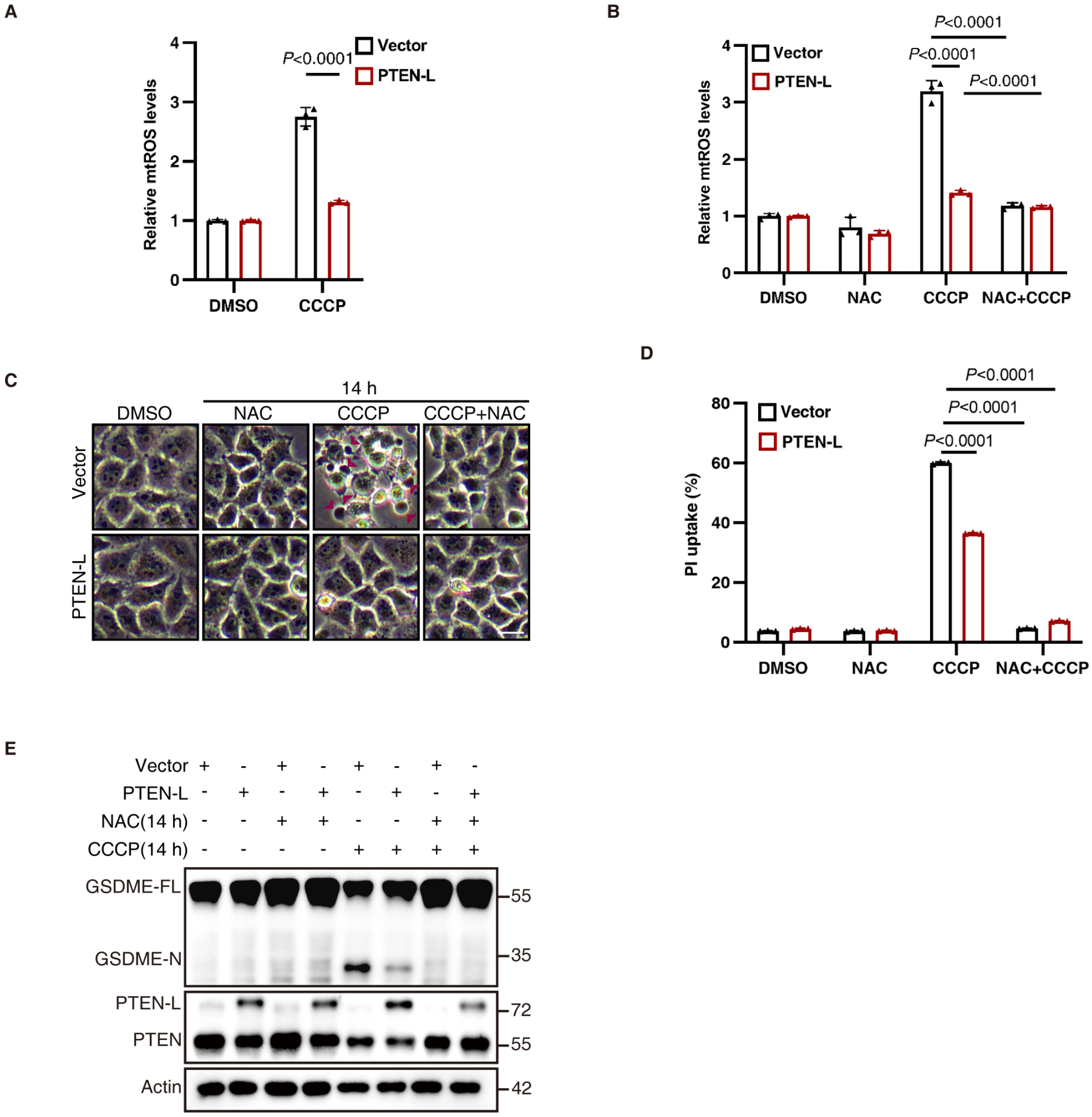

A
Relative mtROS levels
P<0.0001
Vector
PTEN-L
DMSO
CCCP
B
Relative mtROS levels
Vector
PTEN-L
P<0.0001
P<0.0001
P<0.0001
DMSO
NAC
CCCP
NAC+CCCP
C
14 h
DMSO
NAC
CCCP
CCCP+NAC
Vector
PTEN-L
D
PI uptake (%)
Vector
PTEN-L
P<0.0001
P<0.0001
P<0.0001
DMSO
NAC
CCCP
NAC+CCCP
E
Vector
PTEN-L
NAC(14 h)
CCCP(14 h)
GSDME-FL
GSDME-N
PTEN-L
PTEN
Actin
55
35
72
55
42

**Appendix Figure S10. PTEN-L suppresses pyroptosis through inhibition of mitochondrial ROS production.** (**A**) Mitochondrial reactive oxygen species (mtROS) levels measured with MitoSOX Red using a flow cytometer of HeLa YFP-Parkin Vector/PTEN-L cells following treatment with 20 μM CCCP for 14 h. (**B** to **D**) mtROS levels, representative microscopy images, and PI uptake assay of HeLa YFP-Parkin Vector/PTEN-L cells pretreated with 5 mM NAC for 2 h following cotreatment with 20 μM CCCP for 14 h. Scale bar, 25 μm. (**E**) Immunoblotting analysis for GSDME cleavage and activation of HeLa YFP-Parkin Vector/PTEN-L cell lysates pretreated with 5 mM NAC for 2 h following cotreatment with 20 μM CCCP for 14 h. Red arrows in (**C**) indicate pyroptotic cells. Immunoblots in (**E**) are representative of three independent biological replicates. Data in (**A**), (**B**), and (**D**) are presented as mean ± SD from three independent biological replicates. Statistical significance was assessed by two-way ANOVA.

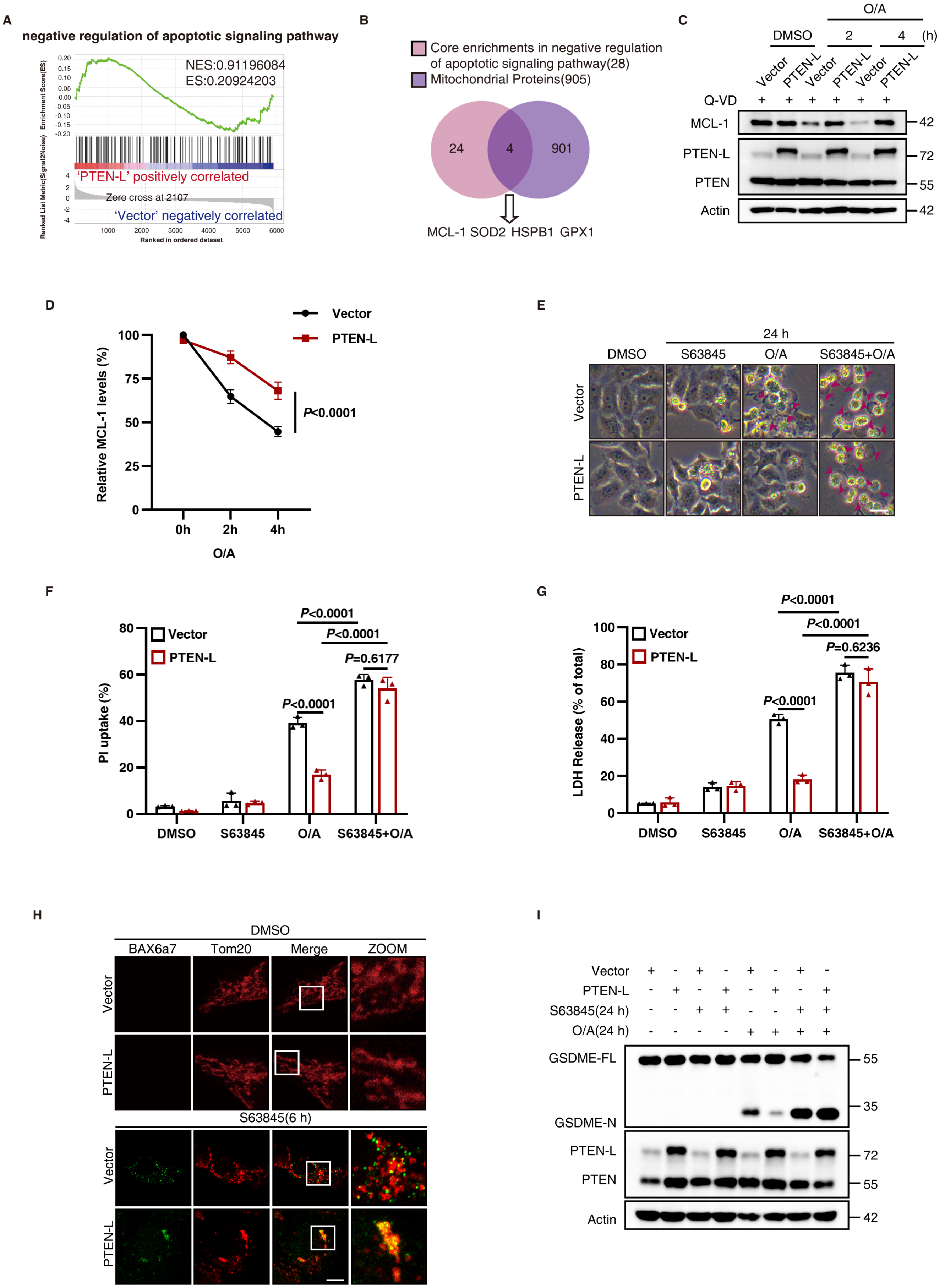
A
negative regulation of apoptotic signaling pathway
NES:0.91196084
ES:0.20924203
'PTEN-L' positively correlated
Zero cross at 2107
'Vector' negatively correlated
Ranked in ordered dataset
B
Core enrichments in negative regulation of apoptotic signaling pathway(28)
Mitochondrial Proteins(905)
24
4
901
MCL-1 SOD2 HSPB1 GPX1
C
O/A
DMSO
2
4
(h)
Vector PTEN-L Vector PTEN-L Vector PTEN-L
Q-VD
MCL-1
PTEN-L
PTEN
Actin
D
Vector
PTEN-L
Relative MCL-1 levels (%)
P<0.0001
0h 2h 4h
O/A
E
24 h
DMSO S63845 O/A S63845+O/A
Vector
PTEN-L
F
PI uptake (%)
Vector
PTEN-L
P<0.0001
P<0.0001
P=0.6177
P<0.0001
DMSO S63845 O/A S63845+O/A
G
LDH Release (% of total)
Vector
PTEN-L
P<0.0001
P<0.0001
P=0.6236
P<0.0001
DMSO S63845 O/A S63845+O/A
H
DMSO
BAX6a7 Tom20 Merge ZOOM
Vector
PTEN-L
S63845(6 h)
Vector
PTEN-L
I
Vector
PTEN-L
S63845(24 h)
O/A(24 h)
GSDME-FL
GSDME-N
PTEN-L
PTEN
Actin

**Appendix Figure S11. MCL-1 inhibition induces pyroptosis following mitochondrial depolarization.** (**A**) Gene Set Enrichment Analysis (GSEA) of proteomics data of Hela YFP-Parkin Vector/PTEN-L cell lysates following treatment with 20 μM CCCP for 2 h. The data were acquired from the Gene Ontology (GO) database. NES represents Normalized Enrichment Score and ES is an abbreviation of Enrichment Score. (**B**) Venn diagram for core enrichments in negative regulation of apoptotic signaling pathway and mitochondrial proteins. (**C** and **D**) Immunoblotting and quantitative analysis of MCL-1 level in lysates of HeLa YFP-Parkin Vector/PTEN-L cells following treatment with 40 μM Q-VD-OPh (Q-VD) and 20 nM/200 nM O/A for 2 h or 4 h. (**E** to **G**) Representative microscopy images, PI uptake and LDH release assay in HeLa YFP-Parkin Vector/PTEN-L cells following treatment with 3 μM S63845, 20 nM/200 nM O/A, or 3 μM S63845 plus 20 nM/200 nM O/A for 24 h. Scale bar, 25 μm. (**H**) Immunostaining assay to assess the presence of active BAX (BAX6a7) on mitochondria of HeLa YFP-Parkin Vector/PTEN-L cells following treatment with 3 μM S63845 or not for 6 h Scale bar, 5 μm. (**I**) Immunoblotting of GSDME cleavage and activation in lysates of HeLa YFP-Parkin Vector/PTEN-L cells following treatment with 3 μM S63845, 20 nM/200 nM O/A, or 3 μM S63845 plus 20 nM/200 nM O/A for 24 h. Red arrows in (**E**) indicate pyroptotic cells. Immunoblots in (**C**) and (**I**) are representative of three independent biological replicates. Data in (**D**), (**F**), and (**G**) are presented as mean ± SD from three independent biological replicates. Statistical significance was assessed by two-way ANOVA.

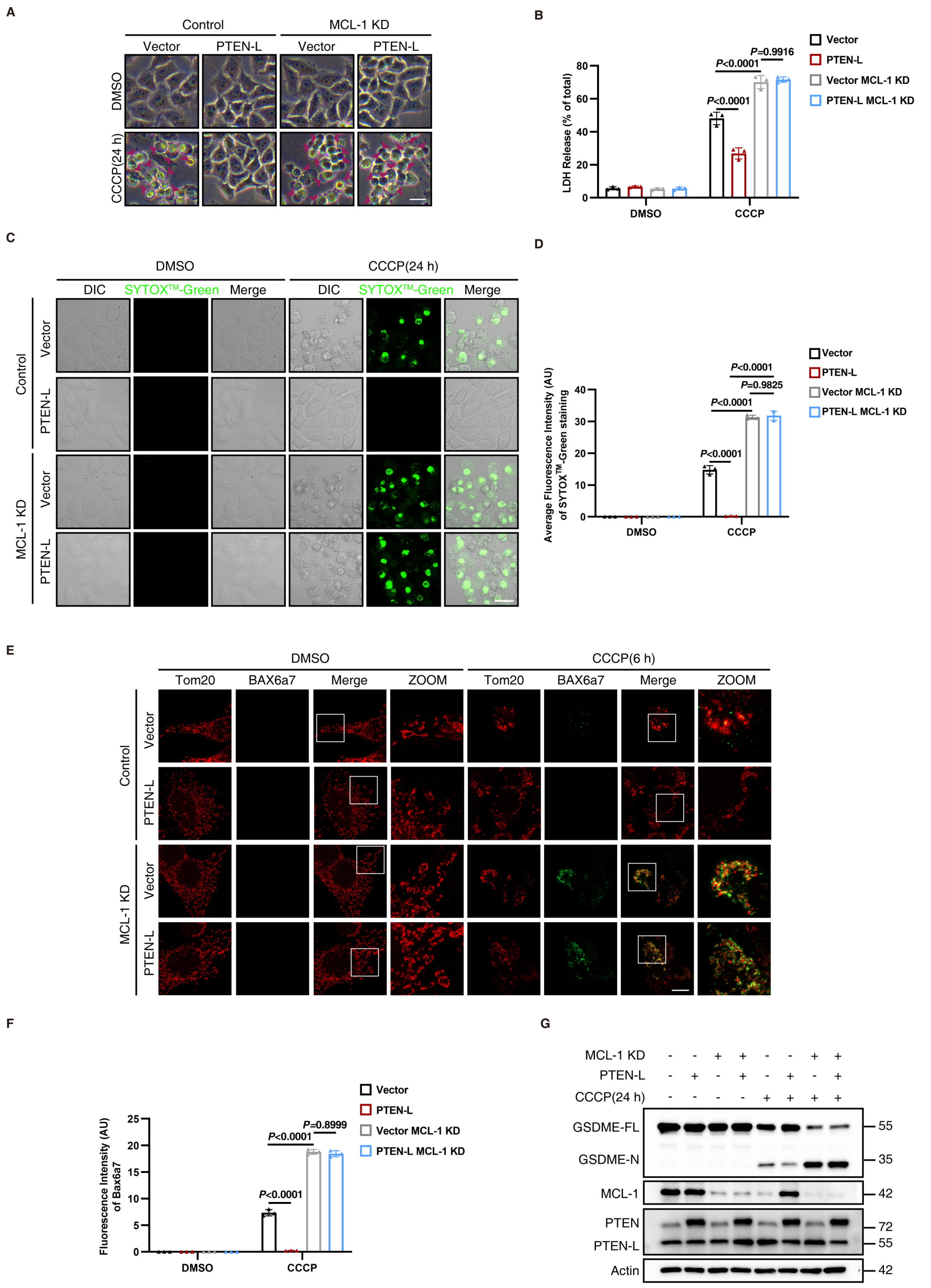

A
Control
MCL-1 KD
Vector
PTEN-L
Vector
PTEN-L
DMSO
CCCP(24 h)
B
LDH Release (% of total)
P=0.9916
P<0.0001
P<0.0001
DMSO
CCCP
Vector
PTEN-L
Vector MCL-1 KD
PTEN-L MCL-1 KD
C
DMSO
CCCP(24 h)
DIC
SYTOX™-Green
Merge
Control
MCL-1 KD
Vector
PTEN-L
D
Average Fluorescence Intensity (AU) of SYTOX™-Green staining
P<0.0001
P=0.9825
P<0.0001
P<0.0001
DMSO
CCCP
E
DMSO
CCCP(6 h)
Tom20
BAX6a7
Merge
ZOOM
F
Fluorescence Intensity (AU) of Bax6a7
P=0.8999
P<0.0001
P<0.0001
G
MCL-1 KD
PTEN-L
CCCP(24 h)
GSDME-FL
GSDME-N
MCL-1
PTEN
PTEN-L
Actin
55
35
42
72
55
42

**Appendix Figure S12. MCL-1 knockdown induces pyroptosis following mitochondrial depolarization.** (**A** and **B**) Representative microscopy images and LDH release assay of HeLa YFP-Parkin Vector/PTEN-L cells with MCL-1 knockdown (MCL-1 KD) following treatment with 20 μM CCCP for 24 h. Scale bar, 25 μm. (**C** and **D**) Representative images and average fluorescence intensity (AU) of SYTOX Green staining in HeLa YFP-Parkin Vector/PTEN-L cells with MCL-1 knockdown (MCL-1 KD) following treatment with 20 μM CCCP for 24 h. Scale bar, 20 μm. (**E** and **F**) Immunostaining assay and quantitative analysis to assess the presence of active BAX (BAX6a7) on mitochondria of HeLa YFP-Parkin Vector/PTEN-L cells with MCL-1 knockdown (MCL-1 KD) following treatment with 20 μM CCCP for 6 h. Scale bar, 5 μm. (**G**) Immunoblotting of GSDME cleavage and activation in lysates of HeLa YFP-Parkin Vector/PTEN-L cells with MCL-1 knockdown (MCL-1 KD) following treatment with 20 μM CCCP for 24 h. Red arrows in (**A**) indicate pyroptotic cells. Immunoblots in (**G**) are representative of three independent biological replicates. Data in (**B**), (**D**), and (**F**) are presented as mean ± SD from three independent biological replicates. Statistical significance was assessed by two-way ANOVA.

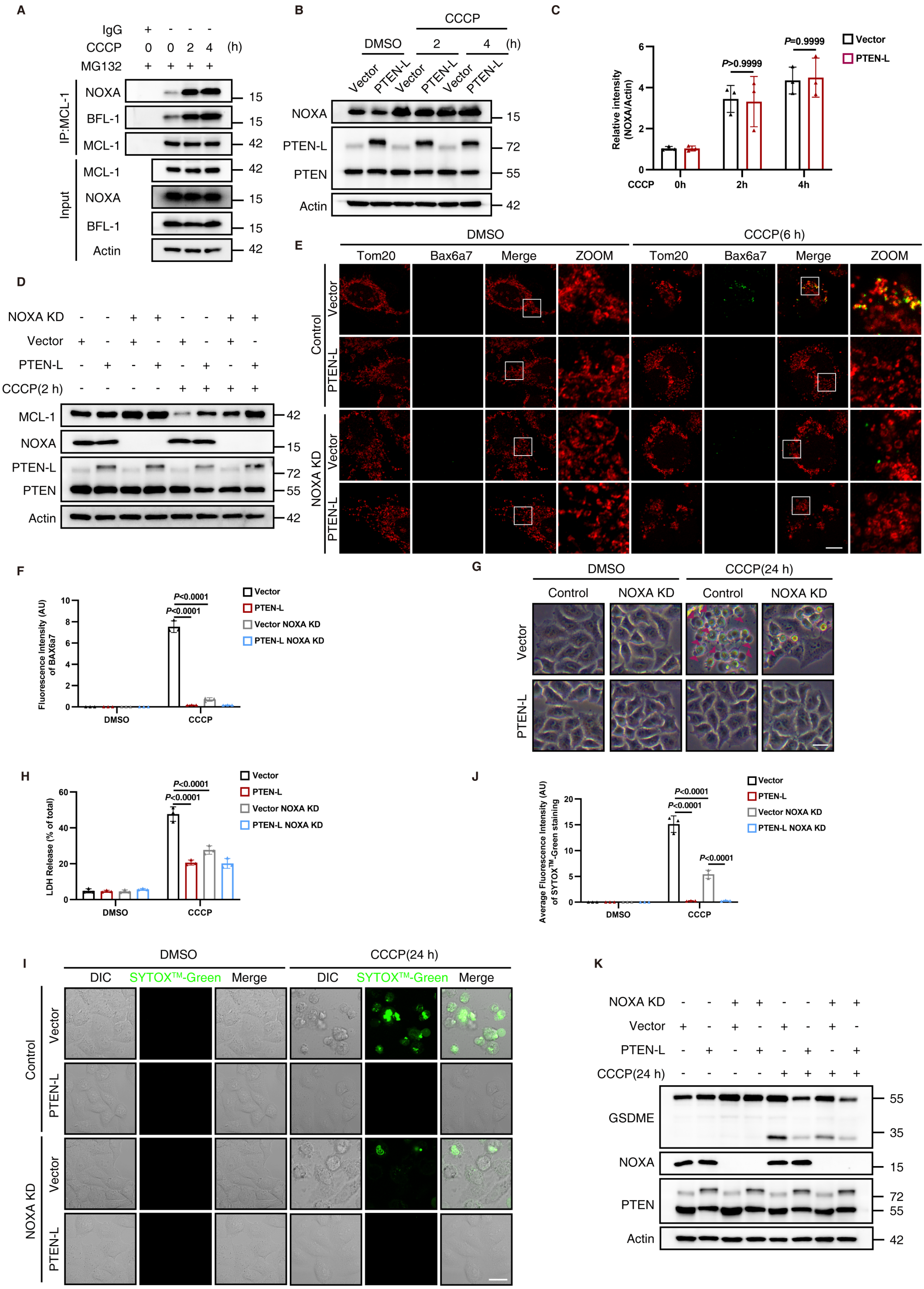
A
IgG
CCCP
MG132
(h)
IP:MCL-1
Input
NOXA
BFL-1
MCL-1
Actin
B
CCCP
DMSO
Vector
PTEN-L
NOXA
PTEN-L
PTEN
Actin
C
Relative intensity (NOXA/Actin)
P>0.9999
P=0.9999
Vector
PTEN-L
CCCP
0h
2h
4h
D
NOXA KD
Vector
PTEN-L
CCCP(2 h)
MCL-1
NOXA
PTEN-L
PTEN
Actin
E
DMSO
CCCP(6 h)
Tom20
Bax6a7
Merge
ZOOM
Control
NOXA KD
F
Fluorescence Intensity (AU) of BAX6a7
P<0.0001
Vector NOXA KD
PTEN-L NOXA KD
DMSO
CCCP
G
CCCP(24 h)
H
LDH Release (% of total)
J
Average Fluorescence Intensity (AU) of SYTOX™-Green staining
I
DIC
SYTOX™-Green
K
GSDME

**Appendix Figure S13. NOXA knockdown inhibits MCL-1 degradation and reduces CCCP-induced pyroptosis.** (**A**) Immunoprecipitation analysis to detect the endogenous MCL-1, NOXA, and BFL-1 interaction in HeLa YFP-Parkin cells pretreated with 10 μM MG132 for 1 h following cotreatment with 20 μM CCCP for 2 h or 4 h. (**B** and **C**) Immunoblotting and quantitative analysis of NOXA level in lysates of HeLa YFP-Parkin Vector/PTEN-L cells following treatment with 20 μM CCCP for 2 h or 4 h. (**D**) Immunoblotting assay of MCL-1 level in lysates of HeLa YFP-Parkin Vector/PTEN-L cells with NOXA knockdown (NOXA KD) following treatment with 20 μM CCCP for 2 h. (**E** and **F**) Immunostaining assay and quantitative analysis to assess the presence of active BAX (BAX6a7) on mitochondria of HeLa YFP-Parkin Vector/PTEN-L cells with NOXA knockdown (NOXA KD) following treatment with 20 μM CCCP for 6 h. Scale bar, 5 μm. (**G** and **H**) Representative microscopy images and LDH release assay in lysates of HeLa YFP-Parkin Vector/PTEN-L cells with NOXA knockdown (NOXA KD) following treatment with 20 μM CCCP for 24 h. Scale bar, 25 μm. (**I** and **J**) Representative images and average fluorescence intensity (AU) of SYTOX Green staining in HeLa YFP-Parkin Vector/PTEN-L cells with NOXA knockdown (NOXA KD) following treatment with 20 μM CCCP for 24 h. Scale bars, 20 μm. (**K**) Immunoblotting analysis of GSDME cleavage and activation in lysates of HeLa YFP-Parkin Vector/PTEN-L cells with NOXA knockdown (NOXA KD) following treatment with 20 μM CCCP for 24 h. Red arrows in (**G**) indicate pyroptotic cells. Immunoblots in (**A**) are representative of two independent biological replicates. Immunoblots in (**B**)

and (**D**) are representative of three independent biological replicates. Immunoblots in (**K**) are representative of four independent biological replicates. Data in (**C**), (**F**), (**H**), and (**J**) are presented as mean ± SD from three independent biological replicates. Statistical significance was assessed by two-way ANOVA.

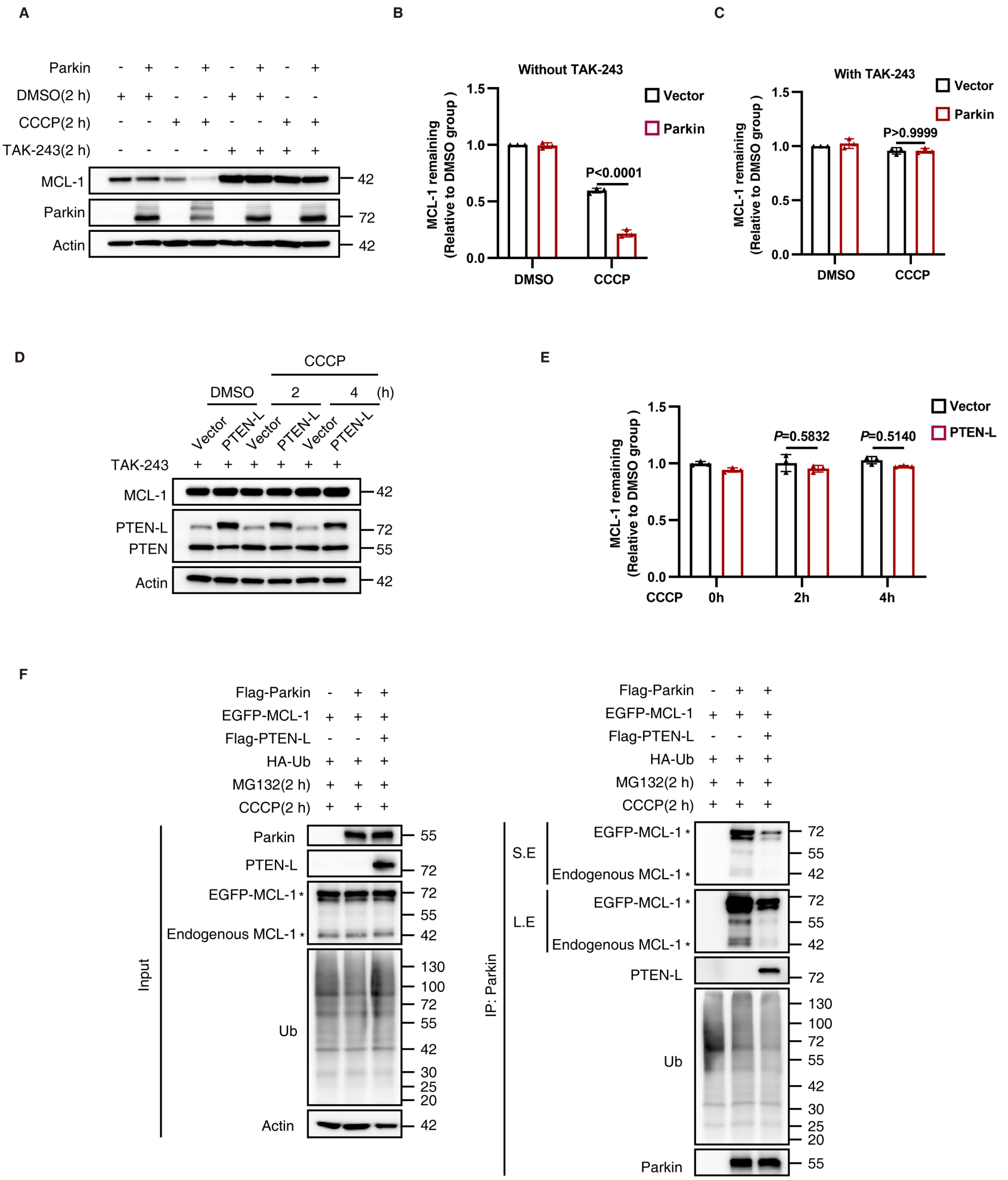
A
Parkin - + - + - + - +
DMSO(2 h) + + - - + + - -
CCCP(2 h) - - + + - - + +
TAK-243(2 h) - - - - + + + +
MCL-1
Parkin
Actin
42
72
42
B
Without TAK-243
MCL-1 remaining
(Relative to DMSO group )
Vector
Parkin
P<0.0001
DMSO
CCCP
C
With TAK-243
MCL-1 remaining
(Relative to DMSO group )
Vector
Parkin
P>0.9999
DMSO
CCCP
D
CCCP
DMSO
2
4
(h)
Vector
PTEN-L
Vector
PTEN-L
Vector
PTEN-L
TAK-243 + + + + + +
MCL-1
PTEN-L
PTEN
Actin
42
72
55
42
E
MCL-1 remaining
(Relative to DMSO group )
Vector
PTEN-L
P=0.5832
P=0.5140
CCCP
0h
2h
4h
F
Flag-Parkin - + +
EGFP-MCL-1 + + +
Flag-PTEN-L - - +
HA-Ub + + +
MG132(2 h) + + +
CCCP(2 h) + + +
Input
Parkin
PTEN-L
EGFP-MCL-1 *
Endogenous MCL-1 *
Ub
Actin
IP: Parkin
S.E
L.E
EGFP-MCL-1 *
Endogenous MCL-1 *
PTEN-L
Ub
Parkin
130
100
72
55
42
30
25
20

**Appendix Figure S14. TAK-243 completely blocks CCCP-induced MCL-1 degradation.** (**A** to **C**) Immunoblotting and quantitative analysis of MCL-1 level in lysates of HeLa WT/YFP-Parkin cells following treatment with 20 μM CCCP, or 20 μM CCCP plus 20 μM TAK243 for 2 h. (**D** and **E**) Immunoblotting and quantitative analysis of MCL-1 level in lysates of HeLa YFP-Parkin Vector/PTEN-L cells following treatment with 20 μM TAK243 and 20 μM CCCP for 2 h or 4 h. (**F**) Co-immunoprecipitation analysis of the interaction between Parkin, and PTEN-L, Ub or MCL-1 in HEK293T cells transiently co-transfected with EGFP-MCL-1, HA-Ub, Flag-Parkin, Flag-PTEN-L and empty vector constructs pretreated with 10 μM MG132 for 2 h following cotreatment with 20 μM CCCP for 2 h. Immunoblots in (**F**) are representative of two independent biological replicates. Immunoblots in (**A**) and (**D**) are representative of three independent biological replicates. Data in (**B**), (**C**), and (**E**) are presented as mean ± SD from three independent biological replicates. Statistical significance was assessed by two-way ANOVA.

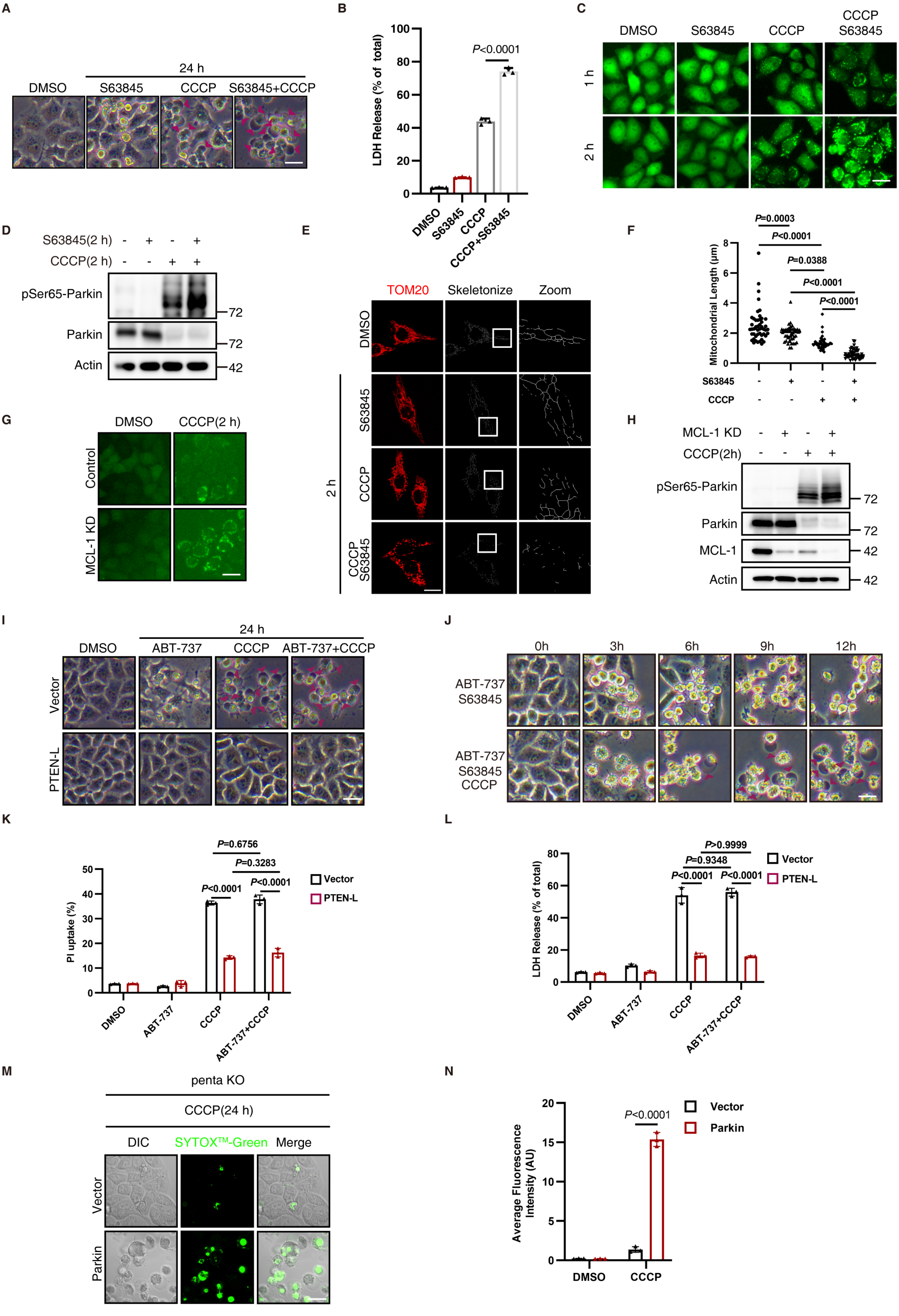
A
24 h
DMSO
S63845
CCCP
S63845+CCCP
B
LDH Release (% of total)
P<0.0001
DMSO
S63845
CCCP
CCCP+S63845
C
DMSO
S63845
CCCP
CCCP S63845
1 h
2 h
D
S63845(2 h)
CCCP(2 h)
pSer65-Parkin
Parkin
Actin
72
72
42
E
TOM20
Skeletonize
Zoom
DMSO
S63845
CCCP
CCCP S63845
2 h
F
Mitochondrial Length (μm)
P=0.0003
P<0.0001
P=0.0388
P<0.0001
P<0.0001
S63845
CCCP
G
DMSO
CCCP(2 h)
Control
MCL-1 KD
H
MCL-1 KD
CCCP(2h)
pSer65-Parkin
Parkin
MCL-1
Actin
72
72
42
42
I
24 h
DMSO
ABT-737
CCCP
ABT-737+CCCP
Vector
PTEN-L
J
0h
3h
6h
9h
12h
ABT-737 S63845
ABT-737 S63845 CCCP
K
PI uptake (%)
P=0.6756
P=0.3283
P<0.0001
P<0.0001
Vector
PTEN-L
DMSO
ABT-737
CCCP
ABT-737+CCCP
L
LDH Release (% of total)
P>0.9999
P=0.9348
P<0.0001
P<0.0001
Vector
PTEN-L
DMSO
ABT-737
CCCP
ABT-737+CCCP
M
penta KO
CCCP(24 h)
DIC
SYTOX™-Green
Merge
Vector
Parkin
N
Average Fluorescence Intensity (AU)
P<0.0001
Vector
Parkin
DMSO
CCCP

**Appendix Figure S15. MCL-1 inhibition enhances CCCP-induced pyroptosis, facilitates Parkin translocation to mitochondria and its activation, and aggravates mitochondrial damage.** (**A** and **B**) Representative microscopy images and LDH release assay of HeLa YFP-Parkin cells treated with 3 μM S63845, 20 μM CCCP or 3 μM S63845 plus 20 μM CCCP for 24 h. Scale bar, 25 μm. (**C**) Representative microscopy images of Parkin translocation in HeLa YFP-Parkin cells treated with 3 μM S63845, 20 μM CCCP or 3 μM S63845 plus 20 μM CCCP for 1 h or 2 h. Scale bar, 25 μm. (**D**) Immunoblotting analysis of pSer65-Parkin levels in lysates of HeLa YFP-Parkin cells treated with 3 μM S63845, 20 μM CCCP or 3 μM S63845 plus 20 μM CCCP for 2 h. (**E** and **F**) Immunostaining assay and its quantitative analysis to detect mitochondrial damage of HeLa YFP-Parkin cells following treatment with 3 μM S63845, 20 μM CCCP or 3 μM S63845 plus 20 μM CCCP for 2 h. Scale bar, 10μm. (**G**) Representative microscopy images of Parkin translocation in HeLa YFP-Parkin cells with MCL-1 knockdown (MCL-1 KD) treated with 20 μM CCCP for 2 h. Scale bar, 25 μm. (**H**) Immunoblotting analysis of pSer65-Parkin levels in lysates of HeLa YFP-Parkin cells with MCL-1 knockdown (MCL-1 KD) treated with 20 μM CCCP for 2 h. (**I**) Representative microscopy images of HeLa YFP-Parkin Vector/PTEN-L cells following treatment with 5 μM ABT-737, 20 μM CCCP, or 5 μM ABT-737 plus 20 μM CCCP for 24 h. Scale bar, 25 μm. (**J**) Representative microscopy images of HeLa cells stably expressing YFP-Parkin treated with 1 μM S63845/1 μM ABT-737 in combination with or without 20 μM CCCP for expected time. Scale bar, 25 μm. (**K** and **L**) PI uptake and LDH

release assay of HeLa YFP-Parkin Vector/PTEN-L cells following treatment with 5 μM ABT-737, 20 μM CCCP, or 5 μM ABT-737 plus 20 μM CCCP for 24 h. (**M** and **N**) Representative images and avarage fluorescence intensity (AU) of SYTOX Green staining in HeLa WT/penta KO with Parkin overexpressing cells following treatment with 20 μM CCCP for 24 h. Scale bars, 20 μm. Red arrows in (**A**), (**I**) and (**J**) indicate pyroptotic cells. Immunoblots in (**D**) and (**H**) are representative of three independent biological replicates. Data in (**B**), (**F**), (**K**), (**L**) and (**N**) are presented as mean ± SD from three independent biological replicates. Statistical significance were assessed by two-way ANOVA for (**F**), (**K**), (**L**), (**N**) and assessed by two-tailed unpaired Student's-t test for (**B**).

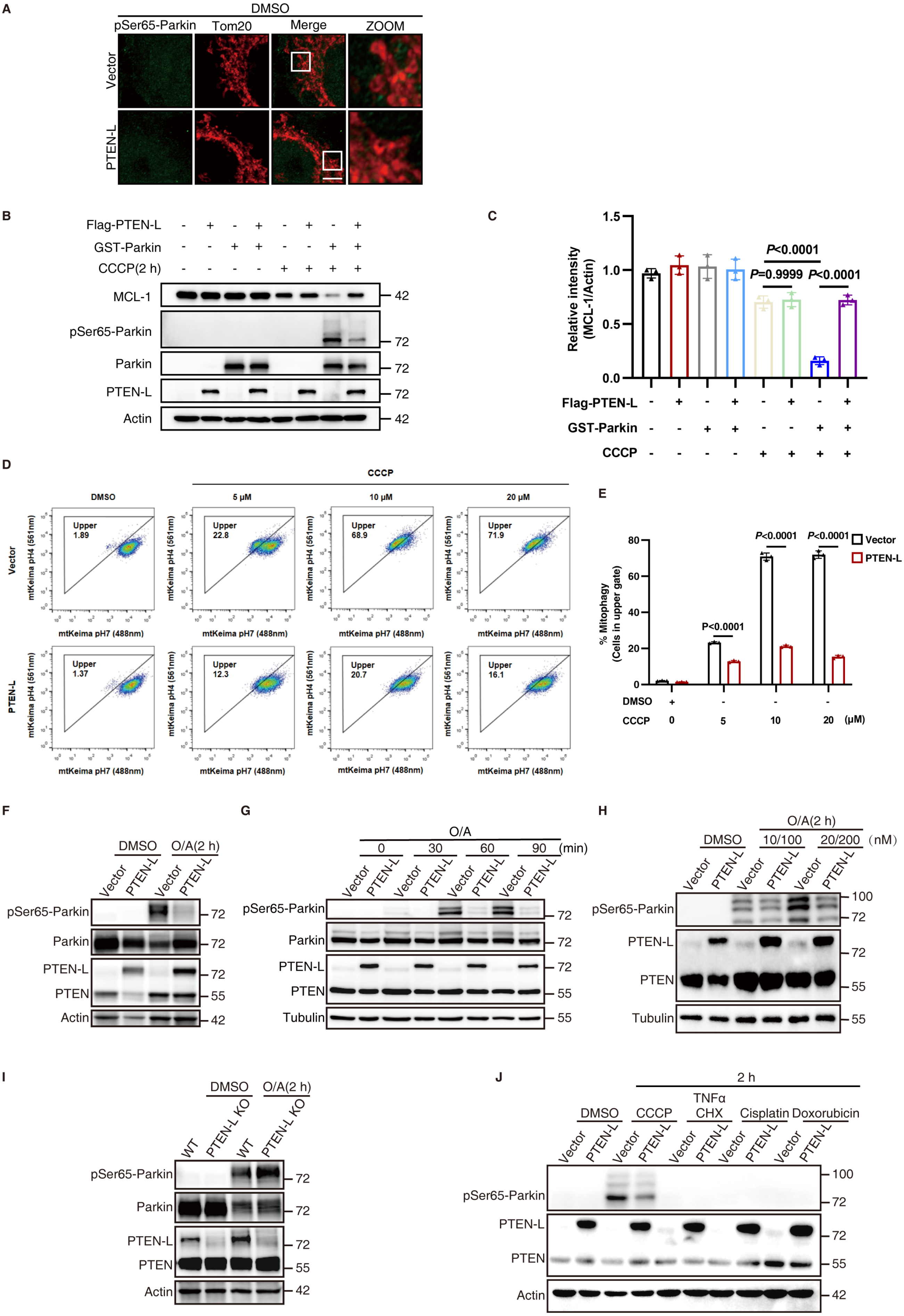
A
DMSO
pSer65-Parkin
Tom20
Merge
ZOOM
Vector
PTEN-L
B
Flag-PTEN-L
GST-Parkin
CCCP(2 h)
MCL-1
pSer65-Parkin
Parkin
PTEN-L
Actin
42
72
C
Relative intensity (MCL-1/Actin)
P<0.0001
P=0.9999
P<0.0001
Flag-PTEN-L
GST-Parkin
CCCP
D
DMSO
CCCP
5 μM
10 μM
20 μM
Vector
PTEN-L
mtKeima pH4 (561nm)
mtKeima pH7 (488nm)
Upper 1.89
Upper 22.8
Upper 68.9
Upper 71.9
Upper 1.37
Upper 12.3
Upper 20.7
Upper 16.1
E
% Mitophagy (Cells in upper gate)
P<0.0001
Vector
PTEN-L
DMSO
CCCP
(μM)
F
DMSO
O/A(2 h)
Vector
PTEN-L
pSer65-Parkin
Parkin
PTEN-L
PTEN
Actin
G
O/A
(min)
Tubulin
H
O/A(2 h)
10/100
20/200
(nM)
I
WT
PTEN-L KO
J
2 h
CCCP
TNFα CHX
Cisplatin
Doxorubicin

**Appendix Figure S16. PTEN-L reduces pSer65-Parkin and inhibits mitophagy.** (**A**) Immunostaining assay for pSer65-Parkin in HeLa YFP-Parkin Vector/PTEN-L cells, as control of **Figure 6C**. Scale bar, 5 μm. (**B** and **C**) Immunoblotting and quantitative analysis of MCL-1 level in lysates of HeLa WT cells transiently co-transfected with Flag-PTEN-L, GST-Parkin, and empty vector constructs following treated with 20 μM CCCP for 2 h. (**D** and **E**) The detection of mitophagy using flow cytometry and quantitative analysis of HeLa YFP-Parkin Vector/PTEN-L cells stably expressing mt-Keima treated with 5 μM, 10 μM or 20 μM CCCP combined with 40 μM Z-VAD for 24 h. (**F**) Immunoblotting analysis for pSer65-Parkin levels of HeLa YFP-Parkin Vector/PTEN-L cell lysates following treatment with 20 nM/200 nM O/A for 2 h. (**G**) Immunoblotting analysis for pSer65-Parkin levels in HeLa YFP-Parkin Vector/PTEN-L cell lysates treated with 20 nM/200 nM O/A for indicated timepoints. (**H**) Immunoblotting analysis for pSer65-Parkin levels in HeLa YFP-Parkin Vector/PTEN-L cells following treatment with 10 nM/100 nM or 20 nM/200 nM O/A for 2 h. (**I**) Immunoblotting analysis for pSer65-Parkin levels of HeLa YFP-Parkin WT/PTEN-L KO cell lysates following treatment with 20 nM/200 nM O/A for 2 h. (**J**) Immunoblotting analysis for pSer65-Parkin levels of HeLa YFP-Parkin Vector/PTEN-L cell lysates following treatment with 20 μM CCCP, 20 ng/mL TNFα plus 10 ug/mL CHX, 20 μg/mL Cisplatin or 1 μM Doxorubicin for 2 h. Immunoblots in (**F**), (**G**), (**H**), (**I**), and (**J**) are representative of two independent biological replicates. Immunoblots in (**B**) are representative of three independent biological replicates. Data in (**C**) and (**E**) are

presented as mean ± SD from three independent biological replicates. Statistical significance was assessed by two-tailed unpaired Student's-t test (**C**), or two-way ANOVA (**E**).

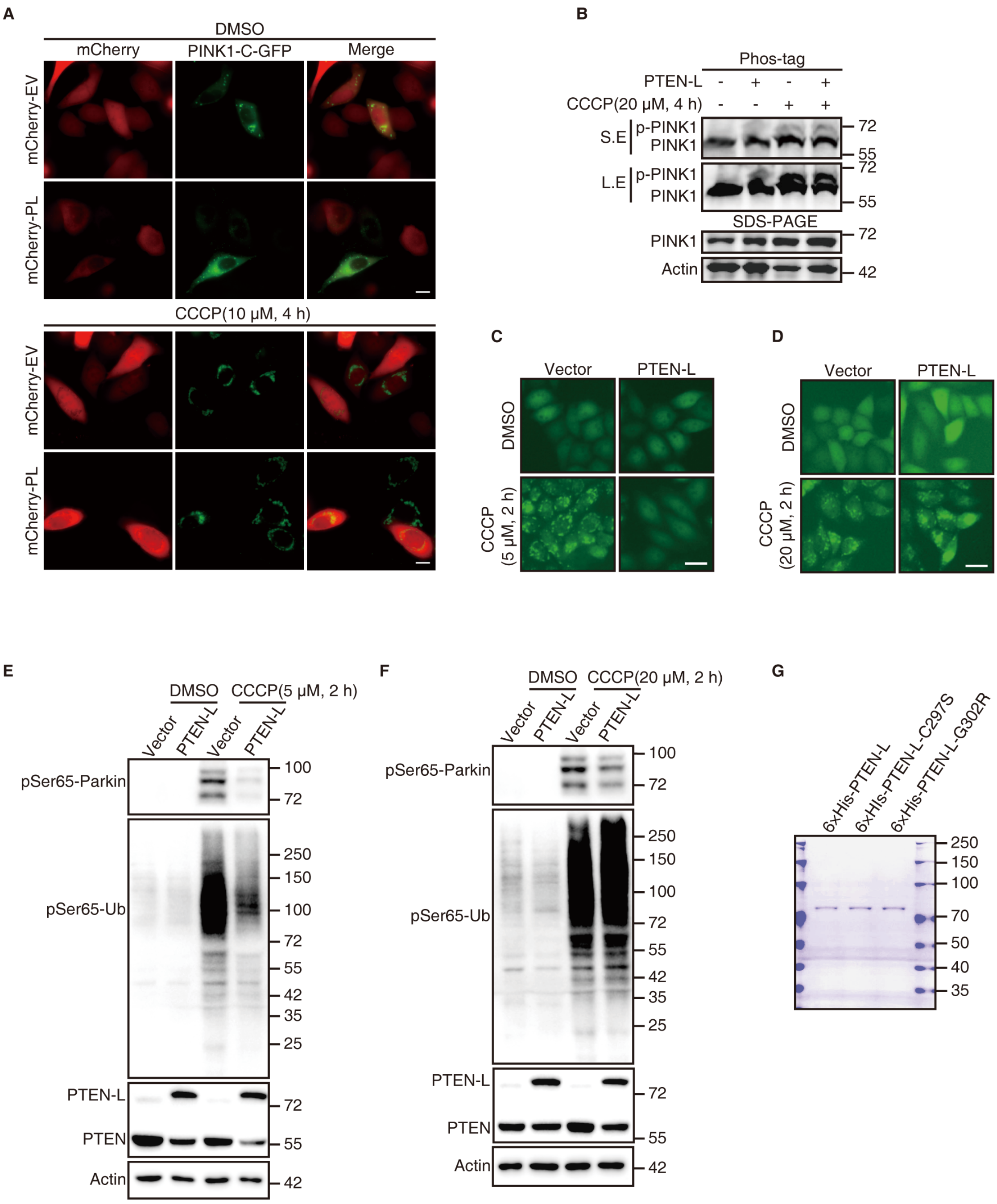

A
DMSO
mCherry
PINK1-C-GFP
Merge
mCherry-EV
mCherry-PL
CCCP(10 μM, 4 h)
B
Phos-tag
PTEN-L
CCCP(20 μM, 4 h)
S.E
L.E
p-PINK1
PINK1
SDS-PAGE
Actin
C
D
Vector
PTEN-L
DMSO
CCCP (5 μM, 2 h)
CCCP (20 μM, 2 h)
E
F
CCCP(5 μM, 2 h)
CCCP(20 μM, 2 h)
pSer65-Parkin
pSer65-Ub
PTEN
G
6xHis-PTEN-L
6xHis-PTEN-L-C297S
6xHis-PTEN-L-G302R

**Appendix Figure S17. The reduction effects of PTEN-L on pSer65-Parkin are independent of PINK1 activity, Parkin mitochondrial distribution and pSer65-Ub.** (**A**) Representative microscopy images of HeLa HA-Parkin cells transiently transfected with the PINK1-C-GFP, mCherry empty control (mCherry-EV) or mCherry-PTEN-L (mCherry-PL) constructs following treatment with 10 μM CCCP for 4 h. Scale bar, 10 μm. (**B**) Immunoblotting analysis for p-PINK1 levels by Phos-tag assay of HeLa YFP-Parkin Vector/PTEN-L cell lysates following treatment with 20 μM CCCP for 4 h. SDS-PAGE was performed concurrently. PINK1 antibodies were used for PINK1 and p-PINK1 detection. S.E, short exposure. L.E, long exposure. (**C** and **D**) Representative microscopy images for Parkin translocation in HeLa YFP-Parkin Vector/PTEN-L cells treated with 5 μM CCCP or 20 μM CCCP for 2 h. Scale bar, 25 μm. (**E** and **F**) Immunoblotting analysis for pSer65-Parkin and pSer65-Ub levels of HeLa YFP-Parkin Vector/PTEN-L cell lysates following treatment with 5 μM CCCP or 20 μM CCCP for 2 h. (G) SDS-PAGE gel and Coomassie Blue staining for purified 6×His-WT PTEN-L, 6×His-PTEN-L$^{C297S}$ and 6×His-PTEN-L$^{G302R}$ proteins from *E. coli*. Immunoblots in (**B**), (**E**), (**F**), and (**G**) are representative of three independent biological replicates.
